# *De Novo* Multi‐Mechanism Antimicrobial Peptide Design via Multimodal Deep Learning

**DOI:** 10.1002/advs.202515835

**Published:** 2026-03-09

**Authors:** Xiaojuan Li, Haifan Gong, Yue Wang, Yinuo Zhao, Lixiang Li, Peijing Bao, Qingzhou Kong, Jialu Fu, Boyao Wan, Yumeng Zhang, Jinghui Zhang, Jiekun Ni, Zhongxue Han, Xueping Nan, Kunping Ju, Longfei Sun, Yuerui Ma, Huijun Chang, Mengqi Zheng, Yanbo Yu, Xiaoyun Yang, Xiuli Zuo, Haina Wang, Yanqing Li

**Affiliations:** ^1^ Department of Gastroenterology Qilu Hospital of Shandong University Jinan China; ^2^ School of Science and Engineering Chinese University of Hong Kong Shenzhen China; ^3^ Shandong Provincial Microecological Research and Biotherapy Center Jinan China; ^4^ Shandong Provincial Clinical Research Center for digestive disease Jinan China; ^5^ School of Chemistry and Chemical Engineering Nanjing University of Science and Technology Nanjing China; ^6^ School of Life Sciences and Biotechnology Shanghai Jiao Tong University Shanghai China; ^7^ School of Pharmaceutical Sciences Shandong University Jinan Shandong China

**Keywords:** 3D structure, antimicrobial peptide, *de novo* design, multi‐mechanism antimicrobial peptide, multimodal deep learning

## Abstract

Artificial intelligence (AI)‐driven discovery of antimicrobial peptides (AMPs) is yet to fully utilize their three‐dimensional (3D) structural characteristics, microbial species‐specific antimicrobial activities, and mechanisms. Here, we constructed a QLAPD database comprising the sequence, structures, and antimicrobial properties of 12 914 AMPs. QLAPD underlies a multimodal, multitask, multilabel, and conditionally controlled AMP discovery (M3‐CAD) pipeline, proposed for the *de novo* design of multi‐mechanism AMPs to combat multidrug‐resistant organisms (MDROs). This pipeline integrates generation, regression, and classification modules, using an innovative 3D voxel coloring method to capture the nuanced physicochemical context of amino acids, thus enhancing structural characterizations. QLX‐3DV‐1 and QLX‐3DV‐2, identified through M3‐CAD, were found to demonstrate multiple antimicrobial mechanisms, notable activity against MDROs, and low toxicity. In vivo experiments were used to validate their antimicrobial effects with limited local and systemic toxicity. Overall, integrating 3D features, species‐specific antimicrobial activities, and mechanisms enhanced AI‐driven AMP discovery, making the M3‐CAD pipeline a viable tool for *de novo* AMP design.

## Graphical abstract

Current AI‐driven peptide discovery often overlooks complex structural data. This study presents M3‐CAD, a generative pipeline that leverages 3D voxel coloring and a massive database of over 12 000 peptides to capture nuanced physicochemical contexts. This innovative framework enables the precise design of multi‐mechanism antimicrobial peptides that effectively clear multidrug‐resistant infections while maintaining high safety profiles.

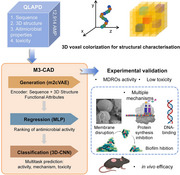

## 1 Introduction

The escalating threat from multidrug‐resistant organisms (MDROs) to human health necessitates an urgent search for new antimicrobial solutions [1, 2]. As antimicrobial resistance (AMR) affects all clinically used antibiotics, developing substitutes with multiple novel mechanisms is crucial [3, 4, 5, 6].

Antimicrobial peptides (AMPs) are key players in innate immunity and provide primary defense against pathogens [7, 8]. Their amphiphilic and positively charged nature allows selective disruption of negatively charged bacterial membranes while sparing the neutral mammalian cell membranes [9, 10]. Beyond membrane disruption, certain AMPs can penetrate bacterial membranes and disrupt fundamental cellular processes, including DNA replication, transcription, translation, protein synthesis, protein folding, and cell division [11]. Further, AMPs inhibit bacterial biofilms through diverse mechanisms; these include disrupting biofilm matrix components, interfering with quorum‐sensing pathways, and targeting intracellular processes like the stringent‐stress response, which are critical for biofilm formation and maintenance [12]. These nonspecific antimicrobial activities, which are primarily driven by biophysical interactions with the bacterial membrane and multiple intracellular targets [13, 14, 15], reduce the likelihood of antimicrobial resistance development, positioning AMPs as promising candidates for next‐generation therapeutics and viable alternatives to conventional antibiotics [16].

However, traditional AMP discovery using wet experiments is time‐consuming and expensive [17, 18]. Consequently, artificial intelligence (AI)‐driven approaches for identification [19, 20, 21, 22, 23], optimization [24, 25, 26], and generation [18, 27, 28, 29, 30] have been proposed to accelerate AMP discovery. Among these, generative models overcome the limitations of identification and optimization models. Unlike identification models, which screen targets within a defined peptide range, and optimization models, which require manual selection of template sequences, generative models theoretically yield optimal AMPs that meet the specific target conditions.

All these AI‐driven AMP discovery models require large‐scale, manually labeled data for empowerment. Driven by the significance of training data, the following question arises: What human knowledge and peptide features can be used to enhance the inhibitory activity of AMPs discovered by AI, particularly generative AI, against superbugs?

In this work, we revealed the following findings. First, AI‐driven AMP discovery currently utilizes limited human knowledge, primarily regarding whether a peptide exhibits antibacterial properties, and often neglects the varying efficacy of AMPs against distinct bacteria, particularly clinically isolated MDROs [31]. Second, most existing studies assume that AMPs function by binding to and disrupting negatively charged bacterial membranes, thereby adopting membrane disruption‐related features in AMP discovery frameworks [19, 23] and verifying the membrane permeabilization and depolarization abilities of AMPs through experimental methods [18, 19, 20, 21, 22, 23, 26, 29, 30]. This has resulted in other antimicrobial mechanisms, including inhibition of biofilm formation, DNA replication, and protein synthesis, being overlooked during the design phase. Finally, existing studies rely heavily on primary sequence data and neglecting crucial 3D structural information that reflects secondary and tertiary structures, which hold major significance in peptide function [31]. However, recent advancements in AI models such as AlphaFold2 and RoseTTAFold have improved the precision of peptide and protein structure prediction [32, 33], facilitating enhanced peptide characterization and downstream tasks using 3D structural data.

We addressed these gaps and curated a comprehensive AMP database (QLAPD) by meticulously selecting 12 914 AMP entries from existing knowledge bases [34, 35, 36, 37] and numerous published papers. Each entry records eight feature categories, including the predicted 3D structure, microbial species‐specific antimicrobial activities, antimicrobial mechanisms, and toxicity. We propose a multimodal, multitask, multilabel, and conditionally controlled AMP discovery (M3‐CAD) pipeline to generate and optimize new AMPs with multiple antimicrobial mechanisms, broad‐spectrum resistance to MDROs, and low host toxicity. We also introduce a 3D voxel coloring method and a multilabel rebalancing training strategy to address the challenges in representing 3D peptide structures and the long‐tail distribution problem in multilabel classification tasks.

Wet lab validation of the M3‐CAD‐selected candidate sequences demonstrated their inhibitory abilities against 25 ESKAPE (*E. faecium*, *S. aureus*, *K. pneumoniae*, *A. baumannii*, *P. aeruginosa*, and *Enterobacter* species) [38] MDROs. The most efficacious AMPs, QLX‐3DV‐1 and QLX‐3DV‐2, displayed potent antimicrobial activity, low host toxicity, and multiple antimicrobial mechanisms. Under the tested conditions, QLX‐3DV‐1 and QLX‐3DV‐2 showed no significant resistance development after 20 passages of bacterial cultivation, unlike that observed with clindamycin. QLX‐3DV‐1 and QLX‐3DV‐2 reduced the bacterial load with no observable in vivo toxicity in full‐thickness skin wound models infected with *S. aureus* and *E. coli*. Comprehensively, incorporating the 3D structural features of the peptide and the information regarding the antimicrobial mechanism enhanced AI‐driven AMP discovery, rendering the M3‐CAD pipeline a promising tool for *de novo* AMP design.

## 2 Results

### 2.1 QLAPD: A Database Linking the Sequences, Structures, and Properties of AMPs

**FIGURE 1:**
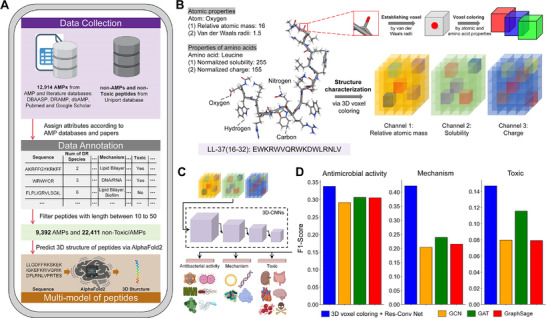
Overview of the proposed QLAPD database and 3D voxel coloring method. (A) Schematic flow chart of antimicrobial peptide collection, data cleaning, label annotation, and multimodal feature construction of the QLAPD database. (B) Schematic diagram of the 3D voxel coloring method, using the antimicrobial peptide LL‐37(16‐32) as an example. (C) The schematic diagram shows that the 3D structural features of polypeptides obtained with the 3D voxel coloring method are used to train three downstream multilabel classification tasks of the 3D‐CNN model. (D) Bar plots show the performance of the 3D voxel coloring + Res‐Conv Net and three graph neural network methods in the multilabel classification task of antimicrobial activity, mechanism, and toxicity of antimicrobial peptides. The means obtained using fivefold cross‐validation are shown.

QLAPD, an AMP database reviewed and annotated by clinical doctors and microbiology experts from Qilu Hospital, was established to train an AI framework capable of designing AMPs *de novo*, with multiple antimicrobial mechanisms and MDRO inhibition potential. QLAPD encompasses 12 914 AMPs and each records its amino acid sequence, predicted 3D structure, and six functional attributes, namely: (1) inhibitory activity against six drug‐resistant pathogenic bacteria; (2) the number of drug‐resistant bacteria it can inhibit; (3) the number of non‐resistant bacteria it can inhibit; (4) four antimicrobial mechanisms, such as disrupting bacterial membranes; (5) toxicity; and (6) nephrotoxicity among the six kinds of organ toxicity (Figure 1A; Figure S1A–F). We used a local ColabFold implementation of AlphaFold2 to predict the peptide structure. We retained the highest‐confidence prediction per peptide for voxelization. AlphaFold2 outputs five models per sequence with per‐residue pLDDT scores; we averaged the pLDDT across residues for each model and selected the one with the highest mean value. That top‐ranked model was then used to generate a 3D voxel grid, such that downstream analyses used the most reliable predicted structure. The peptide 3D structures in the QLAPD database are curated from two sources: experimental structures from the Protein Data Bank (PDB) [39]. are prioritized when available, whereas AlphaFold2‐predicted models [32] are utilized in the absence of experimental data. Each structure includes the type of each atom in the peptide, coordinates of the atom in 3D space, and the type of amino acid residue to which the atom belongs (Figure 1A).

To validate the accuracy of AlphaFold2‐predicted peptide conformations in QLAPD, we curated a benchmark dataset of 79 experimentally resolved peptides (sequence length < 50 aa) from the PDB database and compared four prediction methods: AlphaFold2 with multiple sequence alignment (MSA), AlphaFold2 without MSA, ESMFold [40], and HelixFold [41]. As shown in Table S1, AlphaFold2 with MSA outperformed all other baselines, achieving an average TM_score > 0.67. We thus applied AlphaFold2 with MSA to predict all remaining QLAPD peptide structures for downstream AI‐driven *de novo* AMP design.

### 2.2 3D Voxel Coloring to Improve Structural Characterization of the Peptides

Inspired by the solutions to 3D visual tasks, a 3D voxel coloring method was proposed to improve the 3D structural representation of peptides. A peptide was placed in a 3D Cartesian coordinate system with its centroid as the origin and housed within a cube comprising equal 3D voxels. The occupied voxels were then determined by the spatial location of atoms (predicted by AlphaFold2 [32]) and their van der Waals radii [42]. The voxels within the atom‐covered area have feature channel‐assigned values based on the properties of corresponding atoms, defined here by atomic mass, solubility (indicating hydrophobicity), and acidity or alkalinity (indicating charge). The unoccupied 3D voxels were assigned a value of zero (Figure 1B; Figure S1G and Table S2). This paradigm allowed us to adeptly convert the complex challenge of peptide 3D structural characterization into a tractable 3D visual feature extraction task and solve it by introducing 3D convolutional neural network (3D‐CNN) models (Figure 1C).

To assess this method, we compared the performance of the 3D‐CNN, based on the 3D voxel coloring method (3D Res‐Conv Net [43], 3D SwinUNETR Net [44]), against traditionally used Graph Neural Networks (GNNs) [45], such as GCN [46], GAT [47], and GraphSage [48], to achieve multilabel classification of peptide antimicrobial activity, mechanisms, and toxicity. The fivefold cross‐validation results indicate that compared with GNNs, the proposed 3D‐CNN models, particularly the 3D Res‐Conv Net, significantly improve the F1‐score on the antimicrobial activity prediction task; the AP, F1‐score, and ACC on the toxicity classification task; and the F1‐score and area under the curve (AUC) on the mechanism prediction task, all without materially sacrificing other metrics, thereby achieving the highest overall score across all tasks (Figure 1D; Tables S3–S5). We also compared this method with several conventional methods (SVM, XGBoost, and CATBoost) using the one‐hot sequence embedding as the input feature (Table S6).

### 2.3 M3‐CAD Pipeline Design

**FIGURE 2:**
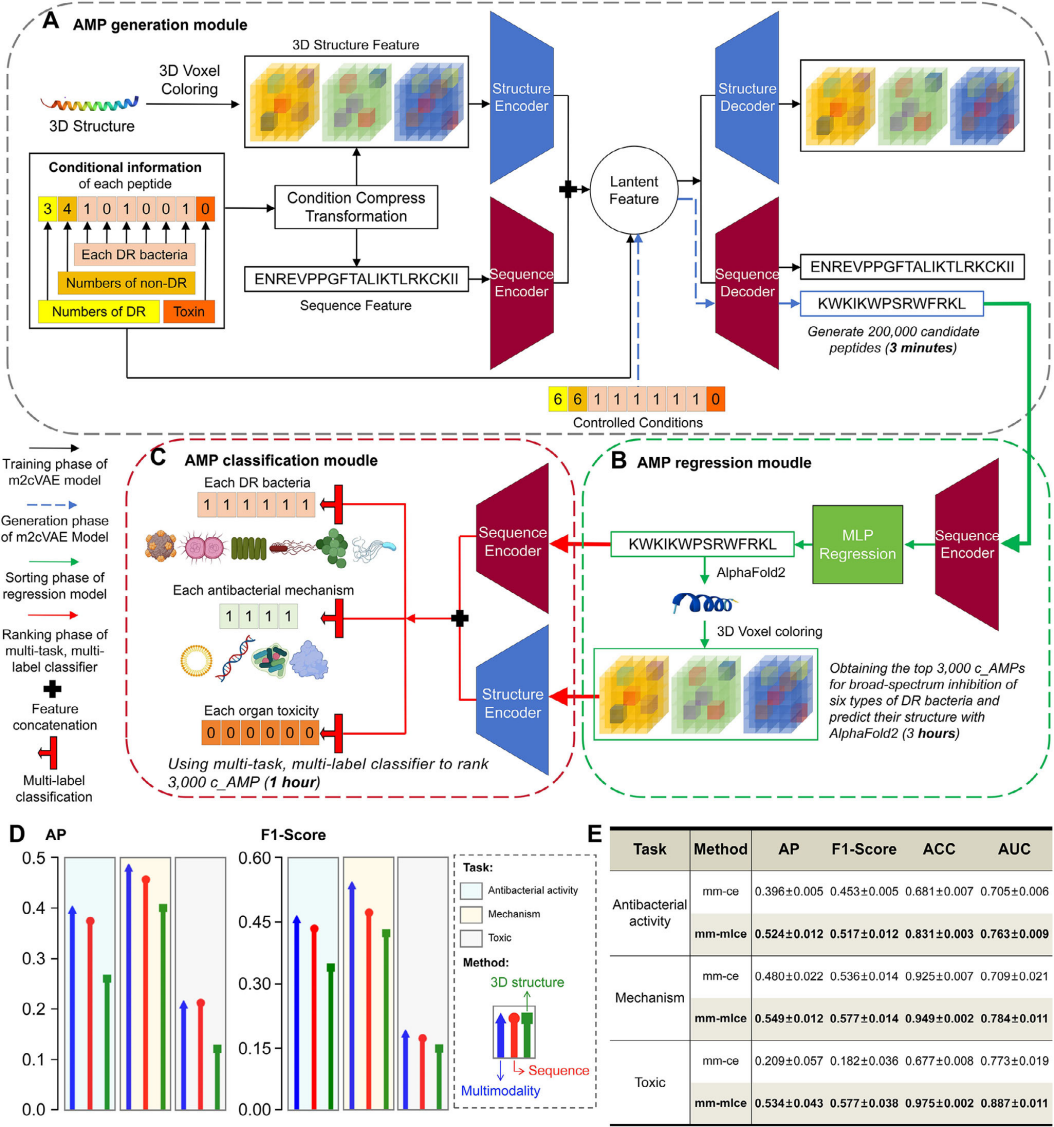
Workflow and performance evaluation of the M3‐CAD pipeline. (A–C) Schematic showing the generation (A), regression (B), and classification (C) modules that constitute the M3‐CAD pipeline. (D) The lollipop plot demonstrates the performance of multimodality‐based models and models based only on sequence or 3D structural modalities on the multilabel classification task of antimicrobial activity, mechanism, and toxicity. Means obtained by fivefold cross‐validation are shown. (E) The table shows the performance improvement of three downstream tasks of AMPs by applying the multilabel rebalancing loss function (mm‐mlce). Means and standard deviations obtained by fivefold cross‐validation are shown.

We proposed an M3‐CAD pipeline comprising sequential generation, regression, and classification modules (Figure 2A–C). Compared with the methods reported in previous studies, the greatest advantage of M3‐CAD is that it uses the antimicrobial mechanism data and inhibitory activity data of AMPs against MDROs during training, thereby learning the potential relationship between the sequence and structure of the peptides and their functional attributes, including the four antimicrobial mechanisms and inhibitory ability against six types of drug‐resistant bacteria.

The training and cross‐validation data for M3‐CAD included 9392 AMPs with lengths of 10–50 amino acid residues selected from the QLAPD database and 22 411 non‐AMPs of the same length range collected from the UniProt database [49]. The six functional attributes of these peptides were defined as multilabel one‐hot encoding (activity against six drug‐resistant bacteria, toxicity to six types of organs) or multiclass labels (number of drug‐resistant bacteria inhibited, number of non‐drug‐resistant bacteria inhibited, and toxicity) for generation, regression, and classification tasks (Figure 2A,C). In practice, generating 200 000 new candidate AMPs (c_AMPs) and further screening high‐priority AMPs using the trained M3‐CAD pipeline required approximately two hours, greatly accelerating the discovery of AMPs.

### 2.4 Generating AMPs That Satisfy the Given Properties Based on a Multimodal VAE

The centerpiece of M3‐CAD is a generative model based on a multimodal, condition‐controlled variational autoencoder (m2cVAE) designed to generate novel peptides meeting specified attributes (Figure 2A). Unlike conventional VAEs, the m2cVAE incorporates separate encoders and decoders for sequence and 3D structure modalities. For sequence features, peptides up to 50 residues long were encoded into a 20 × 50‐dimensional tensor, where 20 represents the one‐hot encoding of amino acids and 50 corresponds to peptide length. Feature encoding and decoding were performed using a Gated Recurrent Unit (GRU). The 3D structural features employed a 3D voxel coloring method and a 3D Res‐Conv Net [50] for encoding and decoding.

To integrate the sequence and structural information with functional attribute data, four functional attributes, including the ability to inhibit DR bacteria, alpha‐helix propensity greater than 0.3, stability below 40, and charge greater than 1, were represented as a 1 × 4‐dimensional tensor. These attributes were feature‐extracted using a multilayer perceptron (MLP) and compressed into a 1 × 1 tensor. This tensor was multiplied by sequence and structural features before being input into the encoder and concatenated with latent features between the encoder and decoder. This architecture allows the m2cVAE to learn the relationships between peptide sequences, structural features, and controlled conditions.

During the inference phase (Figure 2A), two options are available: (1) without a template (untemplated): the GRU decoder randomly samples peptides that meet the specified conditions from the latent‐space probability distribution by inputting the corresponding conditions; and (2) with a template (templated): the GRU decoder samples specific peptides that meet the conditions from the latent‐space probability distribution by inputting the corresponding conditions. Notably, for the generation inference phase, only the sequence part is needed for generation; therefore, this part is quite efficient (3 min for 200 000 candidates). These peptides potentially satisfy various antimicrobial mechanisms and broad‐spectrum inhibitory conditions against MDROs, thereby expanding the combinatorial molecular space for AMP discovery.

To further verify sequence diversity, we quantified how “templated” versus “untemplated” generation strategies affected sequence diversity and length bias through (1) low‐dimensional embedding of all training and generated peptides via UMAP (Figure S2), (2) computing inter‐ and intra‐sequence similarity distributions (Figure S3), and (3) comparing length histograms before and after top‐300 activity filtering (Figure S4). The key results are discussed below.

Templated peptides form tight, high‐density clusters in UMAP embedding, whereas untemplated peptides are broadly distributed and overlap both positive and negative training sets, indicating a broader exploration of the sequence space. Both templated and untemplated candidates maintain < 50% maximum identity to any training sequence (preserving novelty), but templated peptides show very high internal similarity (80%–100%), consistent with local motif perturbations; in contrast, untemplated peptides show moderate internal similarity (30%–50%), reflecting greater diversity. The untemplated set spans 10–24 residues and retains this breadth among the top 300 candidates, whereas templated peptides concentrate at 14–18 residues to balance synthesis cost, stability, and membrane‐penetration efficiency. This length convergence further drives high sequence similarity in the templated pool.

To benchmark our 2D baseline (seqVAE) and demonstrate the benefits of incorporating 3D structural characteristics in designing more potent AMPs, we evaluated six AMP generators: five sequence‐only methods (PepDiffusion [51], AMPGAN v2 [52], AMP‐Designer [53], CLaSS [54], seqVAE) and our sequence + structure integrated m2cVAE—by generating 1000 random peptide sequences per method and predicting their activity against antimicrobial‐resistant strains with an external classifier [55]. SeqVAE exhibited a significantly higher inhibition rate than that of the AMP‐Designer and CLaSS, whereas m2cVAE consistently and significantly surpassed all sequence‐only models. These results demonstrate that the enhanced predicted antimicrobial activity arises from the effective incorporation of 3D structural features (Figure S5).

### 2.5 Ranking the Generated Peptides Using an MLP Regression Model

Considering the time‐intensive nature of predicting the 3D structures of numerous peptides, we incorporated a sequence‐based MLP as the second component of M3‐CAD for the initial screening of generated sequences (Figure 2B). The MLP is trained to rank peptides based on their ability to broadly inhibit drug‐resistant bacteria, and a higher priority is given to broad‐spectrum inhibitory peptides. The label for this regression task is the number of bacterial species that the peptide can kill. In practice, this stage of M3‐CAD selects 3000 high‐priority c_AMPs for 3D prediction using AlphaFold2, where the better candidates with a higher regression value progress to the final stage of the pipeline.

### 2.6 Identifying the Attributes of AMPs Using a Multilabel Classification Model Trained Using the Rebalancing Loss Function

The final component of M3‐CAD is a multimodal, multitask, and multilabel classifier designed to identify AMPs with diverse antimicrobial mechanisms, broad‐spectrum inhibition against drug‐resistant bacteria, and low toxicity. This classifier employs an MLP and a 3D Res‐Conv Net for feature extraction from peptide sequences and 3D structures in parallel with the previously mentioned m2cVAE. The extracted features were fused and dispatched to the three classification heads for multilabel classification (Figure 2C). We then assessed the performance of multilabel classifiers for predicting antimicrobial activity, mechanism, and toxicity using fivefold cross‐validation (Figure 2D). Additionally, we evaluated the antimicrobial activity prediction on an independent external test set [56], which showed that multimodal models outperform those relying solely on sequence or 3D structural data (Table S7).

During classifier training, severe label imbalances were found across the three multilabel classification tasks (Figure S1B–E), which could hamper the performance of the deep neural network model [57]. Therefore, we proposed a multilabel rebalancing loss function by extending the “Softmax + Cross Entropy” scheme to multilabel classification scenarios with multiple target classes. This ensured that the score of each target class was not lower than that of the non‐target class, thereby providing a balanced loss function to handle the imbalanced data distribution [58]. Ablation studies showed that the rebalancing learning strategy improved performance across all three multilabel classification tasks (Figure 2E).

For comparison between single‐task and multitask (multilabel in our setting), we implemented single‐task variants sharing the same encoder architecture but trained independently on one binary classification task at a time. We then compared their performance against the complete multitask M3CAD model under identical fivefold cross‐validation splits and hyperparameter settings. As summarized in Table S8, the multitask learner outperformed all single‐task baselines across every metric, with especially pronounced gains on data‐scarce targets such as *E. faecalis* and *A. baumannii*.

In this application, 3000 c‐AMPs were ranked based on predicted antimicrobial activity and toxicity confidence levels from the multilabel classifier. The 10 top‐ranked peptides were advanced as the final output of the M3‐CAD pipeline for wet lab experiments.

### 2.7 In Vitro Antimicrobial Evaluation of the Top AMPs Designed by M3‐CAD

**FIGURE 3:**
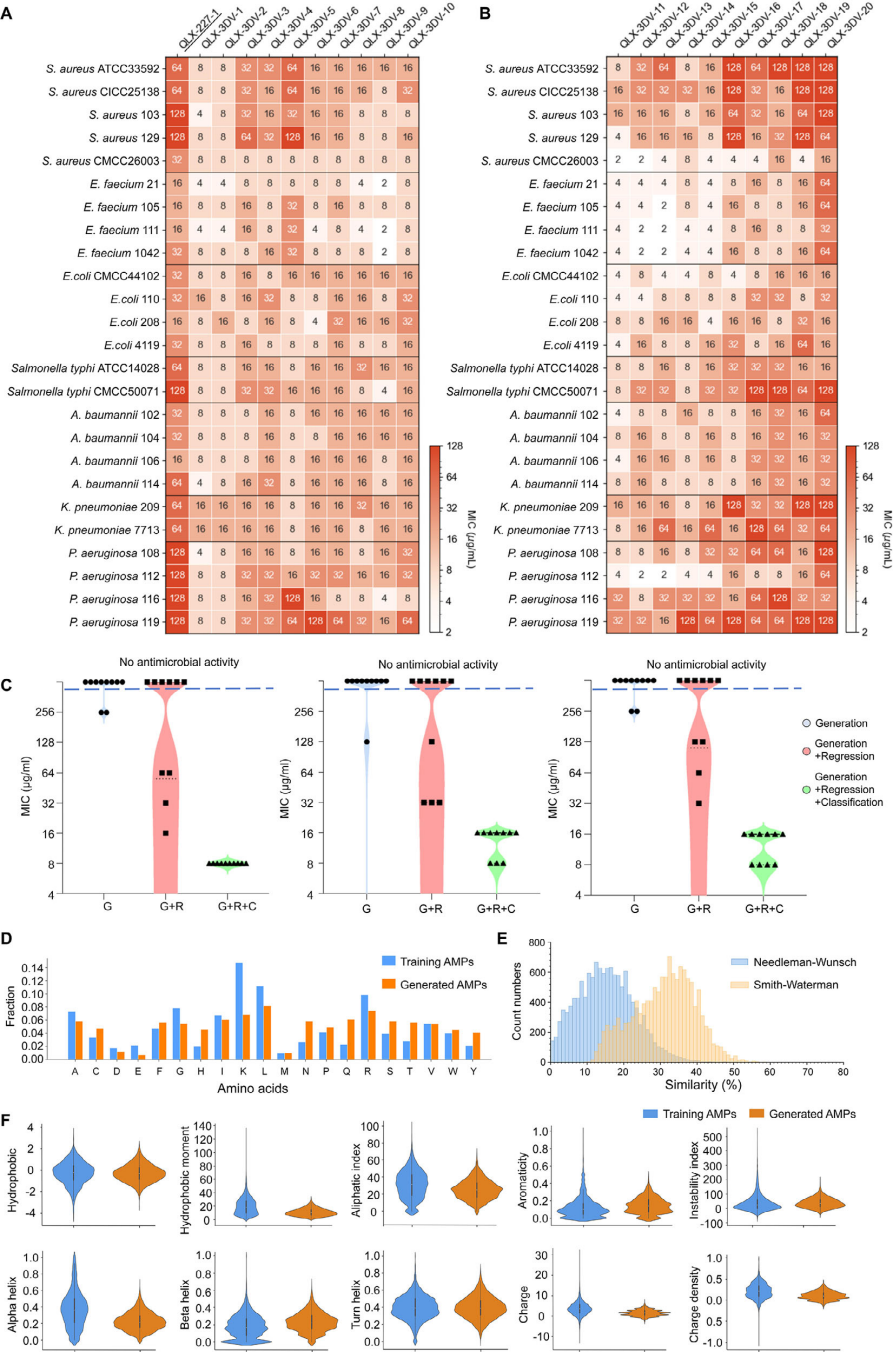
Wet‐lab validation of the antimicrobial activity of AMPs discovered by the M3‐CAD pipeline and characterization of the physicochemical properties. (A) Antimicrobial activity of QLX‐227‐1 and templated‐based peptides QLX‐3DV‐1–10 against standard and clinical isolates of multidrug‐resistant bacteria, represented by MIC (µg/mL); each result had at least 3 biological replicates. (B) Antimicrobial activity of *de novo* peptides QLX‐3DV‐11–20 against standard and clinical isolates of multidrug‐resistant bacteria, represented by MIC (µg/mL); each result had at least 3 biological replicates. (C) Violin plots showing the distribution of the tested MIC values of the top‐10 peptides from the complete and partial component pipelines (n  =  3 biologically independent replicates) against *S. aureus* CMCC26003 (left), *E. coli* CMCC44102 (centre), and *A. baumanii* 106 (right). The dashed line represents MIC > 256 µg/mL. The dotted lines in each data group represent the first quartile, median, and third quartile. (D) Comparison of the amino acid composition between the top 1000 AMPs generated by M3‐CAD and the training AMPs in the QLAPD database (n = 9392). (E) Distribution of the highest similarities of the top 1000 AMPs generated by M3‐CAD to the training AMPs in the QLAPD database. (F) Comparison of the physicochemical properties of training AMPs in the QLAPD database (n = 9392) and newly generated AMPs (n = 1000) using the M3‐CAD pipeline.

We used the template optimization function of M3‐CAD to optimize the scaffold QLX‐227‐1. Its minimum inhibitory concentration (MIC) values against gram‐positive and gram‐negative bacteria ranged from 16 to 128 µg/mL (Figure 3A), whereas the 25% hemolytic concentration (HC25) was 100.92 µg/mL (Figure S6). The 10 top‐ranked peptides output by the M3‐CAD pipeline in a single run were synthesized and tested for antimicrobial activity against 25 strains of ESKAPE MDROs including 19 clinical isolates of bacteria (Figure S7). QLX‐3DV‐1–10 inhibited at least five strains at a concentration of 8 µg/mL or lower (Figure 3A), performing better than the conventional AMPs in our training dataset and supporting the AMP design capabilities of M3‐CAD. The most efficacious AMPs, QLX‐3DV‐1 and QLX‐3DV‐2, demonstrated potent broad‐spectrum activity even at low concentrations, with MIC values of 4–8 µg/mL against gram‐positive strains and as low as 4 µg/mL against gram‐negative strains. To demonstrate the *de novo* peptide design capability of M3CAD, the 10 top‐ranked novel peptides were generated and experimentally validated against ESKAPE MDROs (Figure 3B). The untemplated peptides QLX‐3DV‐11 to 17 and 19 demonstrated antimicrobial potency with MIC values as low as 2 or 4 µg/mL. The growth‐inhibitory effects of QLX‐3DV‐1–18 exceeded those of LL‐37 and showed superior efficacy compared with that of SAAP‐148 [13], an optimized AMP derived from human LL‐37 through extensive wet lab experiments (Figure S8). Furthermore, QLX‐3DV‐1 and QLX‐3DV‐2 showed greater antimicrobial potency than that of four AMPs—C16G2 (NCT02594254), Omiganan (NCT00231153), OP‐145 (ISRCTN12149720), and DPK‐060 (NCT01447017)—all of which have progressed to clinical trials, further underscoring the therapeutic potential of QLX‐3DV‐1 and QLX‐3DV‐2 as next‐generation antimicrobial agents (Figure S8). QLX‐3DV‐1 and QLX‐3DV‐2 shared less than 45.7% sequence similarity with known AMPs, indicating their novelty (Table S9). Overall, these results confirm that M3CAD can generate functional antimicrobial peptides in both template‐based and entirely *de novo* manners.

### 2.8 Ablation Studies of M3‐CAD Using Wet Lab Experiments

To evaluate the necessity of each module in the M3‐CAD framework, the antimicrobial activity of the top 10 predicted peptides from different module combinations was assessed against *S. aureus*, *E. coli*, and *A. baumannii* (Figure 3C; Table S10). Peptides generated by the generative module alone (G) showed poor activity, with MIC ≥ 256 µg/mL against *S. aureus*, *E. coli*, and *A. baumannii*. Adding the regression module (G+R) improved efficacy, with 40% of peptides active against all three strains, 30% with MICs ≤ 32 µg/mL against *E. coli*, and the most effective peptide achieving a MIC of 16 µg/mL against *S. aureus*. The fully integrated framework (G+R+C) showed a further enhanced performance, identifying ten potent peptides (QLX‐3DV‐1–10) with the lowest MICs of 8 µg/mL. These results highlight the importance of combining the generative, regression, and classification modules to improve predictive accuracy and therapeutic potential.

To validate the importance of multimodal features in the M3‐CAD framework, peptides generated using only the sequence (baseline) were compared with those generated using multimodal features, including 3D structural features (proposed). As shown in Table S11, integrating 3D structural information enhanced antimicrobial efficacy. The baseline model produced peptides with MICs ranging from 8 to 256 µg/mL, indicating moderate to low activity, whereas the M3‐CAD pipeline consistently generated potent peptides (e.g., QLX‐3DV‐1–10) with MICs ≤ 16 µg/mL against all tested strains. These results underscore the critical role of 3D structural features in improving predictive accuracy and demonstrate the potential of M3‐CAD for designing highly effective antimicrobial peptides.

To ensure that integrating antimicrobial mechanism prediction into the M3‐CAD multitask classifier does not compromise antimicrobial activity or host toxicity, the top 10 peptides identified by the baseline classifier (focused on activity and toxicity) were compared with those from the proposed multitask classifier (integrating mechanism, antimicrobial activity, and toxicity predictions). As shown in Table S12, peptides designed with multiple antimicrobial mechanisms showed antimicrobial activity and hemolytic toxicity in wet lab experiments comparable with those from the baseline classifier. These findings confirm that the multitask classifier identifies AMPs with diverse mechanisms while maintaining potency and avoiding increased toxicity.

To validate the efficiency of our hierarchical screening strategy, we compared the full pipeline against a control setup in which the 1D screening step was removed, and applied the 3D scoring directly to all 200 000 generated sequences. As shown in Figure S9, skipping the 1D model improved the average scores for larger sets (top 500) but required 66 h of computation compared with 2 h for the full pipeline. Importantly, the selection of the critical top 20 candidates remained largely unaffected by the 1D filter. This confirms that the 1D model effectively accelerates the pipeline by removing low‐potential sequences prior to the expensive 3D modeling, without sacrificing the discovery of the most potent peptides.

### 2.9 Comparison of the Physicochemical Properties of c_AMPs Designed by M3‐CAD with Those of AMPs in the Training Set

We compared the sequence similarity and key molecular features (e.g., amino acid composition, charge count, hydrophobicity, and hydrophobic moment) of the top 1000 c_AMPs generated by the M3‐CAD pipeline in a single run. As shown in Figure 3D–F and Table S13, the newly designed AMPs are comparable to the training set AMPs in terms of physicochemical properties and most amino acid compositions. However, the newly designed AMPs show higher proportions of Lysine (K), Leucine (L), and Arginine (R) compared with those in the training set AMPs. Considering that Lysine, Leucine, and Arginine have been reported to correlate with the antimicrobial activity of AMPs [59], this difference may be attributed to the design of the M3‐CAD framework to generate and prioritize peptides with high antimicrobial activity.

Importantly, the sequence similarity between the newly designed AMPs and the training set AMPs was significantly different. More than 95% of the designed peptides had a maximum sequence similarity of less than 50% with any AMP in the training set (Figure 3E). This combination of retained physicochemical properties, changes in specific amino acid compositions, and notable differences in sequence similarity highlights the potential of M3‐CAD to generate novel peptides while maintaining or enhancing their antimicrobial activity.

### 2.10 Safety Assessment of the Leading AMPs Designed by M3‐CAD

**FIGURE 4:**
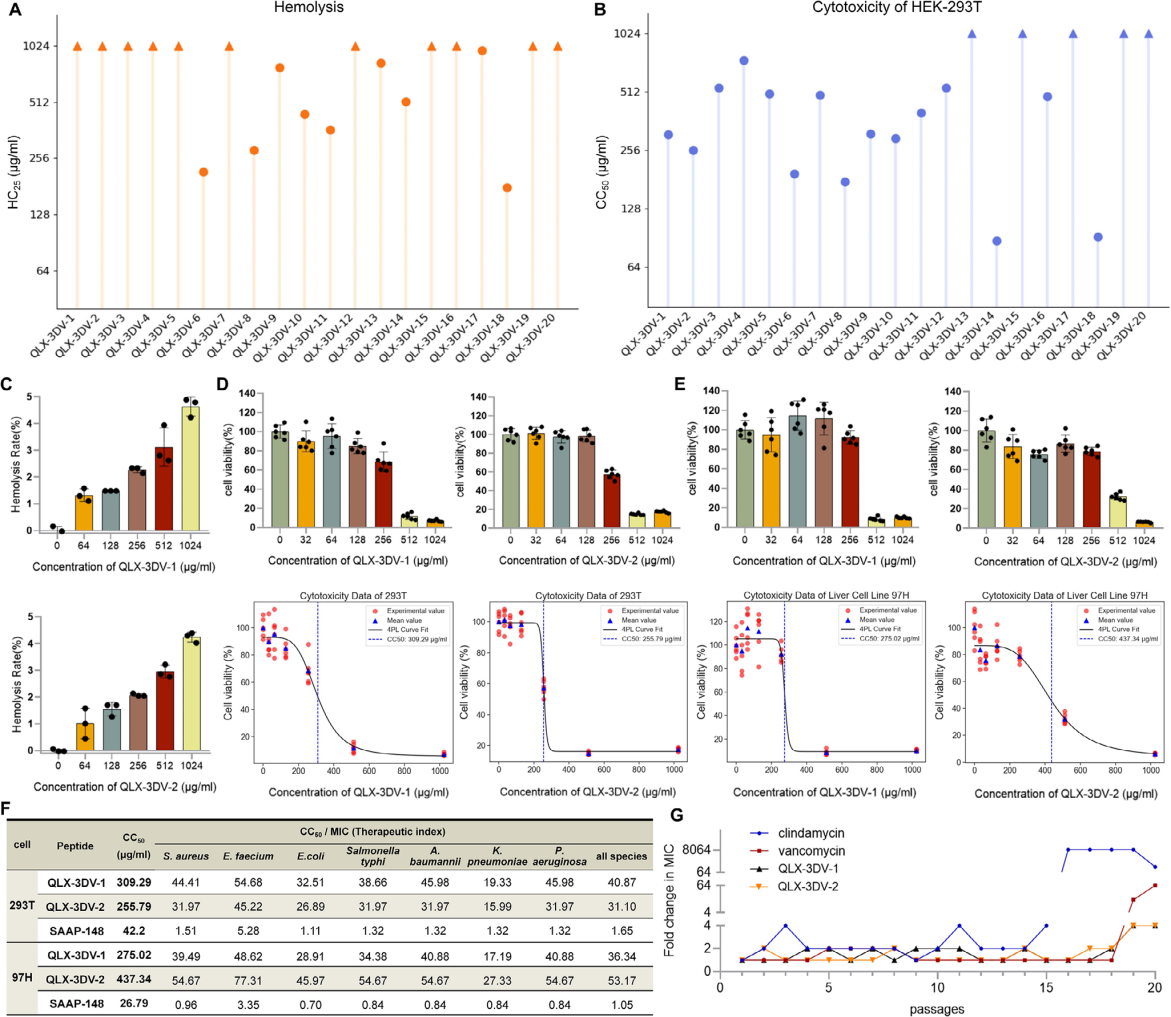
Off‐target toxicity and induced drug resistance of QLX‐3DV‐1 and QLX‐3DV‐2 in vitro. (A) Hemolysis (HC_25_) of QLX‐3DV‐1–20. HC_25_ values were calculated using linear interpolation. n  =  3 biologically independent replicates. (B) Cytotoxicity (CC_50_) of QLX‐3DV‐1–20 on HEK‐293T cell. CC_50_ values were calculated using four‐parameter logistic (4PL) curve function. n = 3 biologically independent replicates. (C) Hemolysis of QLX‐3DV‐1 and QLX‐3DV‐2 at different concentrations. n = 3 biologically independent replicates. (D, E) Cytotoxicity of QLX‐3DV‐1 and QLX‐3DV‐2 against HEK‐293T cells and MHCC97‐H at different concentrations. CC_50_ values were calculated using four‐parameter logistic (4PL) curve function. n = 6 biologically independent replicates. (F) Therapeutic index (CC_50_/geometric mean MIC) of QLX‐3DV‐1, QLX‐3DV‐2, and SAAP‐148 against various bacterial species. (G) Resistance‐acquisition studies of *S. aureus* CMCC26003 when cultured in the presence of sub‐MIC (1/2 × MIC) levels of clindamycin, vancomycin, and AMPs. n  =  3 biologically independent replicates. Data are presented as mean values ± SD. Data points show individual values, the central line represents the median, and the error bars indicate the range.

The off‐target toxicity of AMPs largely determines their potential for clinical application [16]. Hemolytic assays on human erythrocytes and cytotoxicity assays on the human embryonic kidney cell HEK‐293T and human hepatocellular carcinoma cell MHCC97‐H were performed to evaluate the safety of the leading AMPs designed by M3‐CAD. These results were compared with those of SAAP‐148 (Figure S10). For more than half of the peptides, the concentration inducing 25% hemolysis (HC_25_) exceeded 1024 µg/mL(Figure 4A; Figure S11). QLX‐3DV‐1 and QLX‐3DV‐2 caused no more than 5% hemolysis at 1024 µg/mL (Figure 4C). Meanwhile, the HC_25_ of SAAP‐148 was 26.89 µg/mL (Figure S10A). We then tested the cytotoxic concentrations at which 50% of HEK‐293T(Figure 4B,D; Figure S12) and MHCC97‐H cells (Figures S13 and S14; Figure 4E) remained viable (CC_50_) with the leading AMPs designed by M3‐CAD. The therapeutic indices of QLX‐3DV‐1–20 and SAAP‐148 were determined for 7 ESPAKE bacterial species (Figure 4F; Tables S14 and S15). The results demonstrated that QLX‐3DV‐1–20 exhibited higher CC_50_ values and superior therapeutic indices compared to SAAP‐148 across all 7 ESPAKE bacterial species, highlighting their excellent safety profiles.

### 2.11 QLX‐3DV‐1 and QLX‐3DV‐2 Showed Reduced Resistance Development Compared With That Using the Antibiotics Clindamycin and Vancomycin

Subsequently, we assessed the ability of *S. aureus* to develop resistance to QLX‐3DV‐1 and QLX‐3DV‐2, using the classical antibiotics clindamycin and vancomycin as references. As shown in Figure 4G, *S. aureus* cultured at sub‐inhibitory concentrations did not exhibit significant resistance to QLX‐3DV‐1 and QLX‐3DV‐2 after 20 passages. In contrast, *S. aureus* developed resistance to clindamycin and vancomycin after 15 and 18 passages, respectively, with their susceptibility decreasing by factors of 8189 and 64, respectively, by the 20th passages. These results, combined with the data obtained from antibacterial testing against clinically derived MDROs, suggest that QLX‐3DV‐1 and QLX‐3DV‐2 hold promise in addressing the rapidly emerging problem of AMR.

Combinatorial therapies of AMP with antibiotics offer a strategy to overcome multidrug resistance, potentiate antibiotic efficacy, and minimize cytotoxicity through dosage reduction. Consequently, we evaluated the synergistic effects of QLX‐3DV‐1 and QLX‐3DV‐2 with the fourth‐generation cephalosporin cefepime, which effectively targets gram‐negative organisms, using a modified checkerboard assay. Notably, the combination of cefepime with QLX‐3DV‐1 and QLX‐3DV‐2 demonstrated fractional inhibitory concentration indices (FICI) of 0.3125 and 0.1875, respectively, against *K. pneumoniae* (Table S16), indicating a strong synergistic interaction between these agents.

### 2.12 QLX‐3DV‐1 and QLX‐3DV‐2 Possess Multiple Antimicrobial Mechanisms

**FIGURE 5:**
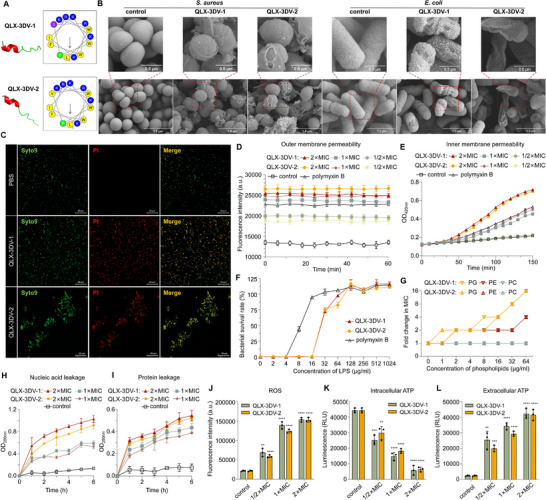
Bacterial membrane‐acting assessment of QLX‐3DV‐1 and QLX‐3DV‐2. (A) The helical wheel and AlphaFold2 predicted‐3D structure diagrams of QLX‐3DV‐1 and QLX‐3DV‐2 are shown, illustrating the α‐helical structure of the peptide. Helical wheels were generated using the HeliQuest tool available at https://heliquest.ipmc.cnrs.fr/index.html. (B) Scanning electron micrographs of *S. aureus* ATCC33592 and *E. coli* CMCC44102 treated with 1 × MIC of QLX‐3DV‐1 and QLX‐3DV‐2, with PBS as the control. Scale bar: 0.5.5 or 1.5 µm. (C) Confocal fluorescence microscopy of *S. aureus* ATCC33592 treated with 1 × MIC of QLX‐3DV‐1 and QLX‐3DV‐2, with PBS as the control. SYTO 9/PI was used to confirm membrane disruption. SYTO 9 and PI are green and red fluorescent dyes, which mark cells with intact bacterial membranes and those with membrane pore formation, respectively. Scale bar: 20 µm. (D) Outer membrane permeation effect in *E. coli* CMCC 44102 treated with QLX‐3DV‐1 and QLX‐3DV‐2 (1/2×, 1×, and 2×MIC) determined using NPN assay. n = 3 independent replicates. (E) Cytoplasmic membrane permeability in *E. coli* CMCC 44102 treated with QLX‐3DV‐1 and QLX‐3DV‐2 (1/2×, 1×, and 2× MIC) was determined using the ONPG assay. n = 3 independent replicates. (F) LPS competitive inhibition of QLX‐3DV‐1 and QLX‐3DV‐2 against *E. coli* CMCC 44102, n = 3 independent replicates. (G) Fold MICs of QLX‐3DV‐1 and QLX‐3DV‐2 against *E. coli* CMCC 44102 in the presence of phospholipids (PG, PE, PC). (H, I) Nucleic acid and protein leakage from *E. coli* CMCC 44102 treated with QLX‐3DV‐1 and QLX‐3DV‐2 (1× and 2× MIC), n = 3 independent replicates. (J) ROS generation in *E. coli* CMCC 44102 treated with QLX‐3DV‐1 and QLX‐3DV‐2 (1/2×, 1×, and 2× MIC), n = 3 independent replicates, n = 3 independent replicates. (K, L) Intracellular (K) and extracellular (L) ATP levels of *E. coli* CMCC 44102 treated with QLX‐3DV‐1 and QLX‐3DV‐2 (at 1/2×, 1×, and 2×MIC), n = 3 independent replicates. Data are presented as mean values ± SD. Data points show individual values, the central line represents the median, and the error bars indicate the range. *p* values were determined using one‐way ANOVA with Dunnett's multiple comparison test. *p < 0.05, **p < 0.01, ****p < 0.001, ****p < 0.0001.

The mechanism by which most cationic amphipathic AMPs inhibit bacteria typically involves electrostatic interactions with bacterial membranes, leading to pore formation and leakage of cell contents [11, 12]. The AlphaFold2‐predicted alpha‐helical structures of QLX‐3DV‐1 and QLX‐3DV‐2, combined with their model‐calculated cationic amphiphilicity, indicated a potential for membrane permeabilization (Figure 5A; Table S9). The helical wheel analysis revealed a distinct amphiphilic segregation of hydrophilic and hydrophobic regions in QLX‐3DV‐1 and QLX‐3DV‐2. This pattern was consistent with the canonical membrane‐permeabilization mechanism of antimicrobial peptides, highlighting their potential as promising antimicrobial agents. Scanning electron microscopy demonstrated that QLX‐3DV‐1 and QLX‐3DV‐2 induced membrane rupture and caused surface roughening and cracking in *S. aureus* and *E. coli* at 1× MIC, highlighting their potent membrane‐disruptive activity (Figure 5B). This mechanism was further corroborated by the SYTO 9/propidium iodide (PI) Live/Dead assay followed by confocal fluorescence microscopy analysis. In *S. aureus* treated with QLX‐3DV‐1 or QLX‐3DV‐2 at 1× MIC, significant red fluorescence was observed, indicating compromised membrane integrity and pore formation. In contrast, the vehicle control group showed exclusively green fluorescence, characteristic of that in intact viable cells (Figure 5C).

We evaluated outer membrane permeability using the hydrophobic fluorescent probe N‐phenyl‐1naphthylamine (NPN), which typically exhibits weak fluorescence in a hydrophilic extracellular environment but displays significant fluorescence upon interaction with the hydrophobic environment within bacterial intracellular compartments. Treatment with QLX‐3DV‐1 or QLX‐3DV‐2 increased the outer membrane permeability of *E.coli*, as evidenced by elevated NPN fluorescence compared with that upon PBS treatment, aligning with observations from polymyxin B (Figure 5D). Additionally, the inner membrane permeability was examined using o‐nitrophenyl‐β‐D‐galactopyranoside (ONPG), which can only enter the cytoplasm to be hydrolyzed and detected when the cytomembrane integrity is compromised. The results indicated that QLX‐3DV‐1 or QLX‐3DV‐2 increased the inner membrane permeability of *E. coli*, reflected by the increase in absorbance (Figure 5E).

We then considered whether QLX‐3DV and QLX‐3DV‐2 could target certain bacterial membrane components. We initially examined the interaction between the peptide and lipopolysaccharide (LPS), the main component of the bacterial outer membrane (Figure 5F). The antimicrobial activity of QLX‐3DV‐1 and QLX‐3DV‐2 against *E. coli* demonstrated dose‐dependent inhibition as the LPS concentration increased to 32 µg/mL. Furthermore, isothermal titration calorimetry (ITC) revealed high affinity between QLX‐3DV‐1 and QLX‐3DV‐2 and LPS (Figure S15). Subsequently, the antibacterial activity of QLX‐3DV was determined in the presence of exogenous phosphatidylethanolamine (PE) and phosphatidylglycerol (PG) (the main inner membrane phospholipids in bacteria), as well as against phosphatidylcholine (PC), a major phospholipid in the mammalian cell membrane (Figure 5G). The addition of PG and PE drastically weakened the antibacterial activity of QLX‐3DV and QLX‐3DV‐2 against *E. coli* in a dose‐dependent manner, suggesting preferential interaction with anionic phospholipids. Microscale thermophoresis (MST) revealed a high binding affinity of QLX‐3DV‐1 and QLX‐3DV‐2 with PG and PE (Figure S16). However, even at the highest concentration tested, the peptide showed no detectable interaction with PC (Figure 5G). This absence of binding underscores its superior antimicrobial selectivity. Overall, these findings suggest that QLX‐3DV‐1 and QLX‐3DV‐2 disrupt membrane integrity by targeting bacterial membrane‐specific lipids. The compromised integrity of bacterial membranes may potentially result in the leakage of intracellular contents. As illustrated in Figure 5H,I, QLX‐3DV‐1 and QLX‐3DV‐2 caused a significant increase in the UV absorbance at 260 and 280 nm in *E. coli*, indicating the leakage of intracellular nucleic acids and proteins.

In addition to the membrane disruption mechanism, QLX‐3DV‐1 and QLX‐3DV‐2 promoted the generation of intracellular reactive oxygen species (ROS) in a dose‐dependent manner, thereby killing bacteria through oxidative damage (Figure 5J). Membrane damage considerably disrupts bacterial energy metabolism. To explore this, extracellular and intracellular ATP levels were quantified, as ATP is a critical energy signaling molecule. Treatment with QLX‐3DV‐1 or QLX‐3DV‐2 markedly reduced the intracellular ATP levels, accompanied by a corresponding increase in the extracellular ATP levels in *E.coli* (Figure 5K,L).

**FIGURE 6:**
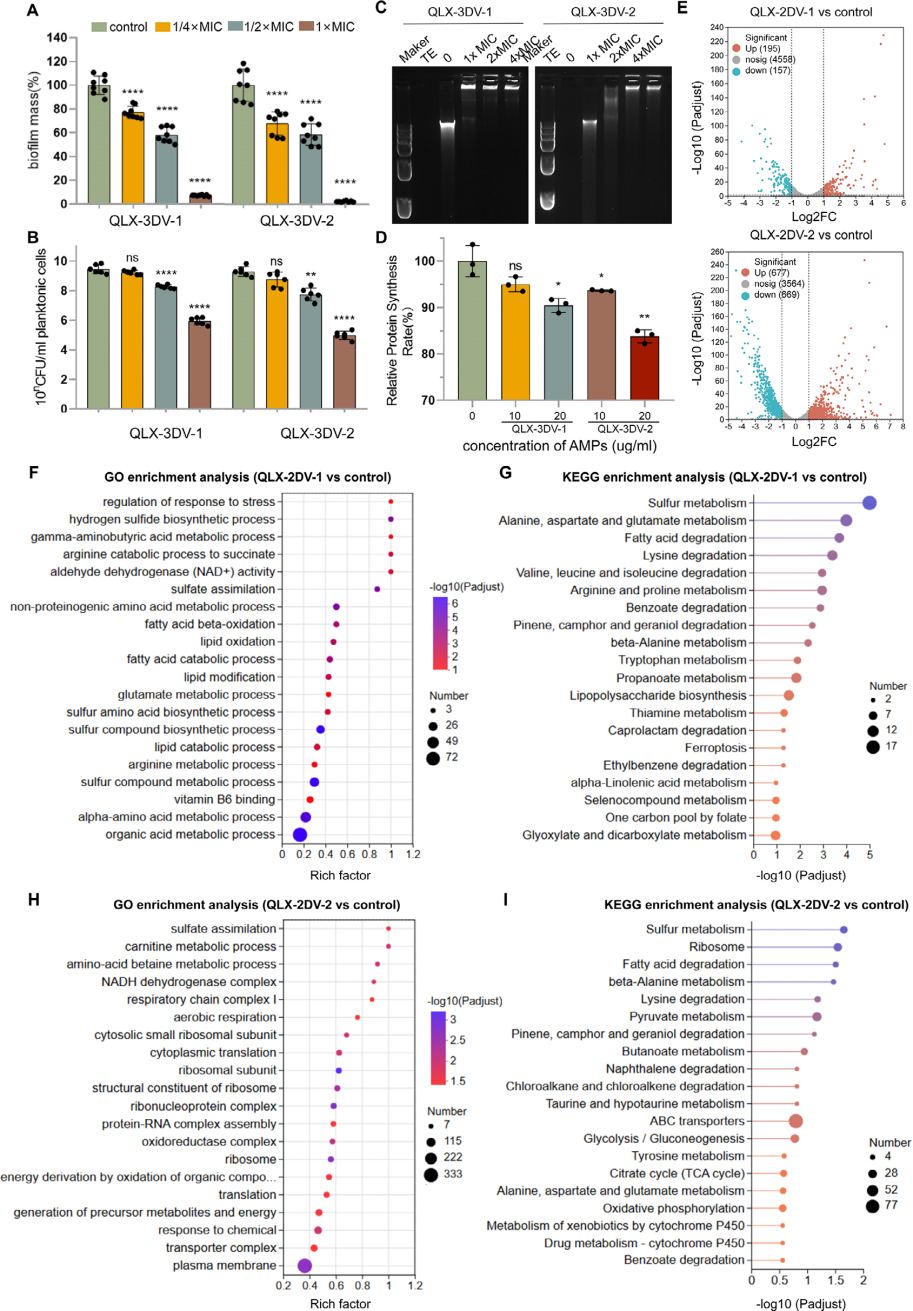
Transcriptomic sequencing to elucidate non‐membranolytic antimicrobial mechanisms. (A) Biofilm mass (%) of *S. aureus* ATCC33592 after treatment with QLX‐3DV‐1 and QLX‐3DV‐2 at different concentrations. n  =  8 independent replicates. (B) Concentration of planktonic cells of *S. aureus* ATCC33592 after treatment with QLX‐3DV‐1 and QLX‐3DV‐2 at different concentrations. n  =  6 biologically independent replicates. (C) DNA‐binding ability of QLX‐3DV‐1 and QLX‐3DV‐2 to *S. aureus* ATCC33592 genomic DNA. n = 3 independent replicates. (D) Relative protein synthesis rate of Cell‐free protein synthesis system treated with QLX‐3DV‐1 and QLX‐3DV‐2 at different concentrations. (E) Volcano plot of upregulated and downregulated genes (|Log2FC| ≥ 1, P adjust < 0.05) in *E. coli* CMCC 44102 after QLX‐3DV‐1 or QLX‐3DV‐2 treatment. (F) GO pathway enrichment analyses of differentially expressed genes in *E. coli* CMCC 44102 after QLX‐3DV‐1 treatment. (G) KEGG pathway enrichment analyses of differentially expressed genes in *E. coli* CMCC 44102 after QLX‐3DV‐2 treatment. (H) GO pathway enrichment analyses of differentially expressed genes in *E. coli* CMCC 44102 after QLX‐3DV‐1 treatment. (I) KEGG pathway enrichment analyses of differentially expressed genes in E. coli CMCC 44102 after QLX‐3DV‐2 treatment. Data are presented as mean values ± SD. Data points show individual values, the central line represents the median, and the error bars indicate the range. *p* values were determined using one‐way ANOVA with Dunnett's multiple comparison test. ns, not significant; *p < 0.05, **p < 0.01, **p < 0.001, ***p < 0.001.

Biofilms enhance bacterial resistance, anti‐phagocytic properties, and adherence and are considered a significant factor in the bacterial AMR emergence [60]. Inhibition of biofilm formation in *S. aureus* was assessed using crystal violet staining. As shown in Figure 6A, QLX‐3DV‐1 and QLX‐3DV‐2 inhibited *S. aureus* biofilm formation in a dose‐dependent manner. At 1/4× MIC, biofilm biomass was reduced without killing planktonic cells, indicating that QLX‐3DV‐1 and QLX‐3DV‐2 inhibit biofilm formation through specific mechanisms rather than by solely relying on bactericidal activity (Figure 6B).

Genomic DNA is a recognized intracellular target, and its interaction with antimicrobial peptides (AMPs) can interfere with gene expression, disrupt macromolecule synthesis, or compromise the essential components of cellular metabolism, ultimately contributing to their antimicrobial effects [11]. We assessed the DNA‐binding potential of QLX‐3DV‐1 and QLX‐3DV‐2 using a gel retardation assay. The results demonstrated that the peptide exhibited high binding affinity to the genomic DNA of *S. aureus* even at a concentration as low as 1× MIC (Figure 6C). The hindrance effect increased progressively with peptide concentration. This result indicates that QLX‐3DV‐1 and QLX‐3DV‐2 bind to *S. aureus* DNA, potentially disrupting its function and contributing to bacterial death, suggesting a possible bactericidal activity through interaction with bacterial DNA and interference with the transcription process.

In vitro transcription/translation assays were performed to evaluate any inhibitory activity exerted by QLX‐3DV‐1 and QLX‐3DV‐2 on one or both processes [61]. The inhibitory effect of the peptide was assessed by measuring luminescence intensity, which reflects luciferase activity from the luciferase generated via cell‐free protein synthesis. As shown in Figure 6D, QLX‐3DV‐1 and QLX‐3DV‐2 both inhibited protein synthesis at 20 µg/mL, with QLX‐3DV‐2 showing stronger activity by achieving significant inhibition at 10 µg/mL. The synergistic effect of membrane‐targeting and non‐membrane actions significantly elevated the genetic threshold for bacteria to escape through a single mechanism, thereby delaying the onset of drug resistance.

### 2.13 Transcriptome Analysis of *E.coli* Treated With QLX‐3DV‐1 or QLX‐3DV‐2

To elucidate the underlying molecular mechanism, we performed the RNA‐seq transcriptome sequencing on *E. coli* treated with QLX‐3DV‐1 or QLX‐3DV‐2. As shown in the volcano plots (Figure 6E), treatment with QLX‐2DV‐1 resulted in a total of 352 significant differentially expressed genes (DEGs), comprising 195 upregulated and 157 downregulated genes. In contrast, the QLX‐2DV‐2 treatment induced a more extensive transcriptional shift, with 1,346 significant DEGs, including 677 upregulated and 669 downregulated genes.

Gene Ontology (GO) enrichment revealed that QLX‐3DV‐1 significantly disrupted metabolic homeostasis in *E. coli*. The marked upregulation of these degradative processes suggests a potential disruption of central metabolism and energy homeostasis. Concurrent enrichment in lipopolysaccharide biosynthesis and stress response pathways further indicates a compensatory survival response to the peptide‐induced membrane perturbation and cellular stress (Figure 6F). Consistent with these findings, Kyoto Encyclopaedia of Genes and Genomes (KEGG) pathway analysis highlighted significant alterations in sulfur metabolism, fatty acid degradation, and amino acid metabolism (specifically alanine, aspartate, glutamate, and arginine) (Figure 6G). The enrichment of stress response regulation and lipopolysaccharide biosynthesis pathways indicates that QLX‐3DV‐1 induces a robust cellular stress response and potentially compromises cell envelope integrity. Differently, QLX‐3DV‐2 targets the cell membrane and protein synthesis machinery in *E. coli*. This is evidenced by the significant enrichment of GO terms related to the plasma membrane, transporter complexes, and transmembrane transport (Figure 6H). Simultaneously, strong upregulation of ribosome‐associated pathways and cytoplasmic translation indicates severe translational stress. KEGG analysis further supports the disruption of core functions, including sulfur metabolism, fatty acid degradation, and ribosomal pathways (Figure 6I). This comprehensive response suggests that the peptide induces membrane damage while concurrently inhibiting protein production, leading to a profound cellular crisis.

### 2.14 QLX‐3DV‐1 and QLX‐3DV‐2 Suppress *S. aureus* and *E. coli* in a Full‐Thickness Skin Wound Model without Toxicity

**FIGURE 7:**
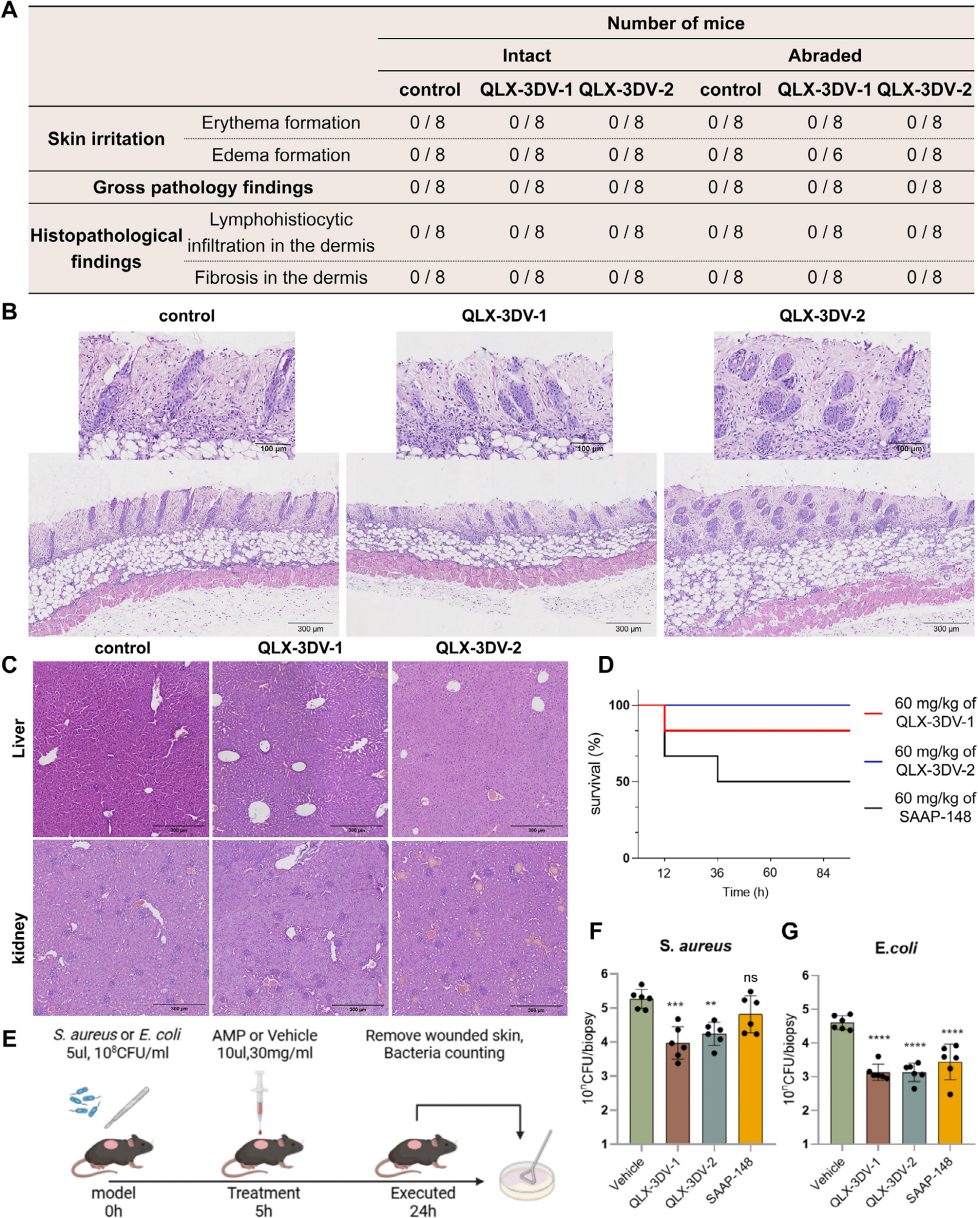
Safety and therapeutic efficacy of QLX‐3DV‐1 and QLX‐3DV‐2 in treating skin infections. (A) Mouse skin tolerance test. Mice with intact or abraded skin were treated with 5× the therapeutic dose (10 µL of 150 mg/mL) of QLX‐3DV‐1, QLX‐3DV‐2, or vehicle. Results are expressed as the number of animals out of the total number of animals in the group (n = 8) that showed signs of skin irritation or pathology within 72 h after treatment. (B) Representative H&E‐stained histopathological sections of tissue biopsies from abraded skin treated with QLX‐3DV‐1, QLX‐3DV‐2, or vehicle (n = 8 biologically independent replicates). (C) Representative H&E‐stained histopathological sections of the liver and kidney from mice intraperitoneally injected with QLX‐3DV‐1, QLX‐3DV‐2, or vehicle (n = 8 biological replicates). (D) Survival rate of mice intraperitoneally injected with 60 mg/kg QLX‐3DV‐1, QLX‐3DV‐2, or SAAP‐148 within 96 h of treatment (n = 8 biologically independent replicates). (E) Schematic representation of bacterial infection and AMP treatment experiments in a full‐thickness skin wound model. Created using BioRender.com. (F, G) The effect of therapeutic doses of QLX‐3DV‐1, QLX‐3DV‐2, SAAP‐148 (10 µL of 30 mg/mL), or vehicle on tissue bacterial burden in full‐thickness skin wound models infected with *S. aureus* (F, n = 6) or *E. coli* (G, n = 6). Data are presented as mean values ± SD. Data points show individual values, the central line represents the median, and the error bars indicate the range. *p*‐values were determined using two‐tailed Student's t‐test. ns, not significant; *p < 0.05, **p < 0.01, ****p < 0.001, ****p < 0.0001.

To further characterize the potential of QLX‐3DV‐1 and QLX‐3DV‐2 as antimicrobial agents, their potential adverse reactions upon local skin application in mice were evaluated (Figure S17A). Treatment of both shaved intact and abraded skin with 5x the therapeutic dose (10 µL of 150 mg/mL) of QLX‐3DV‐1 and QLX‐3DV‐2 showed no signs of skin irritation or pathology at 72 h post‐treatment (Figure 7A). No significant behavioral or body‐weight changes were observed, and no signs of systemic toxicity were found (Figure S17B). Histological examination revealed histopathological findings comparable with those of the control‐treated, QLX‐3DV‐1, and QLX‐3DV‐2 solution‐treated skin (Figure 7B; Figure S17C). To evaluate the systemic safety of QLX‐3DV peptides, mice were intraperitoneally injected with varying doses of QLX‐3DV‐1 and QLX‐3DV‐2. Pathological analysis revealed no significant hepatorenal toxicity at 40 mg/kg (Figure 7C). Survival curves over 96 h showed 100% survival with QLX‐3DV‐1 at 60 mg/kg, while QLX‐3DV‐2 showed an 83% survival rate, outperforming SAAP‐148 (50%) (Figure 7D).

Next, we evaluated the in vivo therapeutic effects of QLX‐3DV peptides in a full‐thickness skin wound model in mice locally infected with *S. aureus* or *E. coli*. As shown in Figure 7E, after removing the hair from the back of the mouse, full‐thickness wounds of 5 mm diameter were created on the dorsal skin of mice and infected with 5 µL of 10^8^ CFU/mL bacterial solution. After 5 h, the treatment group received 10 µL of 30 mg/mL of QLX‐3DV‐1 and QLX‐3DV‐2. Skin samples from the infected area were collected after 24 h for bacterial quantification. Compared with the vehicle, QLX‐3DV‐1 and QLX‐3DV‐2 reduced the *S. aureus* bacterial loads to 5.1% and 9.4%, respectively, and *E. coli* loads to 3.4% (Figure 7F,G). Both peptides demonstrated superior antibacterial activity compared with that of the control peptide SAAP‐148.

## 3 Discussion

Remarkable progress has been made in AI‐driven AMP discovery in recent years [18, 19, 20, 21, 22, 23, 24, 25, 26, 27, 28, 29, 30]. Regardless of the task (that is, identification, optimization, or generation) and the underlying network used, existing AI models inherently link the primary sequence of peptides and/or their sequence‐derived physicochemical properties to their bactericidal activity. Consequently, the information scope provided by the primary sequence inherently caps the potential of all methods utilizing these features for modeling. In this study, we sought to address a critical scientific query. Could there be undiscovered features and functional properties that could bolster the AI‐driven discovery of novel AMPs beyond conventional sequence features and antimicrobial activity properties? To our knowledge, this is the first study to systematically integrate 3D structural features and multi‐mechanism optimization into AI‐driven AMP discovery, addressing a fundamental limitation of conventional sequence‐based approaches.

By leveraging computational methods such as AlphaFold2 [32] and RoseTTAFold [33], we can now predict 3D protein structures with near‐experimental accuracy and atomic‐level precision. We postulated that integrating the 3D structural features of peptides could enhance the performance of AI in AMP design. Ablation studies supported our multimodal model that combined peptide primary sequence features with AlphaFold2‐predicted 3D structural features, and outperformed models relying solely on primary sequences or 3D structures in multilabel classification tasks concerning antimicrobial activity, inhibition mechanisms, and host toxicity of AMPs. Regarding the functional properties of AMPs, we hypothesized that AMPs featuring multiple antimicrobial mechanisms may exhibit enhanced bacterial inhibition efficacy, particularly against resistant superbugs. To investigate this, we reviewed the literature and manually annotated four known inhibitory mechanisms across 12 914 AMPs, which served as training data for the M3‐CAD pipeline and enabled us to design and screen AMPs embodying all four antimicrobial mechanisms. Moreover, we integrated variations in the inhibitory activities of AMPs against different bacterial types into the model, hypothesizing that this could facilitate broad‐spectrum AMP design. Wet lab evidence on QLX‐3DV‐1 and QLX‐3DV‐2 confirmed that the M3‐CAD pipeline, trained with the antimicrobial mechanism and inhibitory activity data of AMPs against six types of resistant bacteria, could generate multi‐mechanism AMPs that inhibited both gram‐positive and gram‐negative MDROs.

During the construction and training of the M3‐CAD pipeline, two critical technical queries were confronted: representation of polypeptide 3D structures with a long‐tail length distribution and imbalance of the functional labels of AMPs in multilabel classification tasks. For the first query, GNNs were proposed to represent the 3D structures of proteins predicted using AlphaFold2 [32]. The dimensions of the adjacency matrices of the GNN equaled the number of amino acids in the longest protein in the training set [62]. However, GNNs seem unsuitable for representing polypeptide 3D structures in AMP discovery because the lengths of AMPs available for training show a long‐tail distribution. With an increase in sequence length, the number of polypeptides continued to decrease, leading to an overly sparse GNN adjacency matrix. Moreover, owing to the sequential connection of amino acids within a polypeptide, relying on a single path for feature transformation may hinder feature transmission between distant amino acids. Therefore, we proposed a 3D voxel‐coloring method that transforms the problem into a 3D visual feature extraction task, addressed using a 3D‐CNN model demonstrating an improved performance compared with that of traditional GNNs for AMP discovery tasks. Notably, in the antimicrobial mechanism prediction task, the apparent contradiction between higher F1 but lower AUC for 3D‐CNN model versus GNNs arises from differences in score calibration and the type of signal each representation captures. The voxelized 3D representation presents each peptide as a dense spatial image, allowing the convolutional network to detect strong local 3D motifs such as amphipathic faces or clustered charged and aromatic residues [63, 64]. Consequently, the CNN often issues confident positive predictions for peptides with clear spatial signatures, which raises single‐threshold metrics such as F1 at the default operating point. However, because some true positives are signaled primarily by sequence connectivity rather than by an obvious 3D texture, the scores of the voxel model can be more bimodal and less well ordered across the entire population; this harms rank‐based, threshold‐free metrics such as the AUC. In contrast, GNNs operate on sparse sequence/adjacency graphs and tend to produce smoother, better calibrated probabilities that preserve global ranking and thus achieve a higher AUC. However, those moderate scores translate into fewer decisive positive calls at a fixed threshold, thus lowering the AP, F1, and accuracy. Regarding the second query, we introduced a multilabel rebalancing loss function for improved performance in all three multilabel tasks.

Although the integration of 3D structural features enhances the predictive performance of our model, it is important to acknowledge the limitations of utilizing AlphaFold2 for short, flexible antimicrobial peptides (AMPs). Because AlphaFold2 is primarily trained on globular proteins, the resulting voxel‐based features represent static, coarse‐grained approximations that may not fully capture the conformational ensembles or environment‐dependent structural transitions—such as those occurring in membrane‐bound states—that are critical to AMP activity. By contextualizing M3CAD within the broader landscape of recent machine learning advancements, including AMPSphere and other deep learning frameworks [65, 66, 67, 68, 69] for peptide screening, we position our approach as a multi‐modal strategy wherein 3D representations complement sequence and graph‐based data rather than serving as definitive structural models. Consequently, we emphasize that the high‐priority candidates identified by our pipeline should be considered with caution and experimentally validated using circular dichroism or NMR spectroscopy to confirm their functional conformations.

The speed and accuracy of M3‐CAD in AMP discovery are noteworthy. In a single run, it can generate and rank 200 000 peptides in approximately two hours. Wet lab validation confirmed the in vitro antimicrobial capabilities of all twenty high‐priority peptides. QLX‐3DV‐1 and QLX‐3DV‐2, the most promising candidate, exhibited multiple antimicrobial mechanisms, broad‐spectrum activity against MDROs, low host toxicity, and reduced resistance‐inducing propensity. With further modifications, such as N‐terminal acetylation or D‐amino acid substitution [16, 69, 70], M3‐CAD shows promise for advancing clinical trials, thereby highlighting its potential to expedite future AMP research and translational efforts. In future designs, we will proactively eliminate sequence features containing known unstable amino acids (such as cysteine) and systematically incorporate engineering modifications, such as C‐terminal amidation. This strategy aims to screen and optimize candidate AMPs that combine high stability with enhanced bioactivity. While the mechanistic labels for the broader dataset rely on model‐derived predictions, the specific multi‐mechanism activities of our lead candidates, QLX‐3DV‐1 and QLX‐3DV‐2, were rigorously validated experimentally, confirming the model's utility in identifying genuine multi‐targeting agents.

In summary, from the perspective of AI‐driven AMP design problems, we demonstrate that introducing polypeptide 3D structures significantly boosts the performance of multimodal models beyond models that solely utilize primary sequences or 3D structures. The proposed 3D voxel‐coloring method enhances the representation of polypeptide 3D structural features, further improving the downstream task performance. Including antimicrobial mechanism information and differential inhibitory activity data of AMPs against various bacteria as training data enables the AI to discover AMPs with multiple antimicrobial mechanisms and broad‐spectrum antimicrobial activity. These discoveries can foster future automated AMP design research with potential generalizable methodological implications for AI‐driven therapeutic peptide discovery.

## 4 Experimental Section

### 4.1 Collection and Annotation of Training Data

We compiled an AMP database, named QLAPD, which encompasses 12 914 AMP and 22 411 non‐AMP entries. Each AMP record included the amino acid sequence, 3D structure, and the following six functional attributes: (1) Inhibitory activity against six drug resistant bacteria, including *E. faecium*, *S. aureus*, *A. baumannii*, *P. aeruginosa*, and Enterobacter, and *Salmonella*; (2) Number of types of non‐resistant bacteria it can inhibit (ranging from 0 to 6); (3) Number of types of drug resistant bacteria it can inhibit (ranging from 0 to 6); (4) Four antimicrobial mechanisms: lipid bilayer disruption, cytoplasmic protein inhibition, DNA/RNA binding, and biofilm inhibition; (5) Toxicity; (6) Six kinds of organ toxicity: cardiotoxin, enterotoxin, neurotoxin, hemostasis impairing toxin, myotoxin, and others. Below, we provide details of the functional attributes and 3D structure features:

1. Details of AMP/non‐AMP annotation: The sequences of AMPs were primarily obtained from DBAASP [34], dbAMP [35], and DRAMP [36] repositories, with a few sourced from literature and patent databases such as PubMed and Google Scholar. We manually screened and binarized the inhibitory activity data of each AMP against six types of bacteria: *E. faecium*, *S. aureus*, *A. baumannii*, *P. aeruginosa*, Enterobacter, and Salmonella. Specifically, if an AMP has a MIC of ≤ 256 µg/mL against any resistant strain of a certain type of bacteria, the AMP is considered active against that resistant bacterial type, and vice versa. The same principle applies to non‐resistant strains. The non‐AMP instances with lengths of 10–50 amino acid residues were collected from the UniPort database [49], after discarding sequences that were annotated as AMP, membrane, toxic, secretory, defensive, antibiotic, anticancer, antiviral, and antifungal, and were used in this study as well.
2. Details of toxic/nontoxic annotation: Sequences with toxicity labels were curated from the DBAASP database [34]. Sequences with IC50/HC50 ≤ 256 µg/mL in DBAASP were considered as Toxic instances. Sequences of 10–50 amino acid residues in the UniProt database were defined as nontoxic after excluding sequences that have been defined as toxic in the DBAASP and UniProt databases. For other mechanism‐based toxicity, we use the label from the Uniprot database. For the organ‐based toxic, AMPs were annotated as organ‐toxic if toxicity was reported for at least one organ system.
3. Details of antimicrobial mechanism annotation: We annotated the lipid bilayer disruption, cytoplasmic protein inhibition, DNA/RNA binding, and biofilm inhibition mechanisms of the AMP by directly using the properties from the DBAASP database [34].
4. Details of peptide's 3D structure: The 3D structure of the peptide was predicted by the AlphaFold2 model [32] with the local install version of ColabFold [ColabFold: making protein folding accessible to all]. We use 8 A100 GPUs with 40 GB of memory for predicting peptide structure in the database. Concretely, AlphaFold2 produces five candidate models per sequence, each annotated with per‐residue pLDDT scores. We computed the mean pLDDT across all residues for each model and retained the structure with the highest average pLDDT. This “top‐ranked” model was then used to generate the 3D voxel grid, ensuring that downstream analyses rely on the most reliable structural prediction. We stored the predicted results in the format of the PDB [39], which records the type of each atom in the peptide, the coordinates of the atom in 3D space, and the type of amino acid residue to which the atom belongs.

To evaluate the peptide structure prediction methods, we have collected the wet‐lab valid protein structure dataset from the public, with the sequence length less than 50, and there are 79 PDB files in total. We have evaluated several methods, including Alphafold2 with MSA, Alphafold2 without MSA, ESMFold [40], and HelixFold [41]. The detailed evaluation results are shown in Table S1, where Alphafold2 with MSA outperforms all other baselines; thus, we decided to use Alphafold2 with MSA to predict all the protein structures in this work. TM‐score is a length‐normalized, topology‐focused metric more robust than RMSD for small proteins and peptides [72, 73]. It is well established that TM‐score > 0.5 typically indicates that the predicted model and the native structure share the same overall fold/topology. Therefore, an average TM‐score of 0.675 ± 0.141 for AlphaFold2 with MSA on our wet‐lab validated peptide set (79 PDBs, < 50 aa) places the predictions comfortably above the widely used “correct topology” threshold and near the high‐accuracy regime, supporting their practical utility for downstream modeling. For the model training process, the 3D voxel is shaped 3 × 64 × 64 × 64, and we use a CNN with residual connections to extract its features. We can set the batch size up to 128 for training; it uses about 12.8 GB of memory, available with our 4090D GPU with 24 GB memory.

All models were evaluated using the same stratified fivefold splits (generated once with a fixed random seed), so differences in performance reflect model behavior rather than sampling variability. To address severe class imbalance, we stratified by the multilabel annotation matrix and counted peptides with an explicit active/inactive annotation per organism in each partition: per training fold there were approximately 689 (*E. faecalis*), 4814 (*P. aeruginosa*), 4487 (*S. aureus*), 848 (*A. baumannii*), 3887 (Enterobacteriaceae), and 1427 (*Salmonella*) annotated peptides; the corresponding validation folds contained ∼101, 736, 675, 114, 643, and 236 annotated peptides, respectively. These counts reveal pronounced imbalance—particularly for *P. aeruginosa* and *S. aureus*—and motivated our use of a multilabel rebalancing loss (peptides lacking an assay for a given organism were omitted from that organism's loss calculation).

Similarly, for the multilabel toxicity classification task, the number of peptides annotated with each toxicity label (used in fivefold cross‐validation) are: 27 peptides annotated for Cardiotoxin, 17 for Enterotoxin, 900 for Neurotoxin, 137 for Hemostasis‐impairing toxin, 35 for Myotoxin, and 782 for Others. For the multilabel mechanism classification task (used in fivefold cross‐validation), the numbers of peptides annotated with each mechanism label are: 10 039 peptides annotated for Lipid Bilayer targeting, 239 for Protein targeting, 174 for DNA/RNA targeting, and 393 for Biofilm targeting.

### 4.2 The 3D Voxel Coloring Method

Based on 3D coordinates, the van der Waals radii of each atom, atomic mass, amino acid solubility, amino acid equipotential, and known information, we constructed a multi‐channel polypeptide structure coloring method. Specifically, this method uses the spatial structure of the atoms in the polypeptide sequence to determine the space occupied by the polypeptide. Using the center of gravity of the polypeptide as the reference point, the *x*‐axis, *y*‐axis, and *z*‐axis are constructed, with a unit distance of 1 angstrom on each axis. The method treats this unit distance as a voxel in a 3D grid. Only atoms within a range of L angstroms in the positive and negative directions of each coordinate axis are considered. The entire 3D voxel is represented as a cube with dimensions of L × 2 in length, width, and height, where L is any positive integer and is typically set as a multiple of 8 to facilitate subsequent feature extraction. The van der Waals radius of each atom, rounded to angstroms (with the rounding for each atom as follows: ‘H’: 1, ‘C’: 1.5, ‘N’: 1.5, ‘O’: 1.5, ‘S’: 2) [42], determines the space it occupies in the cube.

To leverage the inherent properties of atoms and various amino acids, the method innovatively presents a multi‐channel polypeptide coloring approach inspired by the RGB multi‐channel visual imaging concept. Specifically, the properties of the atom (atomic mass), the solubility of the amino acid it constitutes, and the acidity or basicity of the amino acid are treated as three distinct channels for coloring the peptides. In the first channel, atomic mass is rounded and used directly as the voxel value. In the second and third channels, related to amino acid properties, the spatial positions of multiple atoms can represent the position of a single amino acid. Therefore, for the second solubility channel, the solubility of different amino acids is categorized into hydrophobic and hydrophilic amino acids. To differentiate these two types, and with a background voxel value of 0, the method assigns a value of 128 to hydrophobic amino acids and a value of 255 to each atom of hydrophilic amino acids. Similarly, for the third pH channel, the voxel values for acidic, neutral, and basic amino acids are assigned as 86, 168, and 255, respectively.

### 4.3 Multimodal Conditional Variational Auto‐Encoder for Peptide Generation

The variational auto‐encoder has been a powerful tool to generate the antimicrobial peptide recently. It is mainly based on learning the meaningful latent data embedding from the training data with a reconstruction loss and a regularization loss. The training process on sampling the data could be formulated as:

(1) $$p\left(z|x\right)=\frac{p\left(z\right)p_{\theta }\left(x|z\right)}{\int dzp\left(z\right)p_{\theta }\left(x|z\right)}$$

To enable the conditional generation of the target data, a conditional VAE was proposed with an additional category supervision loss term, which enables the feature embedding to be directly applied to other situations. This process could be formulated as follows:

(2) $$L_{\mathrm{VAE}}\left(\theta ,\varphi \right)=E_{q\varphi \left(z|x\right)}\left[\log p\theta \left(x|z\right)\right]-D_{\mathrm{KL}}\left(q\phi \left(\left.z\right|x\right)∥p\left(z\right)\right)$$

However, all the above‐mentioned autoencoders only take the simplified scenarios into account. The ignorance of the structure could do harm to the learning process of peptide generation. Moreover, there is another question on how to embed the condition information into the training process of the multimodal cVAE. Thus, in this work, we propose to use a novel multimodal cVAE. Unlike the previous VAE that only takes the sequence as the input, our multimodal cVAE has two encoders and two decoders, which are used to encode/decode structure input and sequence input, respectively. In order to generate the AMPs with the expected attributes, we need to inject the conditional information (e.g., broad‐spectrum activity against drug‐resistant bacteria, non‐toxicity) into the autoencoder.

For the generation part, we use an SGD optimizer with a learning rate of 0.001. The batch size is set to 64, and models are trained for 80 epochs. The parser defaults to a weight decay of 0.0005.

### 4.4 Multilabel Multimodal Rebalancing Learning for Classification

To further identify the MDR AMPs from the generated sequences, we proposed an efficient multimodal multilabel model to take advantage of both sequence information and structure information. Specifically, we use a 3D‐ResNet *f*_struct_ to capture the structure information *x*_struct_ based on the above‐mentioned structure coloring features. To extract the sequence feature *x*_seq_ of AMPs, we use a multi‐layer neural network *f*_seq_ producing a fixed‐length embedding. These embeddings are concatenated to form a unified multimodal feature vector, which is then passed through a standalone multilayer perceptron to predict continuous antimicrobial activity scores in the classification branch for multilabel classification. The classification head *f*_head_ is a multi‐layer neural network that takes the fused feature as the input and the label number of the target task as the output. Let *y* be the prediction result in the form of a logistic function. This process is formulated as:

(3) $$y=f_{\mathrm{head}}\left(\left[f_{\mathrm{struct}}\left(x_{\mathrm{struct}}\right),f_{\mathrm{seq}}\left(x_{\mathrm{seq}}\right)\right]\right)$$

(4) $$L_{\mathrm{ce}}=-\log\frac{e^{\mathrm{Si}}}{\sum_{j=1}^{L}e^{\mathrm{Sj}}}=\log\left(1+\sum_{j=1,j\neq i}^{L}e^{\mathrm{Sj}-\mathrm{Si}}\right)$$

Considering that the imbalance in data distribution will hinder the learning process of the deep neural network model, we adopt a rebalancing loss to handle the imbalanced multilabel classification task. In imbalanced multi‐class classification, one can rebalance the dataset by augmenting samples of underrepresented classes. However, in a multilabel setting, increasing instances for a minority label may exacerbate the overall imbalance, since those added samples often also include one or more majority labels. The above‐mentioned phenomenon motivates us to propose a rebalancing loss for the unbalanced multilabel classification task. Specifically, we extend the “softmax + cross entropy” scheme to a multilabel classification scene with multiple target classes. The method is based on the consideration that “the score of each target class is not less than that of each non‐target class”. The balance loss function is obtained to handle the imbalanced data distribution.

(5) $$L_{\mathrm{zlpr}}=\log\left(1+<y,e^{-s}>\right)+\log\left(1+<1-y,e^{s}>\right)$$

For the identification part, we use the AdamW optimizer at a learning rate of 0.002. Here, the batch size is 16 and training runs for 50 epochs. Weight decay defaults to 0.0005. It is worth noting that three backbones with corresponding classification heads are used to conduct the multilabel classification task on activity, toxicity, and mechanism. The variational autoencoder employed for peptide generation is a fully independent model and does not share weights or parameters with either the regression or classification modules.

### 4.5 Method for Calculating the Physicochemical Properties and Sequence Similarity of the Peptides

Physicochemical properties such as hydrophobicity, hydrophobic moment, aliphatic index, aromaticity, instability index, alpha‐helix, beta‐sheet, turns, charge, isoelectric point, charge density, and flexibility were estimated using the ProteinAnalysis function of the BioPython library [74].

The similarity between sequences was calculated using the Needleman–Wunsch algorithm, a fundamental method for sequence alignment. This algorithm is implemented in the BioPython library [74], specifically through the pairwise2 module. A function named calculate_similarity was created to facilitate this task. It accepts two sequences in string format, seq_gen and seq_tem, and converts them into Seq objects, as defined in BioPython. The sequences are then globally aligned using the pairwise2.align.globalms method, which stands for “global alignment using the Needleman‐Wunsch algorithm with custom scoring”. The scoring system includes a match score of 1, a mismatch score of −1, a gap opening penalty of −0.5, and a gap extension penalty of −0.1. The function returns the score of the optimal alignment divided by the length of the first sequence (seq_gen). This normalized score provides a measure of similarity between the two sequences, taking into account differences in their lengths.

### 4.6 Data Augmentation

To enhance the robustness of the VAE and improve the quality of generated sequences, we incorporated a bespoke data augmentation mechanism designated as random_mask_and_shift during the training process. Inspired by Masked Language Modeling (MLM) in natural language processing, this strategy aims to optimize latent space representation learning via a denoising autoencoding approach. The amino‐acid masking and shifting augmentation method prevents overfitting to the training data (Figure S18).

Specifically, this function applies stochastic perturbations to input sequences prior to encoding. This process serves two primary objectives: first, it prevents overfitting by introducing random noise, compelling the model to capture the underlying biological grammar of AMPs rather than memorizing specific training instances (i.e., preventing identity mapping); second, it establishes a denoising task that challenges the decoder to reconstruct the original, unmasked sequence from corrupted input, thereby reinforcing the model's capacity to infer and generate biologically relevant information.

The intensity of this augmentation is governed by the hyperparameter α (masking ratio). To determine the optimal value for α, we conducted a systematic hyperparameter sensitivity analysis. We evaluated the model's generative performance across different α settings, using the count of valid AMP sequences produced during three independent unconditional sampling runs (500 sequences per run) as the metric. The empirical results demonstrated that the model achieved optimal performance in generating valid and coherent peptide sequences with α set to 0.1. Consequently, α = 0.1 was adopted as the default setting for the final model training.

### 4.7 Strains and Animals

Standard strains included *S. aureus* ATCC33592, *S. aureus* CICC25138, and *Salmonella enterica* ATCC14028, which were purchased from the China Center of Industrial Culture Collection (CICC). Standard strains including *E.coli* CMCC44102, *Salmonella enterica* CMCC50071, and *S. aureus* CMCC26003 were purchased from the National Center for Medical Culture Collections (CMCC). Clinical multidrug‐resistant strains, including *S. aureus* 103, *S. aureus* 129, *E. faecium* 21, *E. faecium* 105, *E. faecium* 111, *E. faecium* 1042, *E.coli* 110, *E.coli* 208, *E.coli* 4119, *A. baumannii* 102, *A. baumannii* 104, *A. baumannii* 106, *A. baumannii* 114, *K. pneumoniae* 209, *K. pneumoniae* 7713, *P. aeruginosa* 108, *P. aeruginosa* 112, *P. aeruginosa* 116, and *P. aeruginosa* 119, were obtained from the clinical Laboratory of Qilu Hospital. All experiments using the above strains were carried out in a BSL‐2 laboratory. The clinical laboratory of Qilu Hospital detected bacterial resistance to various antibiotics. All strains were streaked on Nutrient Broth (NB) agar medium and incubated overnight at 37°C.

Female C57BL/6J mice were purchased from Beijing Vital River Laboratory Animal Technology Co., Ltd. They were cultured in sterile isolators at the BSL‐2 Laboratory Animal Center of Shandong University on a 12 h–12 h light‐dark cycle with an ambient temperature of 20°C–26°C, humidity of 40%–70%, and ad libitum access to food and water. All animal experiments involved were approved by the Animal Care and Animal Experiments Committee of Qilu Hospital of Shandong University (DWLL‐2023‐104). For each experiment, mice were randomized into experimental groups. The investigators were blinded to the group allocation during the experiment and the outcome processing.

### 4.8 Synthesis, Purification, and Identification of Peptides

Peptides were synthesized by Jiangsu Genscript Biotech Co., Ltd. and Nanjing Top‐Peptide Biotechnology Co. Ltd., using Solid Phase Peptide Synthesis. Peptides were purified using high‐performance liquid chromatography; the final purities of the peptides were >95%, and their identity was confirmed using Mass Spectrometry (results shown in Figures S19–S25). The sequences of the peptides are shown in Tables S17–S20.

### 4.9 Antimicrobial Activity Assays

The antimicrobial activity of the peptides was examined in sterile 96‐well plates using the broth microdilution according to the CLSI standard. The colonies were suspended in saline solution, the turbidity was adjusted to McFarland 0.5 to reach a bacterial concentration of 10^8^ CFU/mL, and again diluted 100 times for the inhibition test. Then, 50 µL of bacterial suspension at 1 × 10^6^ CFU/mL was incubated with the same volume of different concentrations of peptide solution [serial twofold dilutions in Mueller–Hinton Broth (Hopebio)]. After 18–20 h of incubation at 37°C, the MIC was defined as the minimum drug concentration that completely inhibited visible bacterial growth. All experiments were performed with three independent replicates.

### 4.10 Checkerboard Assay

A checkerboard assay was used to determine the synergistic effect of AMP and cefepime against *Klebsiella pneumoniae*. The initial concentrations of the AMP and cefepime were 32 and 128 µg/mL, respectively. Twofold serial dilutions of the AMP were prepared in vertical rows, and twofold serial dilutions of cefepime were prepared in horizontal rows using a 96‐well plate. The plates were prepared well to obtain a single plate in which both the antimicrobial agents were cross‐diluted. *Klebsiella pneumoniae* strains were cultured in the exponential phase in an MH broth medium. The final inoculum used was 5 × 10^5^ CFU/mL. The plate was incubated at 37°C for 18 h. Fractional inhibitory concentration index (FICI) values were defined as the lowest concentration at which a combination of the AMP and cefepime inhibited bacterial growth. The FICI was calculated using the following formula:

$$\begin{array}{ccc} & & \mathrm{Fici}=[\mathrm{MICAMP}\;\mathrm{in}\;\mathrm{the}\;\mathrm{presence}\;\mathrm{of}\;\mathrm{cefepime}]\;/\;\mathrm{MicAMP}+\\ & & \;[\mathrm{MICcefepime}\;\mathrm{in}\;\mathrm{the}\;\mathrm{presence}\;\mathrm{of}\;\mathrm{AMP}]\;/\;\mathrm{Miccefepime} \end{array}$$

Synergy was defined as FICI ≤ 0.5, antagonism as FICI > 4.0, and no interaction when the FICI was between 0.5 and 4.0 [71].

### 4.11 In Vitro Analysis of Toxicity

Human embryonic kidney (HEK‐293T) cells and human hepatocarcinoma cells MHCC97H were obtained from the American Type Culture Collection (ATCC) and grown at 37°C in a humidified atmosphere containing 5%CO_2_ in Dulbecco's modified Eagle's medium (DMEM) supplemented with 1% antibiotics (penicillin and streptomycin) and 10% fetal bovine serum (FBS). The cytotoxicity of peptides against 293T and 97H cells was determined using the CCK8 assay. HEK‐293T and 97H cells were seeded on 96‐well microplates at a density of 8 × 10^3^ cells/well in 100 µL of culture medium and cultured at 37°C for 24 h. The culture medium was then removed and 100 µL culture medium with the peptide at serial twofold diluted concentrations was added to each well. Wells containing cells without peptides served as controls. The cells were cultured for another 24 h, followed by replacement of the media with 10% CCK‐8 containing fresh Dulbecco's Modified Eagle Medium (DMEM) and another 1 h incubation at 37°C. The absorbance of the solution was measured at 450 nm using a microplate reader. The cell viability was calculated according to the equation:

$$\begin{array}{ccc} & & \mathrm{Cell}\;\mathrm{Viability}\;(\%)=[\mathrm{OD}450\;\mathrm{nm}(\mathrm{peptide})-\mathrm{OD}450\;\mathrm{nm}(\mathrm{blank})]\\ & & \;/\;[\mathrm{OD}450\;\mathrm{nm}(\mathrm{negative})-\mathrm{OD}450\;\mathrm{nm}(\mathrm{blank})]\;\times \;100 \end{array}$$

Human red blood cells (hRBCs) were purchased from Xiamen Immocell Biotechnology Co., Ltd. For the hemolysis test, fresh human red blood cells (hRBCs) were washed three times with PBS (35 mm phosphate buffer, 0.15 m NaCl, pH 7.4) by centrifugation for 5 min at 1000 × *g* and resuspended in PBS. Then, 50 µL peptide solutions (serial twofold dilutions in PBS) were added to 100 µL of hRBC suspension [8% (v/v) in final] in PBS and incubated for 1 h at 37°C. Samples were centrifuged at 1000 × *g* for 5 min, and hemoglobin release was monitored by measuring the supernatant absorbance at 570 nm in a flat‐bottom 96‐well plate (Servicebio). hRBCs in PBS or 0.1% Triton X‐100 were used as the negative and positive controls, respectively. The hemolysis percentage was calculated according to the equation:

$$\begin{array}{ccc} & & \mathrm{Hemolysis}\;(\%)=[\mathrm{OD}570\;\mathrm{nm}(\mathrm{peptide})-\mathrm{OD}570\;\mathrm{nm}\;(\mathrm{PBS})]\\ & & \;/\;[\mathrm{OD}570\mathrm{nm}\;(0.1\%\;\mathrm{Triton}\;X\;100)-\mathrm{OD}570\mathrm{nm}\;(\mathrm{PBS})]\;\times \;100 \end{array}$$

### 4.12 Drug Resistance Assay

For comparison, the development of resistance to the clinically relevant antibiotics clindamycin (Macklin) and vancomycin (Macklin) was determined. The evolution of resistance by *S. aureus* CMCC26003 to antibiotics and the peptide was monitored for 20 days of serial passaging in liquid NB. Cells were passaged every 24 h. Briefly, 1 mL of bacterial suspension (1 × 10^6^ CFU/mL) was incubated with antibiotics or peptide solution at half the minimal inhibitory concentration at 37°C, 200 rpm. After each incubation period, a new inoculum of 10^6^ cells was prepared for inoculation of the following passage containing fresh medium and increased doses of the antimicrobial agent. During the 20 days of serial passaging, the minimum inhibitory concentration of antibiotics or peptides to each generation of cells was determined. The MIC determination method was as previously described. The development of bacterial resistance was defined as a greater than fourfold increase in MICs compared to the initial MICs.

### 4.13 Scanning Electron Microscopy (SEM)

*S. aureus* ATCC33592 and *E. coli* CMCC 44102, grown in the mid‐log phase, was incubated with 1× MIC of QLX‐3DV‐1 and QLX‐3DV‐2 at 37°C for 4 h. Then, the mixture was centrifuged at 5500 × *g* for 10 min, and the bacterial pellets were collected at the bottom of the centrifuge tube and washed with PBS four times. The samples with no treatment were used as the control samples. To the bacterial precipitate, glutaraldehyde (2.5%) was added and dried by gradient concentration of ethanol, then plated with gold at the critical point. Finally, the bacterial morphology was photographed using a Hitachi S‐4700 electron microscope.

### 4.14 Membrane Permeabilization Assay

The membrane permeability of the peptide was determined by using the LIVE/DEAD BacLight Bacterial Viability Kit (Invitrogen). *S. aureus* ATCC33592 was grown to mid‐log‐phase, centrifuged (5000 × *g* for 10 min), washed, and resuspended in PBS to 1 × 10^6^ CFU/mL. The peptide solution was added to 100 µL of this bacterial suspension so that the final concentration of peptide was equal to the minimum inhibitory concentration. Bacterial cells were incubated with peptides at room temperature for 1 h. Bacterial cells in PBS were used as the negative controls. Then, they were centrifuged at 5000 × *g* for 5 min and resuspended in 50 µL staining solution. Mix thoroughly and incubate at room temperature in the dark for 15 min. Trap 5 µL of the stained bacterial suspension between a slide and an 18 mm square coverslip. Observe in confocal microscopy (LSM980) equipped with specified filter sets.

### 4.15 Outer Membrane Permeability Assay

*E. coli* CMCC 44102 cultured to logarithmic growth phase was adjusted to OD_600nm_ = 0.1. After centrifugation (5000 g, 5 min), the pellet was washed three times with 5 mm HEPES buffer containing 5 mm glucose and resuspended. For the assay, 50 µL of AMP solutions at varying concentrations were mixed with 100 µL, bacterial suspension, followed by addition of 50 µL NPN (40 µm) under dark conditions. Fluorescence intensity was measured every 6 min for 60 min using a microplate reader (excitation λ = 350 nm, emission λ = 420 nm), with PBS serving as the negative control and polymyxin B as the positive control.

### 4.16 Inner Membrane Permeability Assay

*E. coli* CMCC 44102 was cultured in MHB medium supplemented with 2% lactose until the logarithmic growth phase was reached. The bacterial suspension was adjusted to OD_600nm_ = 0.1 and centrifuged at 5000 g for 5 min, then the pellet was washed three times with PBS and resuspended. Subsequently, 100 µL of the bacterial suspension was mixed with 50 µL of AMP solutions at varying concentrations and 50 µL of 6 mm ONPG solution. The absorbance at 420 nm was measured every 10 min for 150 min using a microplate reader, with PBS serving as the negative control and polymyxin B as the positive control.

### 4.17 Nucleic Acid and Protein Leakage Assay

*E. coli* CMCC 44102 in logarithmic phase (adjusted to OD_600nm_ = 0.1) was centrifuged (5000 g, 5 min) and washed three times with PBS. After 1:1 mixing with peptide solutions, the mixtures were incubated at 37°C. Samples (1 mL) were collected every 1 h for 6 h and centrifuged (12 000 rpm, 2 min). Supernatants were collected, and the absorbance at 260 and 280 nm was measured using a microplate reader, with PBS as the negative control.

### 4.18 LPS Competitive Inhibition Assay

Lipopolysaccharide (LPS) was dissolved in PBS with different concentrations, which were mixed with an equal volume of peptide to incubate at 37°C for 1 h. Subsequently, the mixture was incubated with an equal volume of bacterial suspension (1 × 10^6^ CFU/mL) for 18 h, with the peptide present at a concentration of 1 × MIC. The absorbance at 600 nm was measured by a microplate reader, and the bacterial survival rate was calculated to assess the binding ability of the peptide to LPS. Polymyxin B served as positive control.

### 4.19 ROS Generation Assay

*E. coli* CMCC 44102 in logarithmic phase was diluted to OD_600nm_ = 0.3 in PBS. The bacterial suspension was incubated with 10 µm 2’,7’‐Dichlorodihydrofluorescein diacetate (DCFH‐DA) at 37°C for 30 min, then washed by PBS for three times. Then, 190 µL of the mixture was incubated with 10 µL of AMPS at different concentrations for 15 min. The fluorescence (excitation wavelength = 488 nm, emission wavelength = 525 nm) was recorded by a microplate reader.

### 4.20 Phospholipid Combination Assay

To evaluate the antibacterial activity of peptides in combination with bacterial membrane phospholipids, DOPG, DOPE, and DOPC were selected for joint testing in this study. A checkerboard microdilution assay was used, with serial dilutions of peptides performed along the horizontal axis and serial dilutions of the different phospholipids along the vertical axis. Finally, MIC assay against *E. coli* CMCC 44102 was conducted.

### 4.21 Microscale Thermophoresis Assay

The equilibrium dissociation constants (*K*_d_) of the peptides binding to two fluorescent lipids, NBD‐PG (18:1/12:0) and NBD‐PE (14:0/12:0), were determined by microscale thermophoresis (MST) using a Monolith NT.115 system (NanoTemper Technologies GmbH, Gemmany). Briefly, peptide was subjected to a twofold serial dilution starting from 1000 µm in 20 mm HEPES buffer (pH 7.0). The labeled PG or PE was prepared at a concentration of 250 nm in the same buffer and mixed with each peptide dilution in a 1:1 volume ratio, resulting in a final peptide concentration range of 500–0.0153 µm, Measurements were performed on the Monolith NT.115 instrument using standard treated capillaries under medium MST power and auto‐detected blue excitation power. The *K*_d_ value was calculated from the binding curve using the Monolith Afinity Analysis *K*_d_ fitting model.

### 4.22 Isothermal Titration Calorimetry Assay

The binding afinity between peptide and LPS was measured using isothermal titration calorime‐try (ITC). ITC experiments were performed on a Nano‐ITC (TA, USA) at 25°C. Peptide was prepared at 200 µm in 5 mm HEPES buffer (pH 7.4) and used as the titrant, while LPS was prepared at 20 pm in the same buffer and loaded into the sample cell. The titration protocol consisted of an initial injection of 0.5 µL, followed by 20 injections of 2.5 µL at 160 s intervals, with continuous stirring at 300 rpm. Data were analyzed using PEAQ‐ITC Analysis software, and thermodynamic parameters including enthalpy change (AH), entropy change(AS), and the *K*_d_ was calculated using a one‐site binding model.

### 4.23 Quantitative Determination of Extracellular and Intracellular ATP

The enhanced ATP assay kit was used to measure ATP level. Briefly, *E. coli* CMCC 44102 suspension (OD_600nm_ = 0.5 in PBS) was treated with the peptide at various concentrations for 30 min. The mixture was centrifuged (5000 g, 5 min), and the supernatant was collected for extracellular ATP quantification. The bacterial pellet was lysed with lysozyme, followed by centrifugation to collect the supernatant for intracellular ATP analysis. Chemiluminescencewas measured using a multifunctional microplate reader according to the manufacturer's instructions.

### 4.24 Biofilm Formation Assay

The microtiter dish biofilm formation assay was used to detect the preventive effect of peptides on biofilm formation. TSBG [tryptic soy broth supplemented with 1% (wt./vol) glucose] was used for the growth of bacterial biofilms. In order to prevent the MIC values measured in different media from being inconsistent, the MIC of *S. aureus* ATCC33592 in TSBG was retested, and the result was both 16 µg/mL. The method was improved based on MIC detection. 50 µL of bacterial suspension at 1 × 10^6^ CFU/mL was incubated with the same volume of peptide solution ranging from 1/4×, 1/2×, and 1× MIC in 96‐well microplates. As an untreated control, bacteria were exposed to TSBG medium without peptide. After 24 h of incubation at 37°C, the supernatant was collected, subjected to serial dilution, and plated for colony‐forming unit (CFU) enumeration to quantify the planktonic bacterial population in each well. Gently wash the wells three times with PBS, then dry at 37°C. Biofilms were stained with 0.3% crystal violet (Sigma–Aldrich) for 15 min, washed, and solubilized with 30% acetic acid in water. Quantify absorbance in a plate reader at 550 nm using 30% acetic acid in water as the blank. Biofilm mass was calculated according to the equation:

$$\begin{array}{ccc} & & \mathrm{Biofilm}\;\mathrm{Mass}\;(\%)=[\mathrm{OD}550\;\mathrm{nm}(\mathrm{peptide})-\mathrm{OD}550\;\mathrm{nm}(\mathrm{blank})]\\ & & \;/\;[\mathrm{OD}550\;\mathrm{nm}(\mathrm{negative})-\mathrm{OD}550\;\mathrm{nm}(\mathrm{blank})]\;\times \;100 \end{array}$$

### 4.25 DNA‐AMP Interaction Assay

The DNA‐binding ability of peptides was investigated by gel retardation assay. The genomic DNA of *S. aureus* ATCC33592 was extracted by TIANamp Bacteria DNA Kit. 10 µL DNA was incubated with the same volume of different concentrations of peptide solution (1×–4× MIC in final) dissolved in TE buffer for 30 min. As an untreated control, DNA was exposed to TE buffer without peptide. Subsequently, 4 µL of loading buffer was added, and a 10 µL aliquot was run on a 1% agarose gel. Finally, the migration of DNA bands was observed by ultraviolet (UV) illumination with the ImageQuant 300 gel documentation system.

### 4.26 Cell‐Free Protein Synthesis Inhibition Assay

The reactions were performed using the S30 T7 High‐Yield Protein Expression System (Promega). 3 µL of peptide solution was added to reaction mixtures to get a final concentration of 10 or 20 µg/mL in a final volume of 15 µL in nuclease‐free PCR tubes. Nuclease‐free water was added to the reaction instead of the peptides as an untreated control. Nuclease‐free water was added to the reaction instead of peptides and DNA template as a blank control. Erythromycin was used as a positive control. The samples were incubated for 1 h at 37°C with vigorous mixing (300 rpm), and the reaction was stopped by transferring the samples onto ice. The activity of the Renilla reniformis luciferase, used as a reporter, was quantified using the commercial kit Renilla‐Glo Luciferase Assay System (Promega). 2.5 µL of the reaction mixture was mixed with 97.5 µL buffer and incubated for 10 min before measurement. Black flat‐bottom 96‐well plates were used in a luminometer Plate Camaleon (Bioteck). In all the luminescence measurements, the relative values were calculated as a percentage of the untreated control.

### 4.27 Transcriptome Analysis

*E. coli* CMCC 44102 in logarithmic phase was diluted to 6 × 10^8^ CFU/mL in PBS, then incubated with peptide at 2 × MIC for 2 h at 37°C. Post‐treatment cells were harvested by centrifugation at 5000 g for 5 min at 4°C, and the supematant was discarded. The resulting cell pellets were rapidly frozen in liquid nitrogen and stored at −80°C. RNA was extracted from these samples using TRlzol reagent, followed by DNase l (TaKara) digestion to eliminate any contaminating genomic DNA. The quality and integrity of the extracted RNA were assessed with an Agilent 2100 Bioanalyzer (Agilent TechnologiesCA. USA). Subsequent RNA sequencing and bioinformatic analysis were performed by MajorbioBio‐Pharm Technology Co., Ltd. (Shanghai, China). Construction of the transcriptome libraries was accomplished using the lllu.mina@ Stranded mRNA Prep, Ligation method (San Diego, CA, USA), utilizing total RNAas the starting material. Clean reads obtained from each library were mapped to the referencegenome for further analysis. Genes with an adjusted *p* value < 0.05 were considered statistically significant in differential expression analysis. Each group was conducted with three biological replicates.

### 4.28 In Vivo Experiments

The therapeutic potential of QLX‐3DV‐1 and QLX‐3DV‐2 in vivo was tested in a full‐thickness skin wound model with local *S. aureus* or *E. coli* infection. Ten‐week‐old female C57BL/6J mice were anesthetized using isoflurane and administered buprenorphine as an analgesic at 0.1 mg/kg intraperitoneally. Then, we removed the back hair of mice, and a piece of full‐layer skin with a diameter of 5 mm was removed from the center of the back using a tissue biopsy device. The model was considered as successfully established if the wound area of the mice in each group was uniform. Mice were immediately infected with 5 µL of 10^8^ CFU/mL bacterial solution directly pipetted onto the wound bed. The wound was covered with a sterile dressing. After 5 h, the treatment and control groups were administered 10 µL of 30 mg/mL antimicrobial peptide QLX‐3DV‐1, QLX‐3DV‐2, or SAAP‐148, or 10 µL H_2_O. Mice were sacrificed at the experimental endpoint (24 h postinfection), the wound tissue was collected, homogenized in phosphate‐buffered saline, and plated on solid LB medium. The number of colonies formed on LB medium was counted on the next day.

### 4.29 Safety

For the skin local safety study, the intact or abraded skin of the mice was treated once with antimicrobial peptides QLX‐3DV‐1, QLX‐3DV‐2, or water. In brief, ten‐week‐old female C57BL/6J mice (n = 8 for each group) were anesthetized using isoflurane and administered buprenorphine as an analgesic at 0.1 mg/kg intraperitoneally. After removing the back hair of the mice, the skin on the back of the mice in the scratch group was sanded with 60‐grit sandpaper until the skin oozed blood. The intact skin group received no additional treatment except shaving. 10 µL QLX‐3DV‐1 and QLX‐3DV‐2 (150 mg/mL) or water was applied uniformly over the intact or abraded skin to assess the acute dermal toxicity. Groups without any addition served as untreated controls. Weight changes were recorded over 72 h. Histopathological characteristics of treated skin detected by H&E staining on the third day.

The 10‐week‐old female C57BL/6J mice were randomly divided into three groups (n = 8 for each group). QLX‐3DV‐1, QLX‐3DV‐2, and SAAP‐148 were given to the mice by intraperitoneal injection at 60 mg/kg. Mouse survival was monitored for 96 h. For pathologic analysis, mouse liver and kidneys were obtained by surgery for hematoxylin‐eosin (HE) staining after treatment with 40 mg/kg QLX‐3DV‐1 and QLX‐3DV‐2 for 12 h.

### 4.30 Statistical Analysis

All experiments were repeated at least three times, and GraphPad Prism (version 8) was used for statistical tests. Comparison among groups was performed using two‐tailed Student's t‐test or one‐way ANOVA with Dunnett's multiple comparison test. Significance was accepted when the *p* value was < 0.05.

## Author Contributions

Conceptualization: Y.L., X.L., H.G., Y.W., and Y.Z.; Methodology: X.L., H.G., and Y.W.; Investigation: X.L., H.G., Y.W., L.L., Y.Z., B.W., Y.Z., P.B., Q.K., J.N., Z.H., X.N., K.J., L.S., H.C., and M.Z.; Visualization: X.L., H.G., and Y.W.; Funding acquisition: Y.L.; Project administration: Y.L. and L.L.; Supervision: Y.L., X.Z., X.Y., and Y.Y.; Writing – original draft: X.L.; Writing – review and editing: Y.L., X.L., H.G., and Y.W.

## Funding

National Natural Science Foundation of China grant nos. 82070552 and 82270580 (to Y.L.). National Clinical Research Center for Digestive Diseases supporting technology project grant no. 2015BAI13B07 (to Y.L.). Taishan Scholars Program of Shandong Province (to Y.L.).

## Conflicts of Interest

The authors have filed the following patent applications for this work: (1) Patent No. ZL 202310429545.3 for the method for structural characterisation of peptides based on 3D voxel coloring (inventors: YW, YL, HG, LL, XL, and XZ); (2) Patent No. ZL 202310483081.4 for the method and system for generating and identifying antimicrobial peptides (inventors: YL, YW, HG, XL, LL, and XZ).

## Supporting information




**Supporting File 1**: advs74717‐sup‐0001‐SuppMat.docx.

## Data Availability

All datasets, preprocessing scripts, and source code related to this analysis are available on GitHub at https://github.com/haifangong/M3CAD. Due to file size constraints, additional structural data (e.g., PDB voxelization outputs) are hosted on Baidu Netdisk: https://pan.baidu.com/s/1B‐7nd‐av2oFi‐gQtTd‐Xmg (Access Code: m3cd). To ensure reproducibility, we have provided minimal executable pipelines for both generation (https://github.com/haifangong/M3CAD/blob/main/generation/inference.ipynb) and identification (https://github.com/haifangong/M3CAD/blob/main/identification/inference.ipynb) tasks. The transcriptome sequencing data generated of *E. coli* is available on Mendeley Data (DOI: https://doi.org/10.17632/gvrw23wznb.1).
