# Supplementary material for: De Novo Multi‐Mechanism Antimicrobial Peptide Design via Multimodal Deep Learning
Source: Adv Sci (Weinh). 2026 Mar 9;13(28):e15835. doi: 10.1002/advs.202515835 (PMC13185819; doi:10.1002/advs.202515835)
Supplement: Supplementary file 1 — Supporting File 1: advs74717‐sup‐0001‐SuppMat.docx. [file ADVS-13-e15835-s001.docx]

**Supporting Information**

**Title: *De novo* multi-mechanism antimicrobial peptide design via multimodal deep learning**

**This PDF file includes:**

**Figure S1**. Distribution of characteristics and functional attributes of AMPs in the QLAPD database.

**Figure S2.** UMAP projection of training and generated peptides.

**Figure S3.** Sequence similarity distributions.

**Figure S4.** Length distributions of generated peptides.

**Figure S5.** Percentage of peptides generated by different models that inhibit AMR strains.

**Figure S6**. Hemolysis toxicity of antimicrobial peptide QLX-227-1.

**Figure S7.** Antibiotics sensitivity of ESPAKE including standard and clinical isolates of multidrug-resistant bacteria.

**Figure S8**. Antimicrobial activity of clinical trial peptides against standard and clinical isolates of multidrug-resistant bacteria, represented by MIC (μg/ml).

**Figure S9**. Comparison of antimicrobial activity scores between sequences discovered with and without the 1D model guidance.

**Figure S10**. Experimental off-target toxicity of antimicrobial peptide SAAP-148.

**Figure S11**. Hemolysis (HC25) of QLX-3DV-3–20.

**Figure S12**. Cytotoxicity of QLX-3DV-3–20 against HEK-293T cells at different concentrations.

**Figure S13**. Cytotoxicity (CC50) of QLX-3DV-1–20 on MHCC97-H cell.

**Figure S14**. Cytotoxicity of QLX-3DV-3–20 against MHCC97-H cells at different concentrations.

**Figure S15**. Isothermal calorimetry analysis of the interaction between QLX-3DV-1/2 and lipopolysaccharide (LPS).

**Figure S16**. Microscale thermophoresis analysis of the interaction between QLX-3DV-1/2 and phosphatidylglyc (PG) or phosphatidylethanolamine (PE).

**Figure S17**. In vivo skin safety testing of QLX-3DV-1 and QLX-3DV-2.

**Figure S18**. Optimization of the masking ratio (α) for AMP generation.

**Figure S19**. Validation of synthesized peptide QLX-227-1 and QLX-3DV-1–10.

**Figure S20**. Validation of synthesized peptide QLX-3DV-11–20.

**Figure S21**. Validation of synthesized contrast peptide.

**Figure S22**. Validation of synthesized peptide G1–10.

**Figure S23**. Validation of synthesized peptide GR1–10.

**Figure S24**. Validation of synthesized peptide 1D1–10.

**Figure S25**. Validation of synthesized peptide DT1–10.

**Table S1.** Performance comparison of four structure prediction methods on a wet-lab validated peptide dataset (79 PDBs, length < 50 aa).

**Table S2**. Normalized values of atomic features and amino acid features for the 3D voxel coloring method.

**Table S3**. AP, F1, ACC, and AUC metrics on multilabel classification results of AMP inhibition activity against six types of drug-resistant bacteria.

**Table. S4**. AP, F1, ACC, and AUC metrics on four antimicrobial mechanisms of AMPs.

**Table S5**. AP, F1, ACC, and AUC metrics on multilabel classification of six organ toxicities of AMPs.

**Table S6.** AUC performance comparison across SVM, XGBoost, CatBoost, and M3CAD.

**Table S7**. Comparison of multimodal models vs. single-modality (sequence-only or 3D structure-only) performance on three downstream tasks.

**Table S8.** Single-task (ST) vs multitask (MT) performance across AP, F1, ACC, and AUC for bacterial classes.

**Table S9**. Physicochemical properties of antimicrobial peptide QLX-3DV-1 and QLX-3DV-2 and similarity to training AMPs.

**Table S10**. Experimental MICs of randomly sampled 10 peptides from m2cVAE, the top 10 c_AMPs selected from the regression module and complete pipeline M3CAD-selected 10 c_AMPs.

**Table S11**. Experimental MICs of the top 10 c_AMP selected from the pipeline trained with 3D structural characteristics and sequence, and the top 10 c_AMP selected from the pipeline trained with only sequence.

**Table S12**. Experimental MICs and HC50 value of the top 10 c_AMP selected from the triple-task classifier that includes mechanism, antimicrobial activity, and toxicity, and the top 10 c_AMP selected from the double-task classifier that includes antimicrobial activity and toxicity.

**Table S13**. Physicochemical properties of training AMPs and newly discovered AMPs using the M3-CAD pipeline.

**Table S14.** Therapeutic index (CC50/geometric mean MIC) of QLX-3DV-1–20, and SAAP-148 against various bacterial species on HEK-293T.

**Table S15.** Therapeutic index (CC50/geometric mean MIC) of QLX-3DV-1–20, and SAAP-148 against various bacterial species on MHCC97-H.

**Table S16**. MICs of AMPs and cefepime (tested alone or in combination) and FICIs for the combinations of AMPs and cefepime.

**Table S17**. Sequences of de novo designed peptides QLX-3DV-1–20 by M3CAD.

**Table S18**. Sequences of contrast peptides.

**Table S19**. Sequences of Randomly sampled 10 peptides from m2cVAE(Generation).

**Table S20**. Sequences of Regression moudle-selected 10 c_AMPs(Generation+Regression).


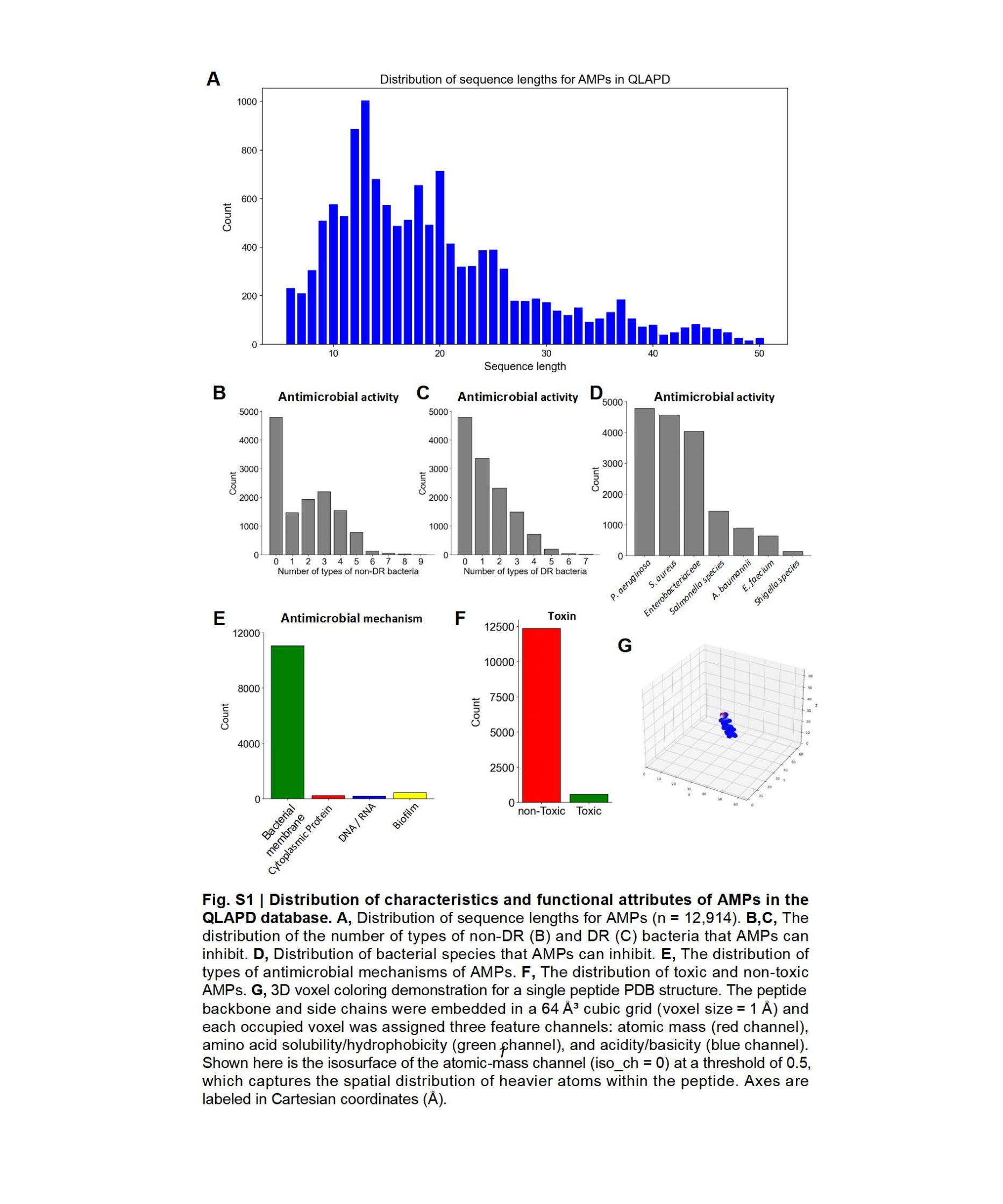


**Figure S1. Distribution of characteristics and functional attributes of AMPs in the QLAPD database.**

**(A)** Distribution of sequence lengths for AMPs (n = 12,914).

**(B, C)** Distribution of the number of types of non-DR (B) and DR (C) bacterial types that AMPs can inhibit.

**(D)** Distribution of bacterial species that AMPs can inhibit.

**(E)** Distribution of types of antimicrobial mechanisms of AMPs.

**(F)** The distribution of toxic and nontoxic AMPs.

**(G)** 3D voxel coloring demonstration for a single peptide PDB structure. The peptide backbone and side chains were embedded in a 64 Å³ cubic grid (voxel size = 1 Å), and each occupied voxel was assigned three feature channels: atomic mass (red channel), amino acid solubility/hydrophobicity (green channel), and acidity/basicity (blue channel). Shown here is the isosurface of the atomic-mass channel (iso_ch = 0) at a threshold of 0.5, which captures the spatial distribution of heavier atoms within the peptide. Axes are labelled in Cartesian coordinates (Å).

**
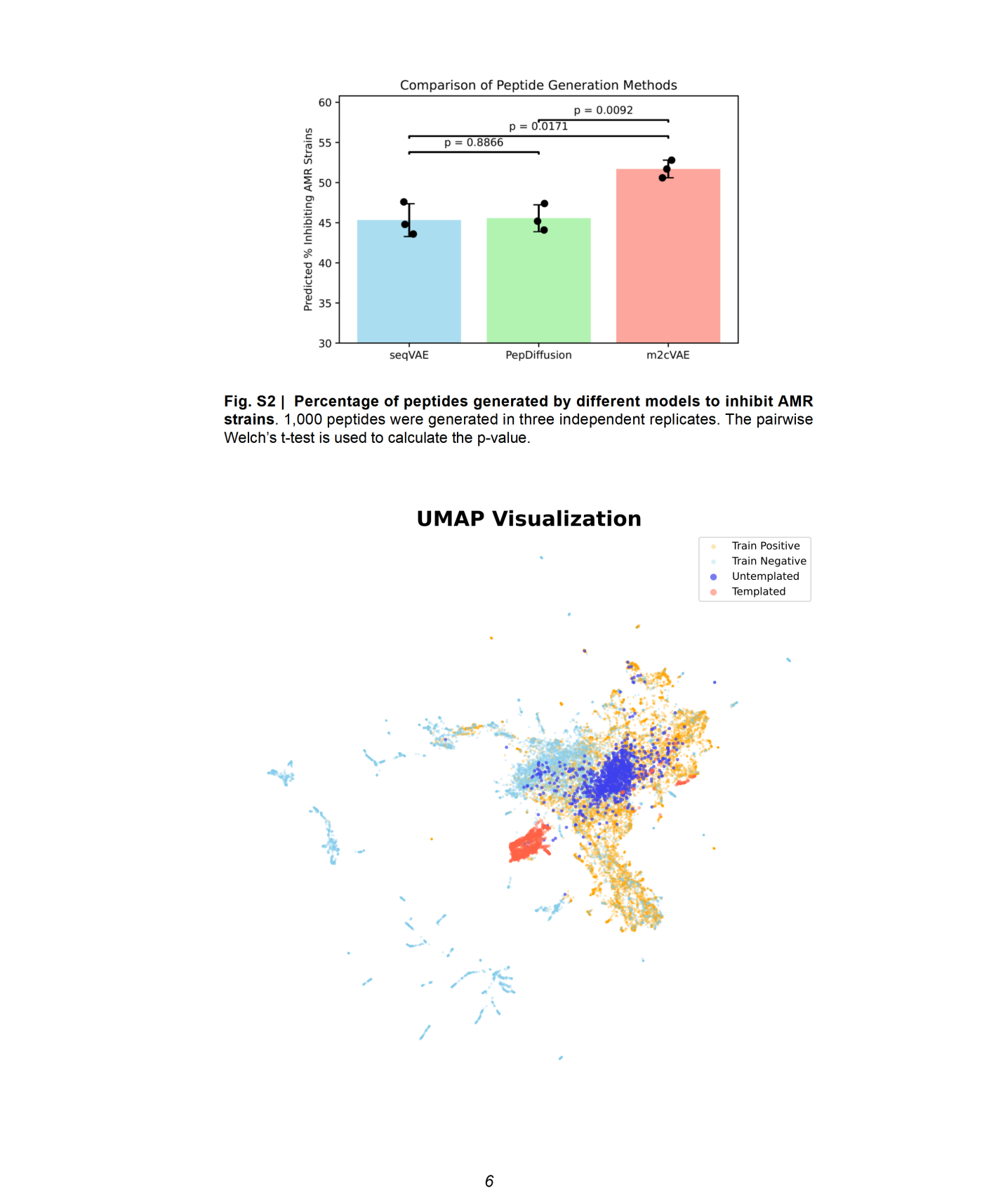
**

**Figure S2. UMAP projection of training and generated peptides.**

All positive training set (gold), negative training set (blue), top 1,000 untemplated candidates (purple), and top 1,000 templated candidates (red) are embeded into two dimensions. Templated peptides form one tight cluster, whereas untemplated peptides are dispersed throughout the space and overlap with both train positive/negative regions, indicating a wider coverage of the learned embedding.


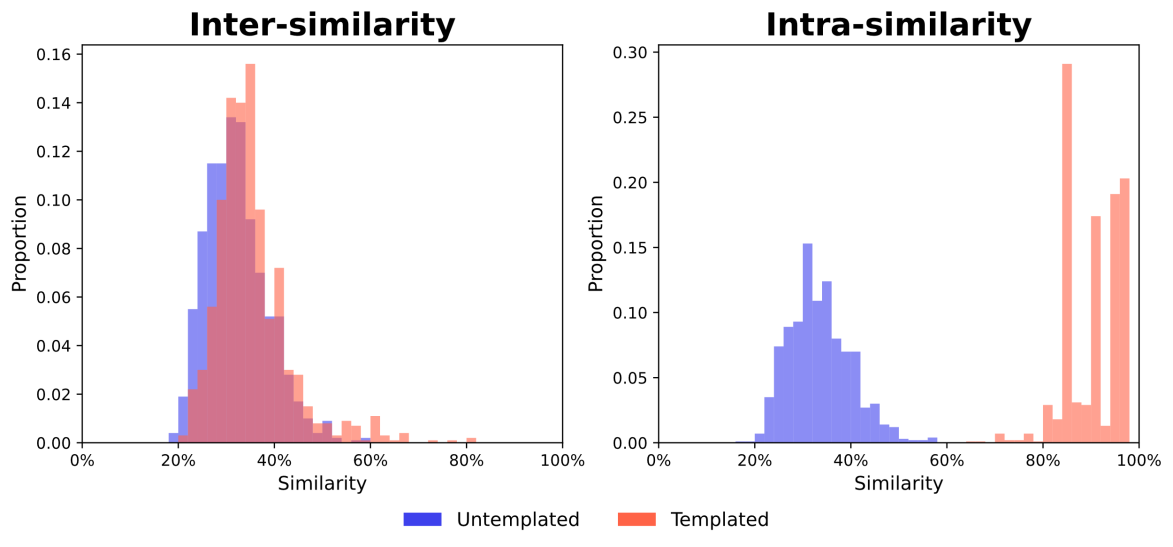


**Figure S3. Sequence similarity distributions.**

Left: Analysis of sequence similarity between the generated peptides and the training set shows that both untemplated (blue) and templated (pink) sequences maintain sequence similarity below 50%, confirming their overall novelty.

Right: Intra-similarity among generated peptides. Untemplated peptides peak around 30–50% identity, while templated peptides cluster at 80–100%, indicating that templated generation largely performs small, local mutations on existing motifs.


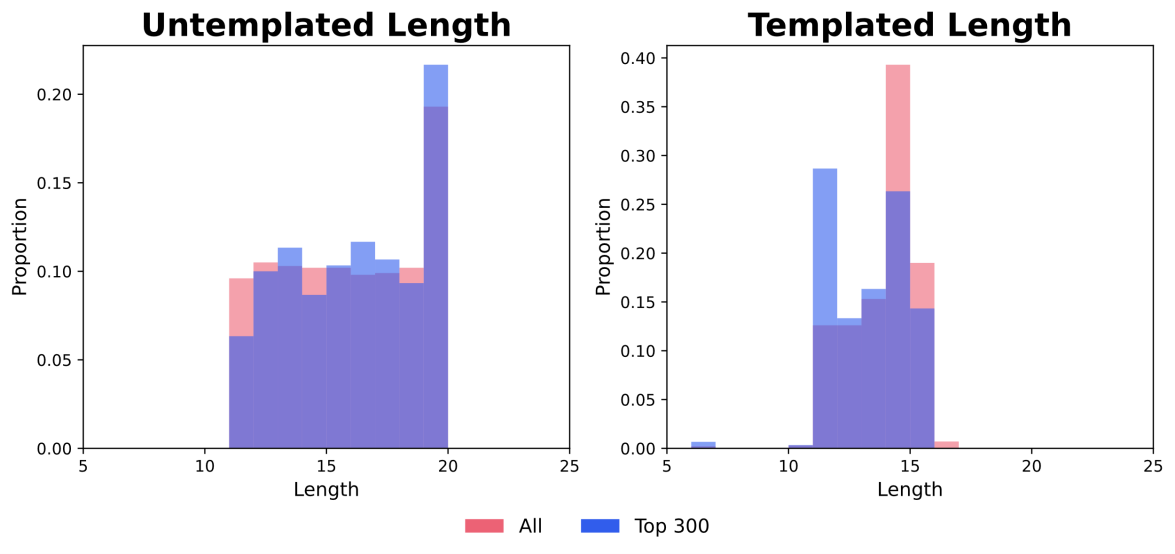


**Figure S4. Length distributions of generated peptides.**

Left: Histogram of full (pink) versus top-300 (blue) untemplated peptides, showing lengths from 10-24 residues and retention of broad length diversity after filtering.

Right: Histogram of full (pink) versus top-300 (blue) templated peptides, demonstrating a tight length range of 14-18 residues—reflecting our design choice to optimize for synthetic feasibility, structural stability, and membrane-penetration, and explaining the high sequence similarity observed in templated candidates.


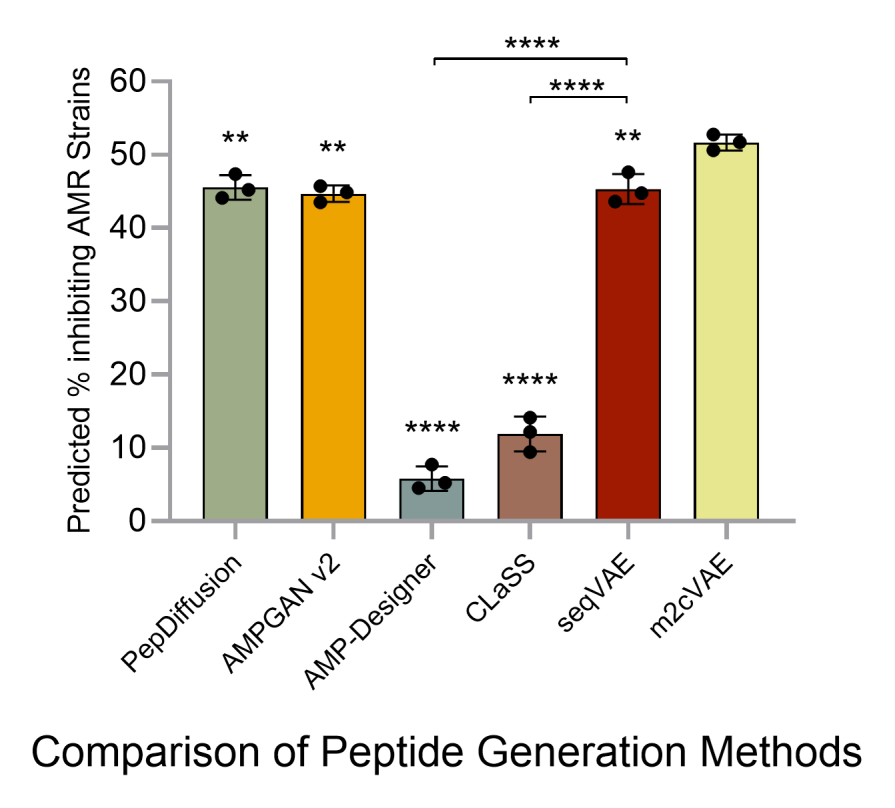


**Figure S5. Percentage of peptides generated by different models that inhibit AMR strains.**

1,000 peptides were generated in three independent replicates. All data are mean ± SD. All p-values were calculated and reported using a two-tailed Student’s t-test. **P < 0.01, ****P < 0.0001.


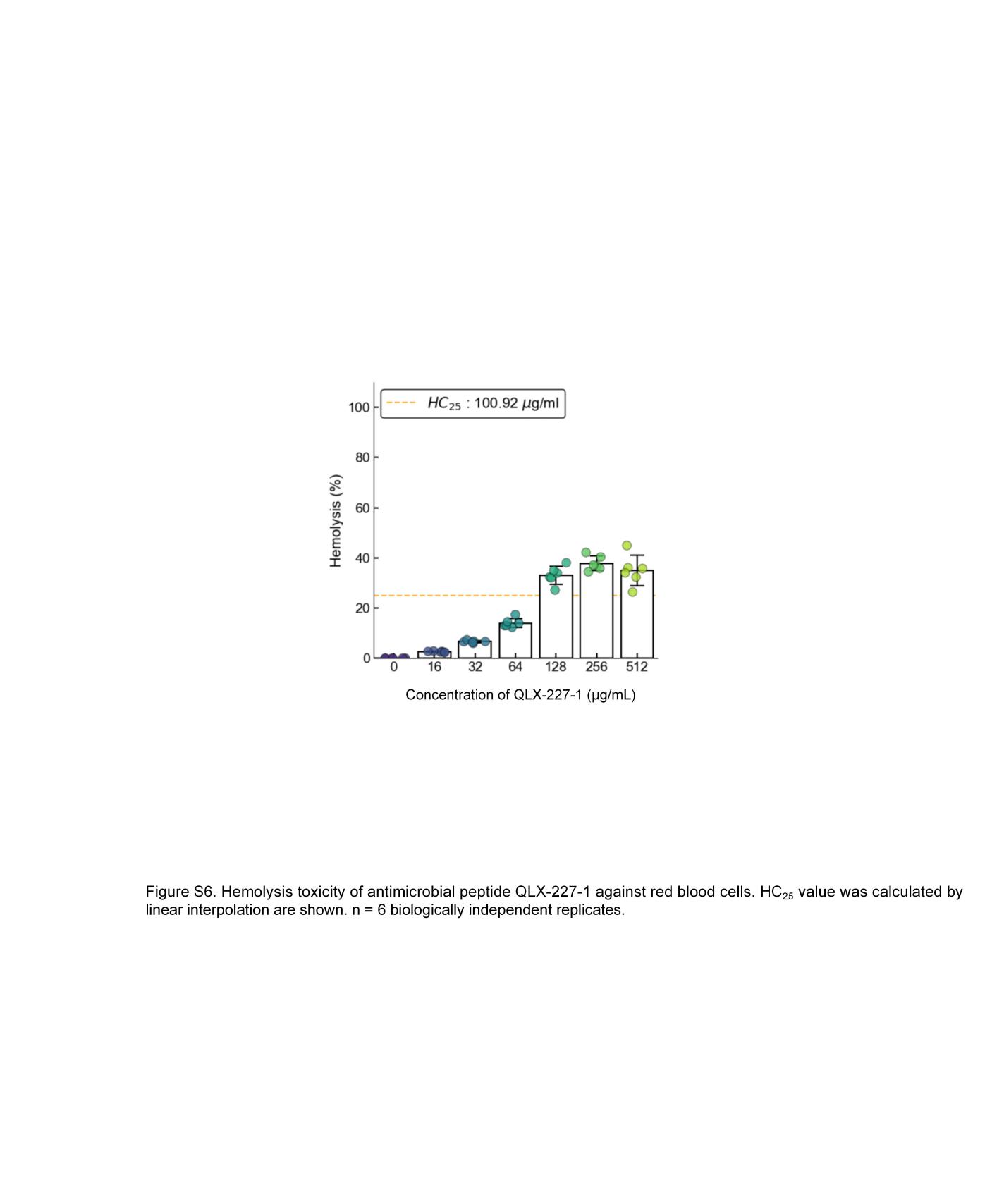


**Figure S6. Hemolysis toxicity of antimicrobial peptide QLX-227-1.** n = 6 biologically independent replicates.


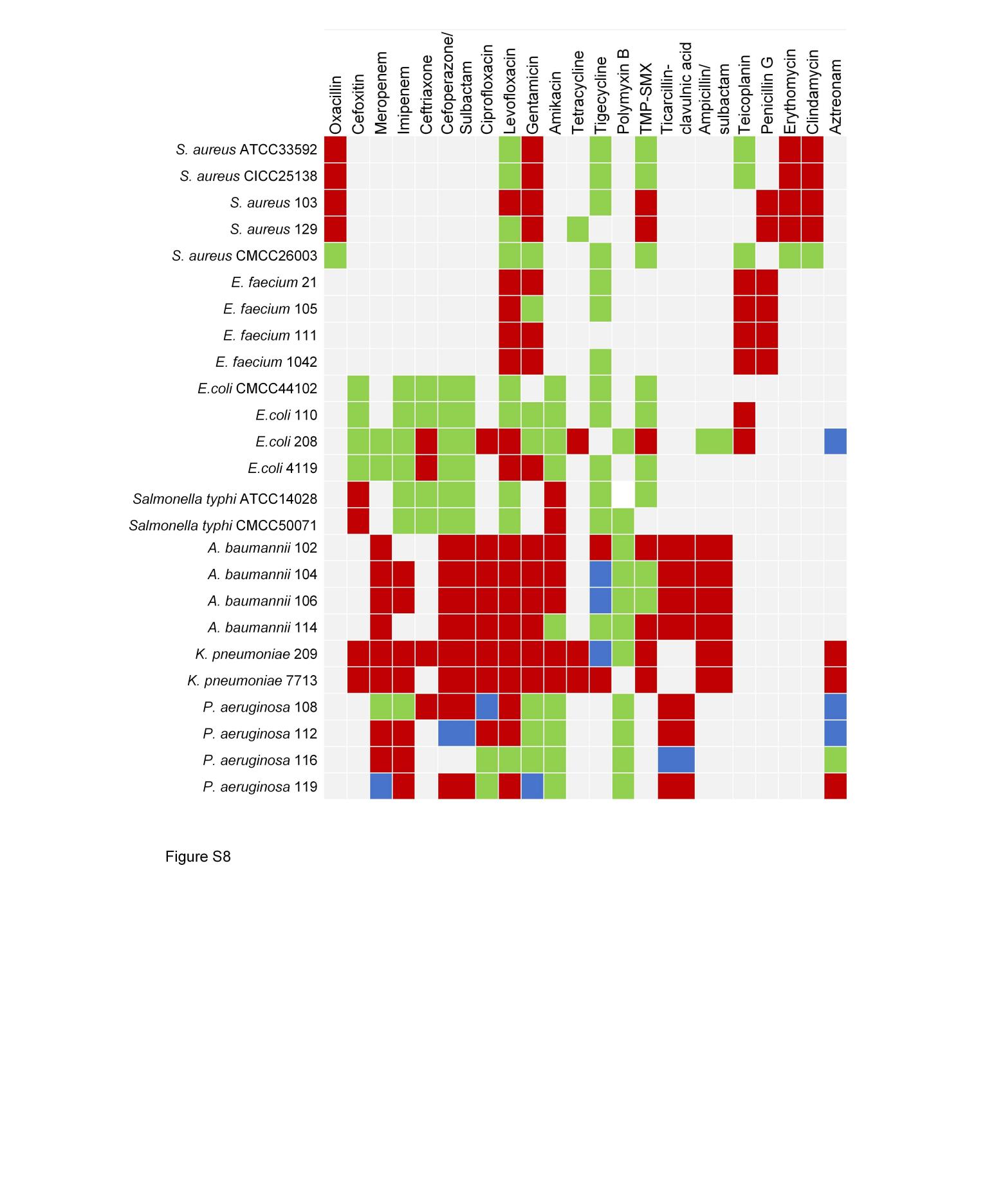


**Figure S7. Antibiotics sensitivity of ESPAKE including standard and clinical isolates of multidrug-resistant bacteria.**

Explanatory note: Green, red, and blue boxes represent clinically isolated strains that are susceptible, resistant, and moderately susceptible to the corresponding antibiotics, respectively. Gray boxes are shown if the susceptibility to agents in that class is not assessed.


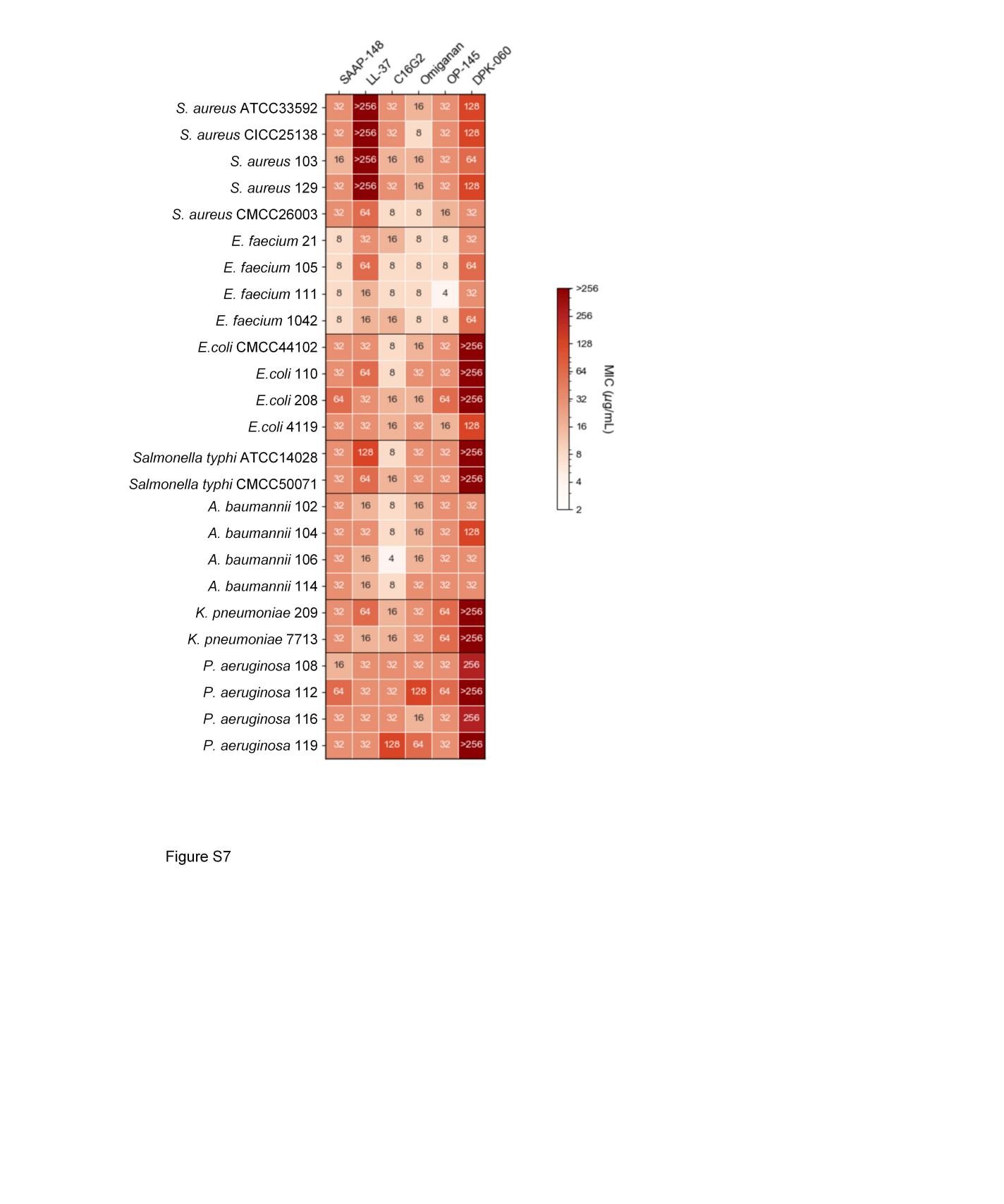


**Figure S8. Antimicrobial activity of clinical trial peptides against standard and clinical isolates of multidrug-resistant bacteria, represented by MIC (μg/ml); each result had at least 3 biological replicates.**


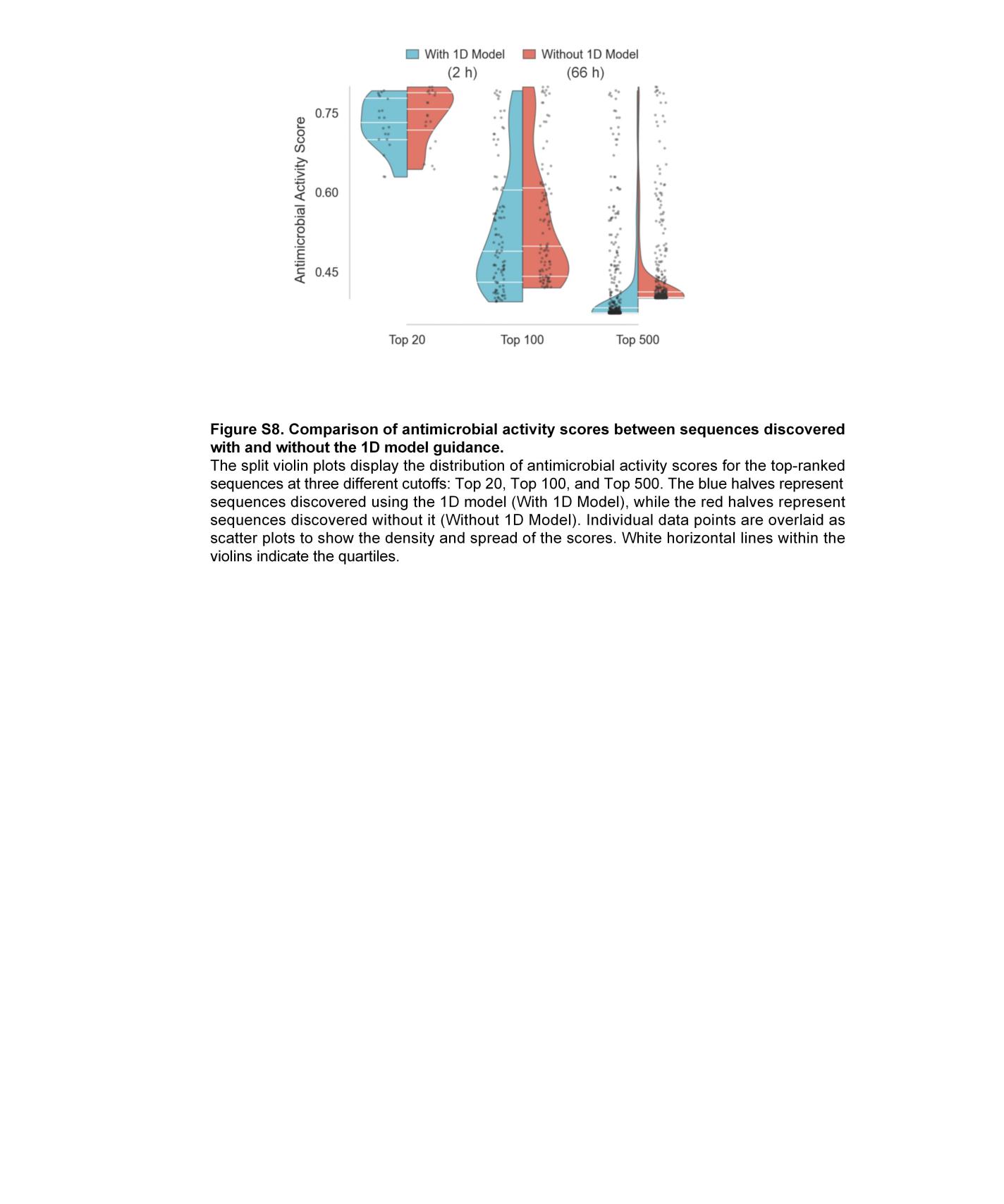


**Figure S9. Comparison of antimicrobial activity scores between sequences discovered with and without the 1D model guidance.**

The split violin plots display the distribution of antimicrobial activity scores for the top-ranked sequences at three different cutoffs: Top 20, Top 100, and Top 500. The blue halves represent sequences discovered using the 1D model (With 1D Model), while the red halves represent sequences discovered without it (Without 1D Model). Individual data points are overlaid as scatter plots to show the density and spread of the scores. White horizontal lines within the violins indicate the quartiles.


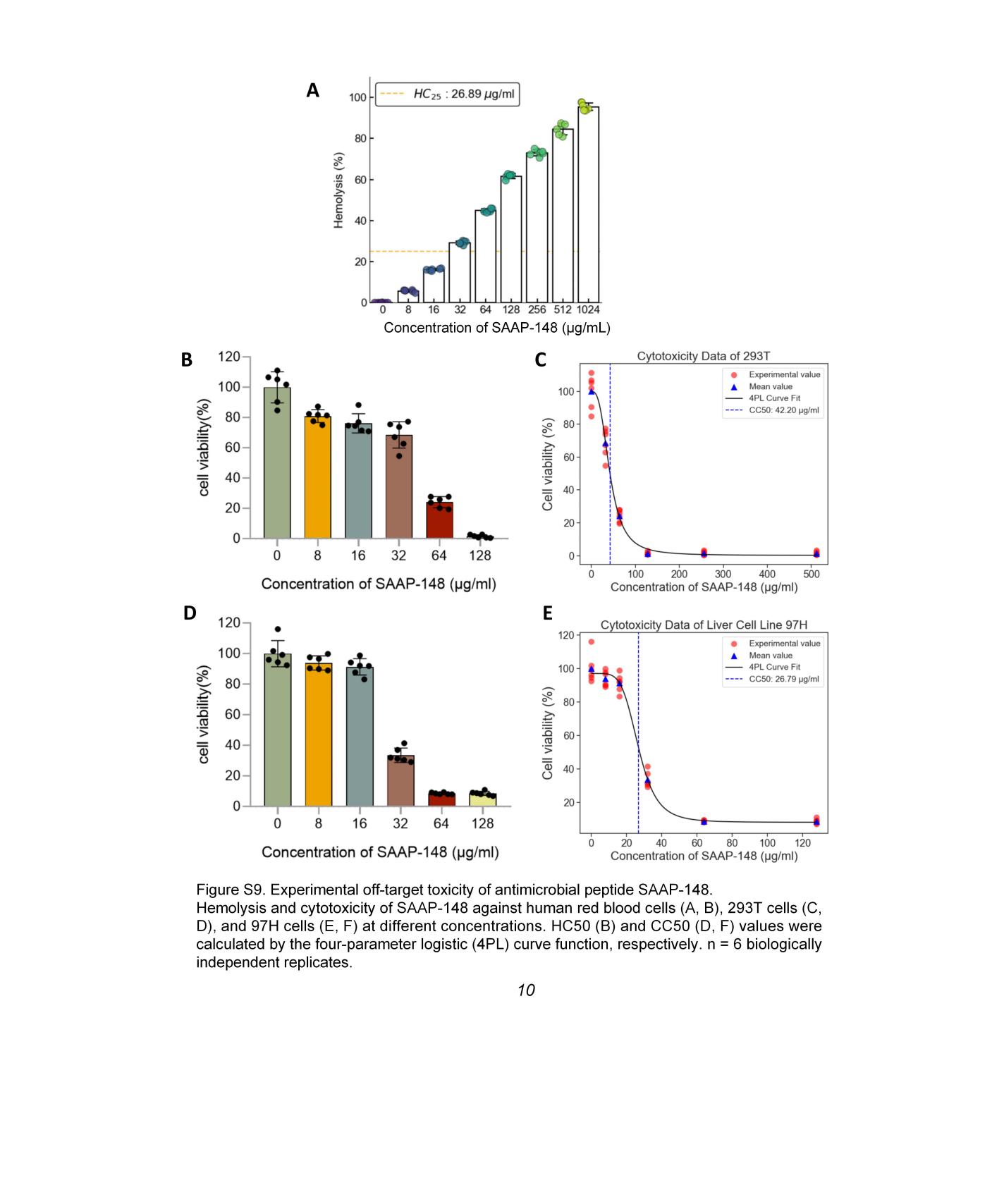


**Figure S10. Experimental off-target toxicity of antimicrobial peptide SAAP-148.**

Hemolysis and cytotoxicity of SAAP-148 against human red blood cells (A), 293T cells (B, C), and 97H cells (D, E) at different concentrations. HC_25_ (A) values were calculated using linear interpolation. CC_50_ (C, E) values were calculated by the four-parameter logistic (4PL) curve function, respectively. n = 6 biologically independent replicates.


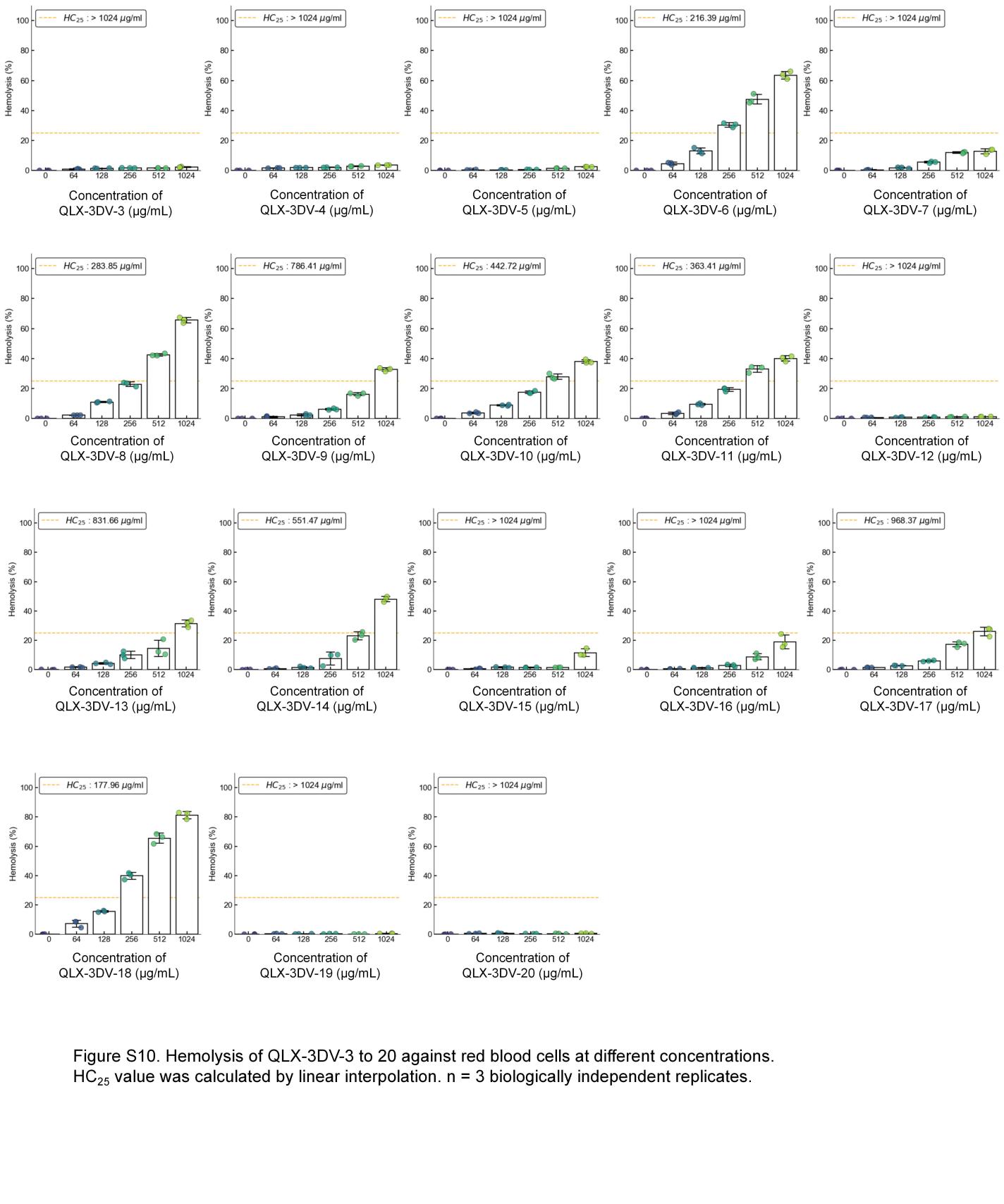


**Figure S11. Hemolysis (HC25) of QLX-3DV-3–20.** HC_25_ values were calculated using linear interpolation. n = 3 biologically independent replicates.


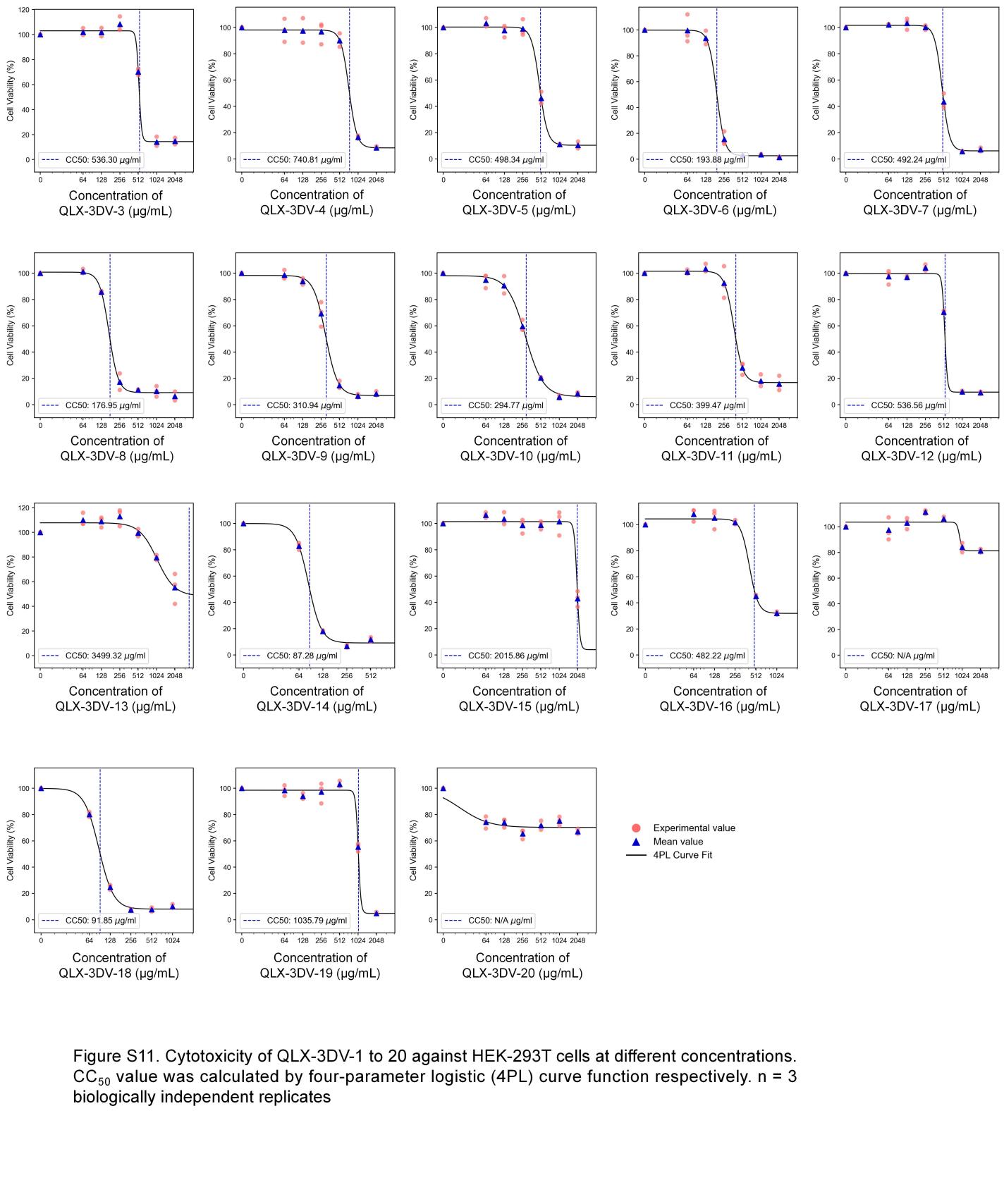


**Figure S12. Cytotoxicity of QLX-3DV-3–20 against HEK-293T cells at different concentrations.** CC_50_ values were calculated using four-parameter logistic (4PL) curve function. n = 3 biologically independent replicates.


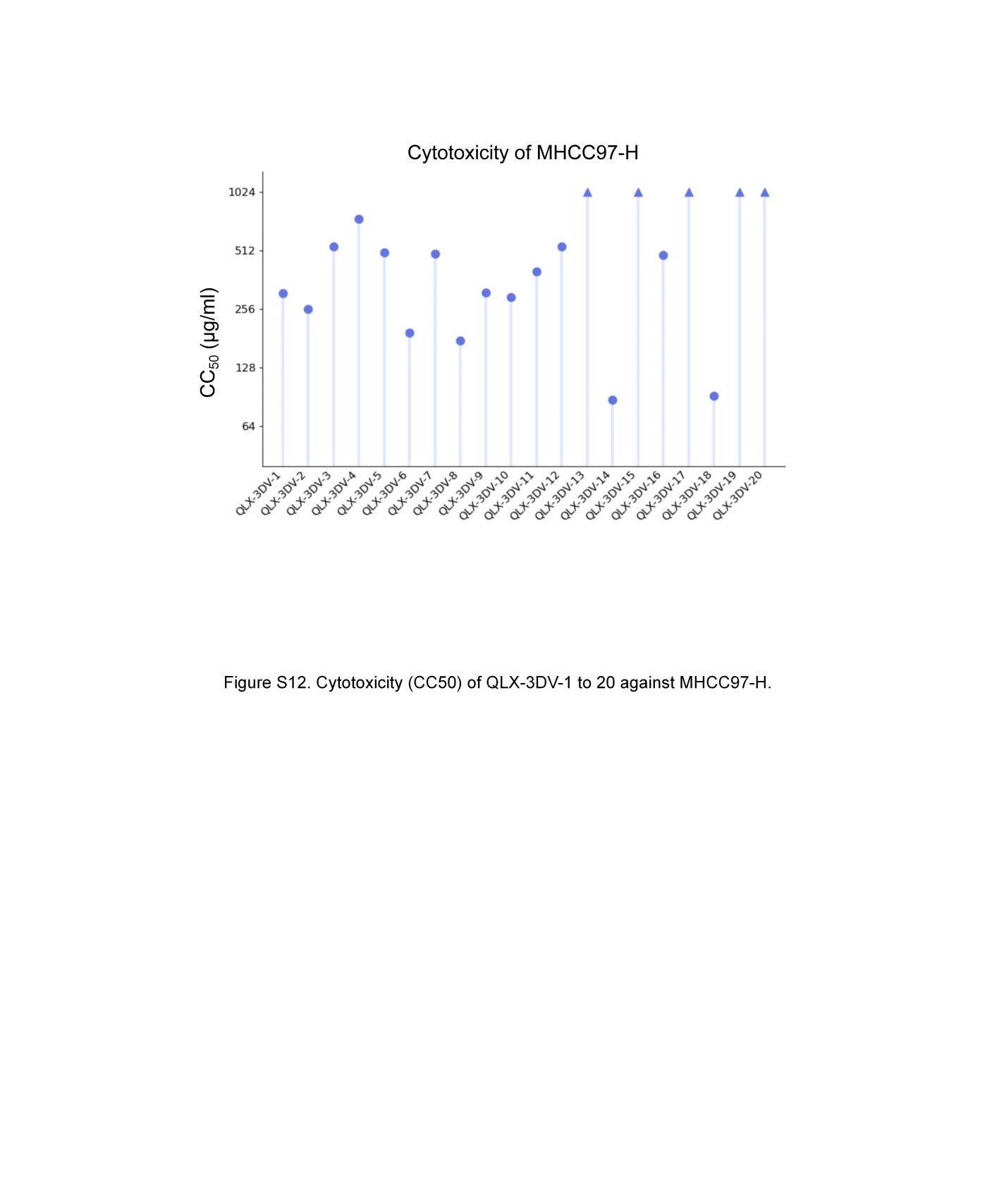


**Figure S13. Cytotoxicity (CC_50_) of QLX-3DV-1–20 on MHCC97-H cell.** CC_50_ values were calculated using four-parameter logistic (4PL) curve function. n = 3 biologically independent replicates.


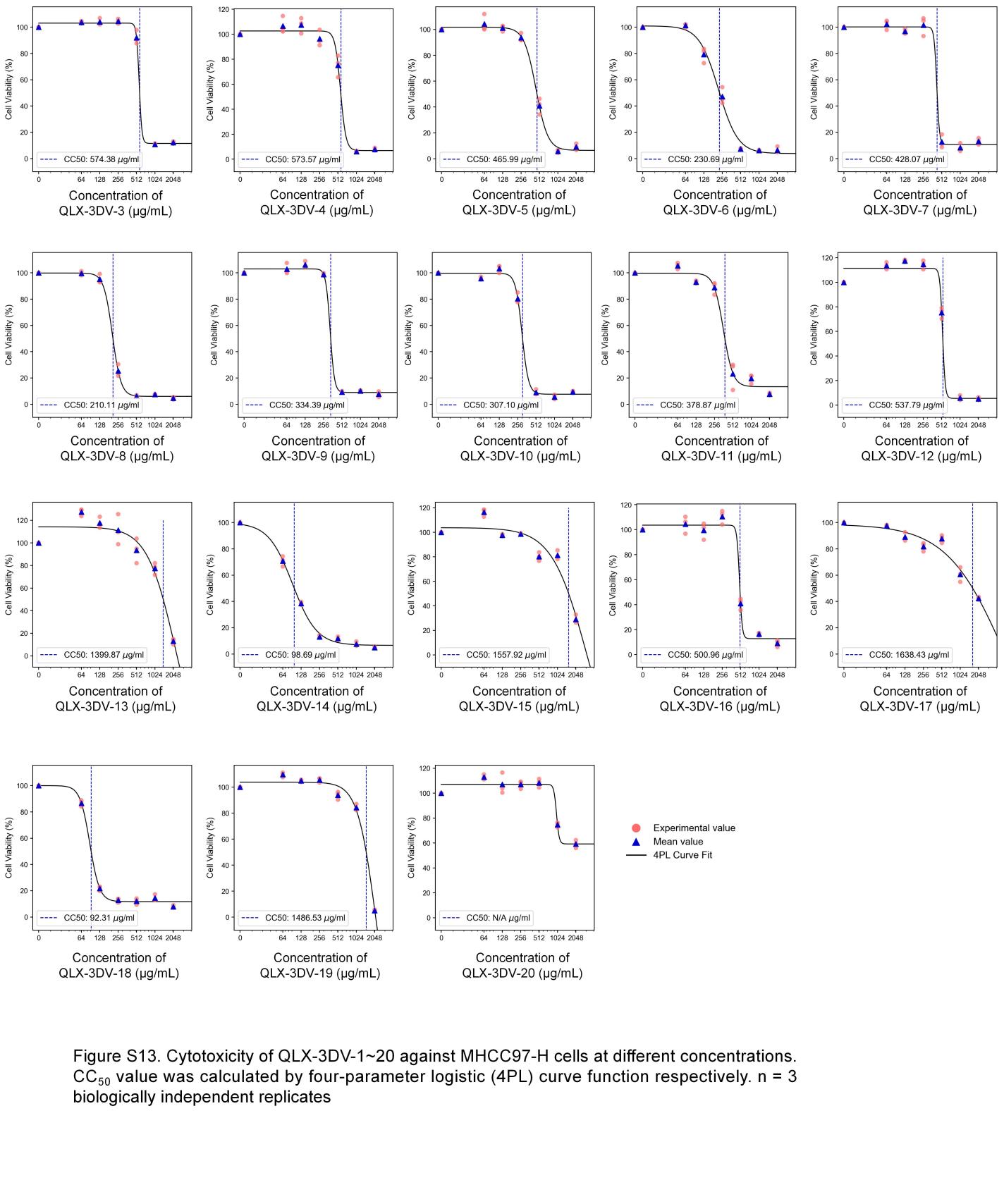


**Figure S14. Cytotoxicity of QLX-3DV-3–20 against MHCC97-H cells at different concentrations.** CC_50_ values were calculated using four-parameter logistic (4PL) curve function. n = 3 biologically independent replicates.


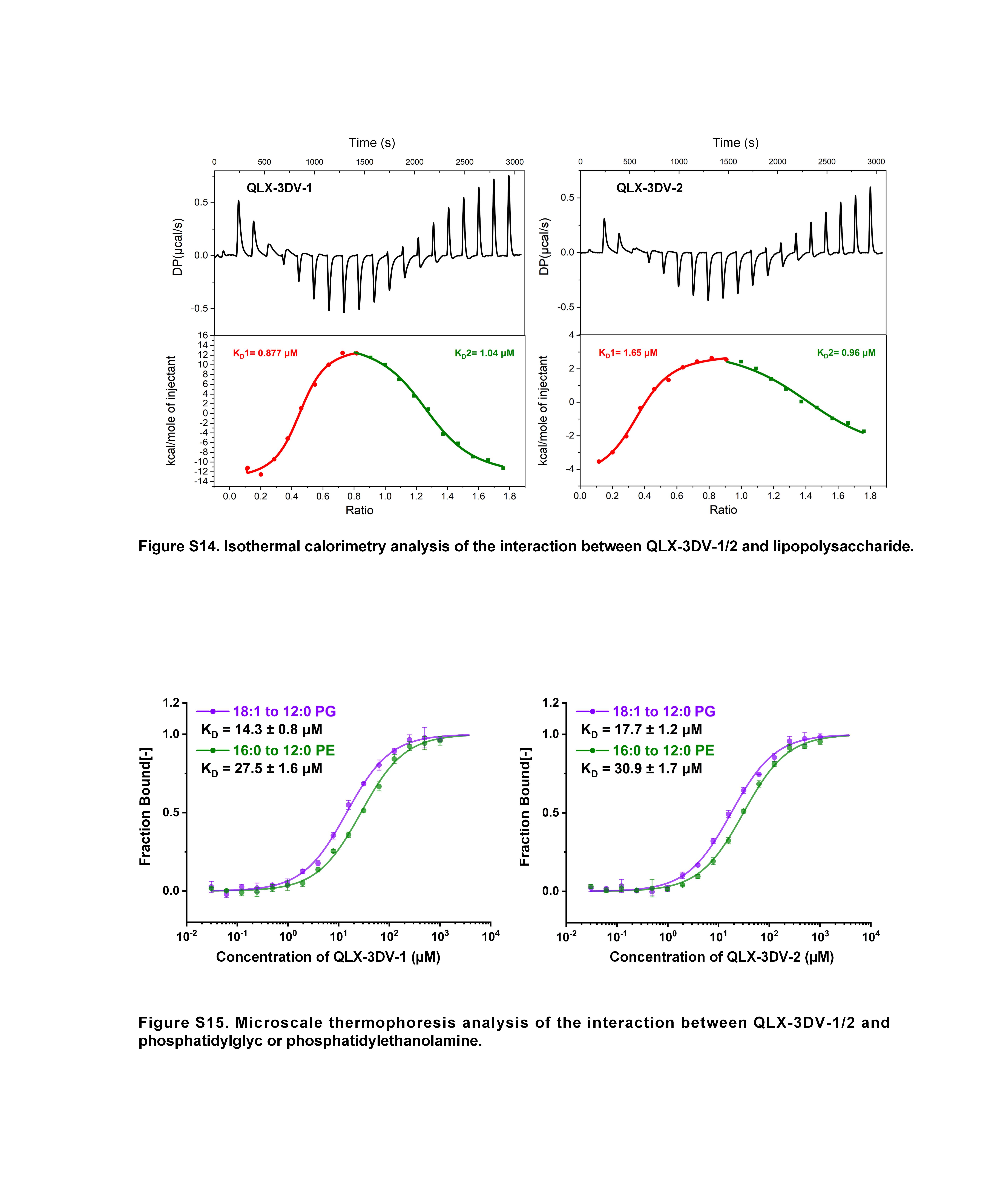


**Figure S15. Isothermal calorimetry analysis of the interaction between QLX-3DV-1/2 and lipopolysaccharide (LPS).**

The equilibrium dissociation constant (Kd) value is shown.


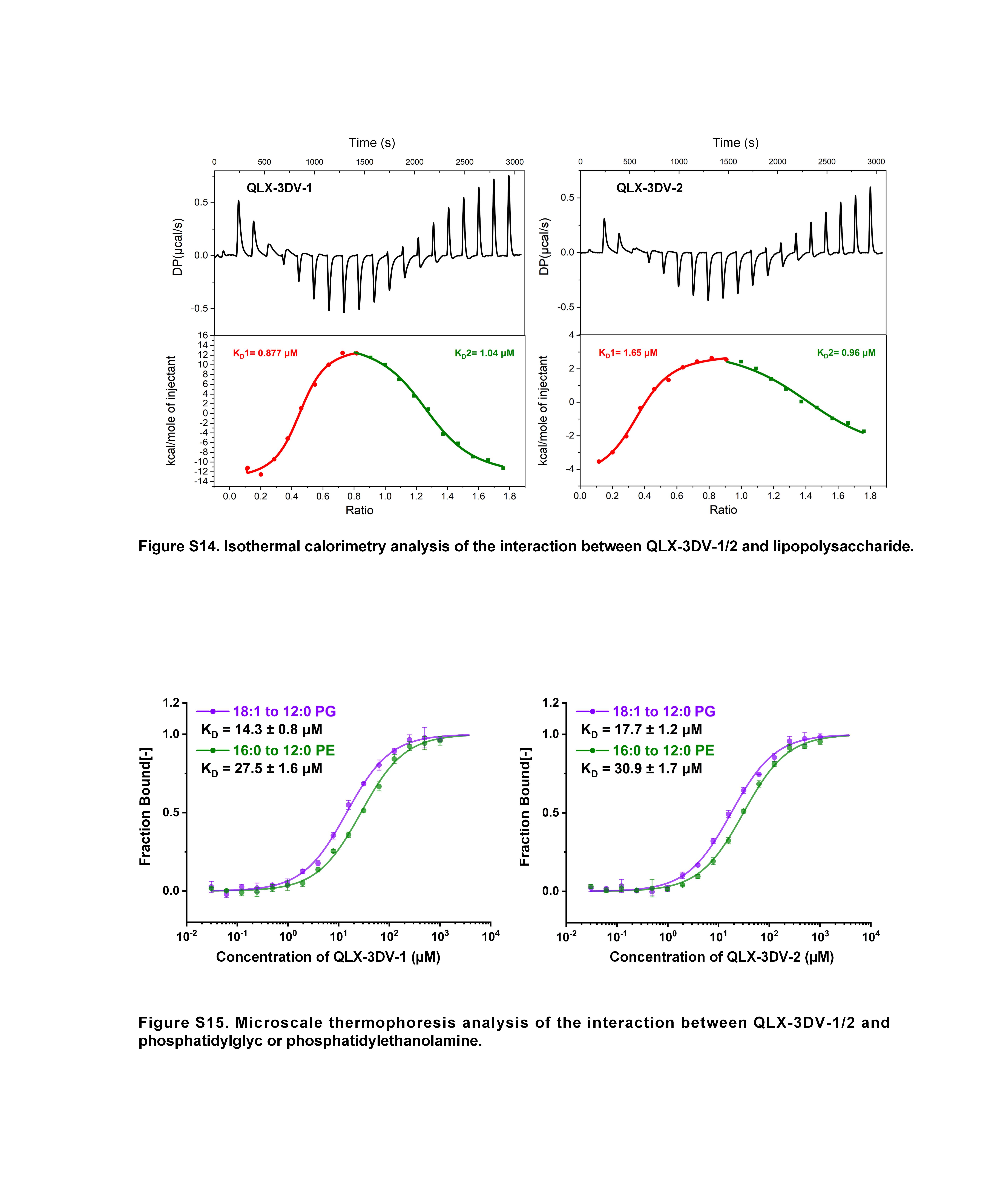


**Figure S16. Microscale thermophoresis analysis of the interaction between QLX-3DV-1/2 and phosphatidylglyc (PG) or phosphatidylethanolamine (PE).**

The equilibrium dissociation constant (Kd) value is shown.


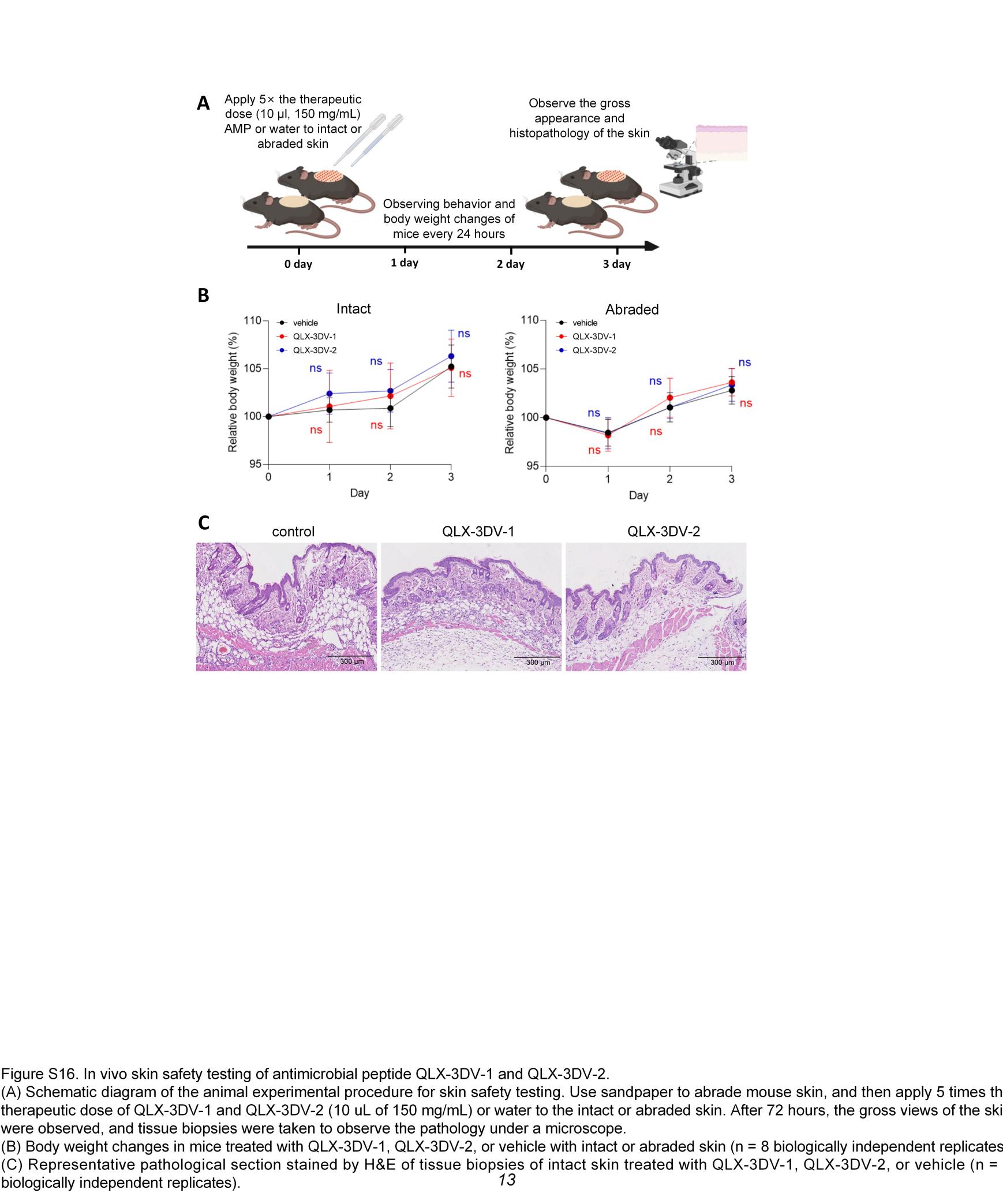


**Figure S17. *In vivo* skin safety testing of QLX-3DV-1 and QLX-3DV-2.**

**(A)** Schematic diagram of the animal experimental procedure for skin safety testing. Use sandpaper to abrade mouse skin, and then apply 5 times the therapeutic dose of QLX-3DV-1 and QLX-3DV-2 (10 μL of 150 mg/mL) or water to the intact or abraded skin. After 72 hours, the gross views of the skin were observed, and tissue biopsies were taken to observe the pathology under a microscope.

**(B)** Body weight changes in mice treated with QLX-3DV-1, QLX-3DV-2, or vehicle with intact or abraded skin (n = 8 biologically independent replicates).

**(C)** Representative H&E-stained histopathological sections of tissue biopsies of intact skin treated with QLX-3DV-1, QLX-3DV-2, or vehicle (n = 8 biologically independent replicates).


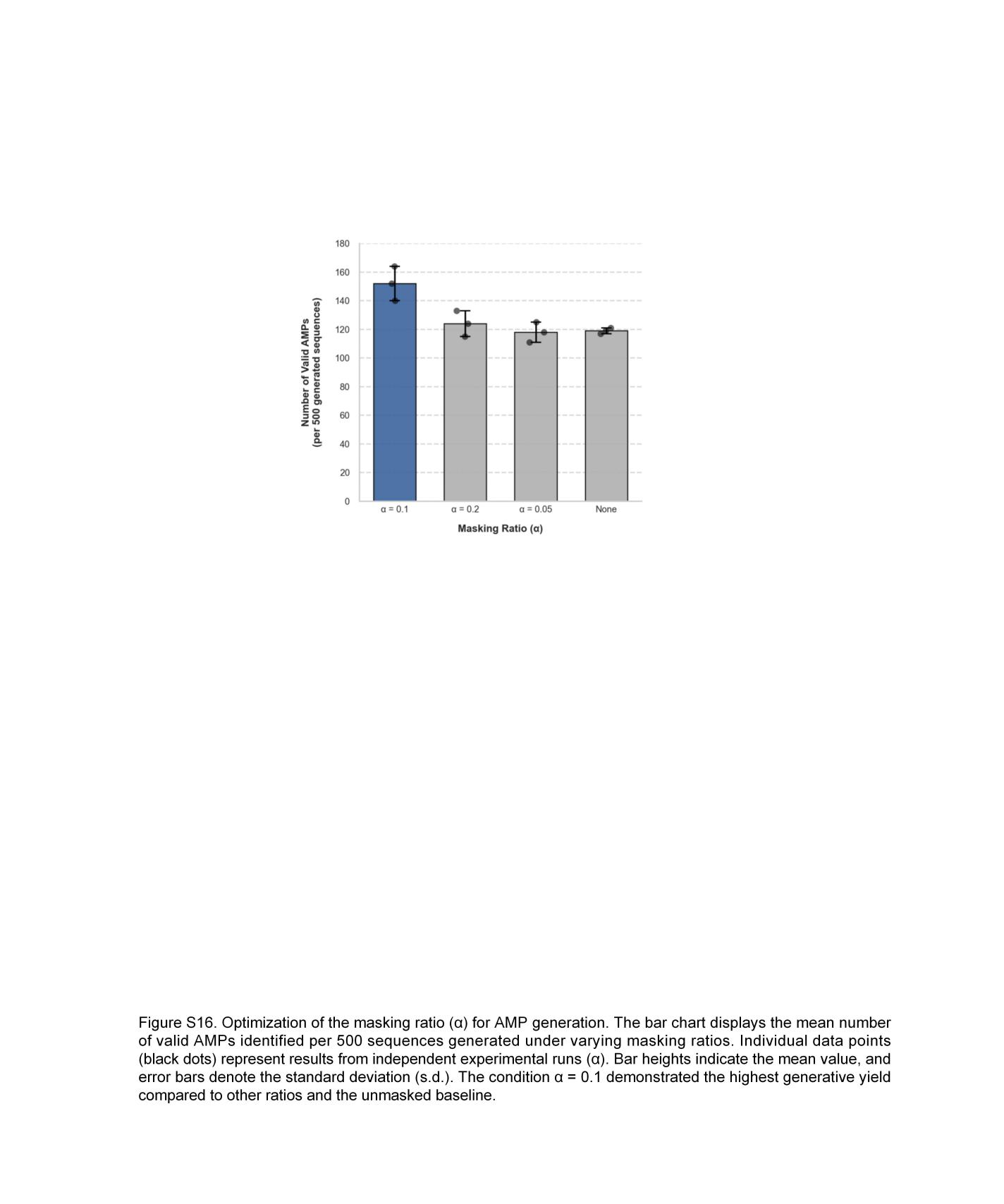


**Figure S18. Optimization of the masking ratio (α) for AMP generation.**

The bar chart displays the mean number of valid AMPs identified per 500 sequences generated under varying masking ratios. Individual data points (black dots) represent results from independent experimental runs (α). Bar heights indicate the mean value, and error bars denote the standard deviation (s.d.). The condition α = 0.1 demonstrated the highest generative yield compared to other ratios and the unmasked baseline.


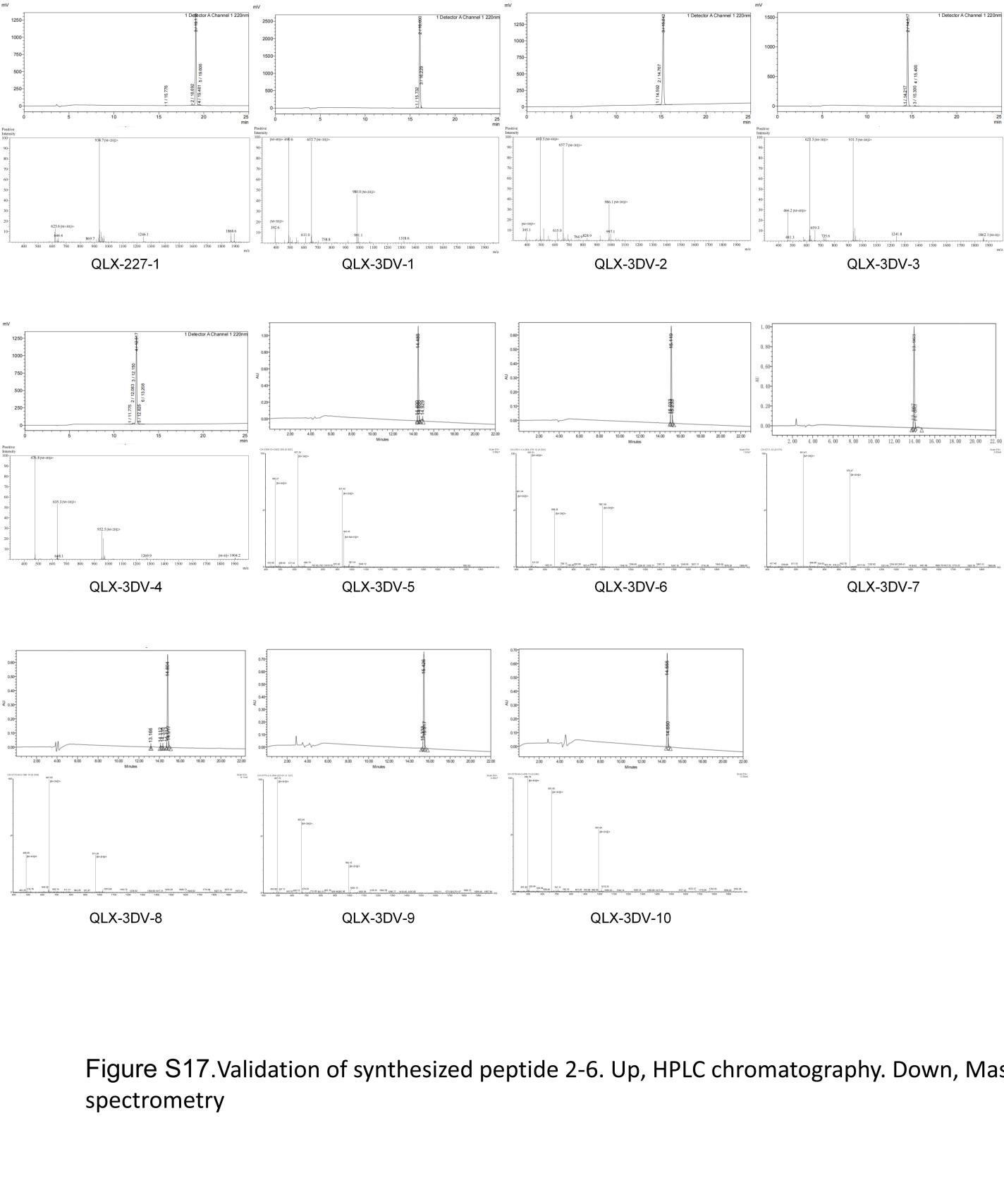


**Figure S19.Validation of synthesized peptide QLX-227-1 and QLX-3DV-1–10.**

Up, High-performance liquid chromatography (HPLC). Down, Mass spectrometry (MS).


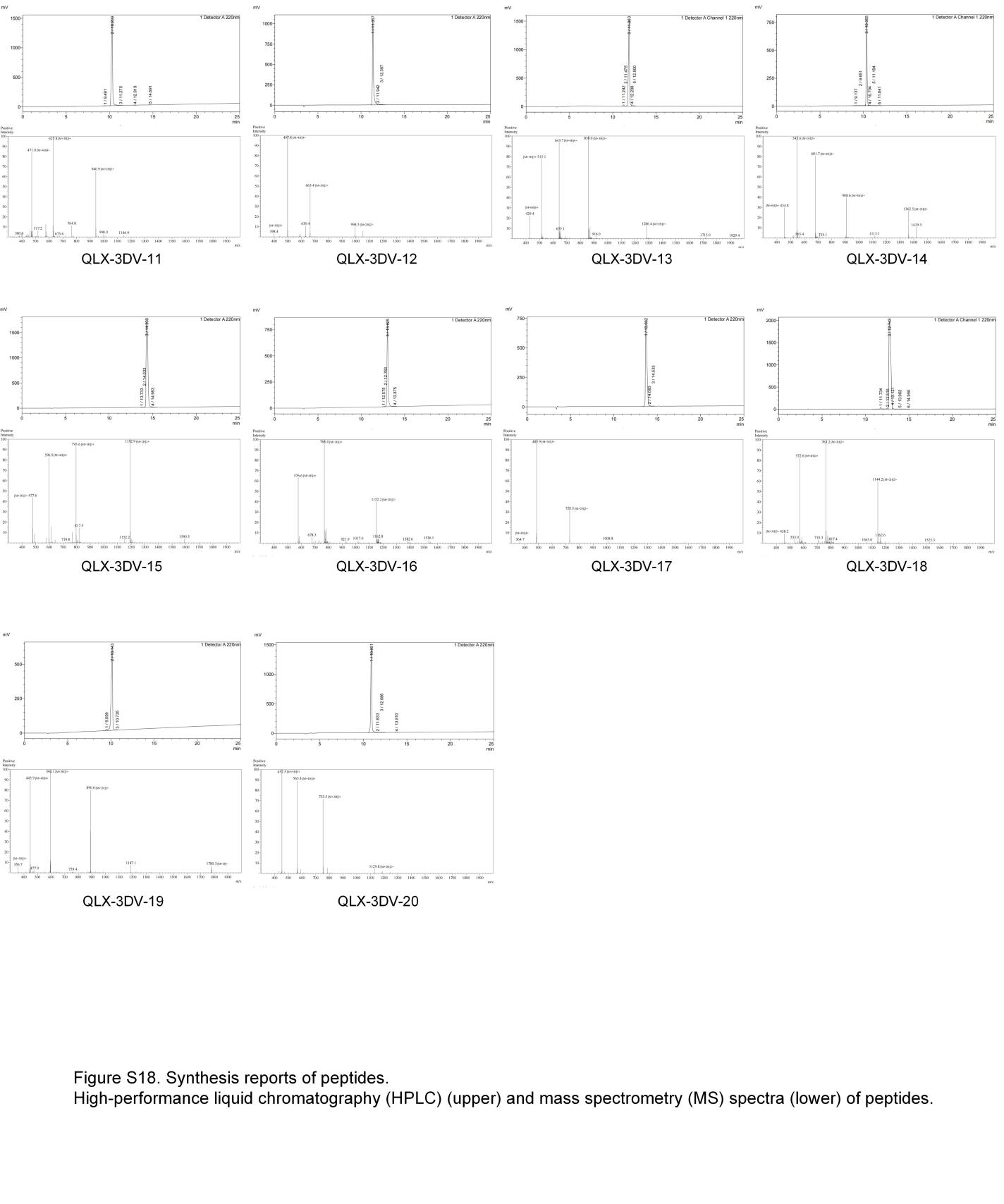


**Figure S20.Validation of synthesized peptide QLX-3DV-11–20.**

Up, High-performance liquid chromatography (HPLC). Down, Mass spectrometry (MS).


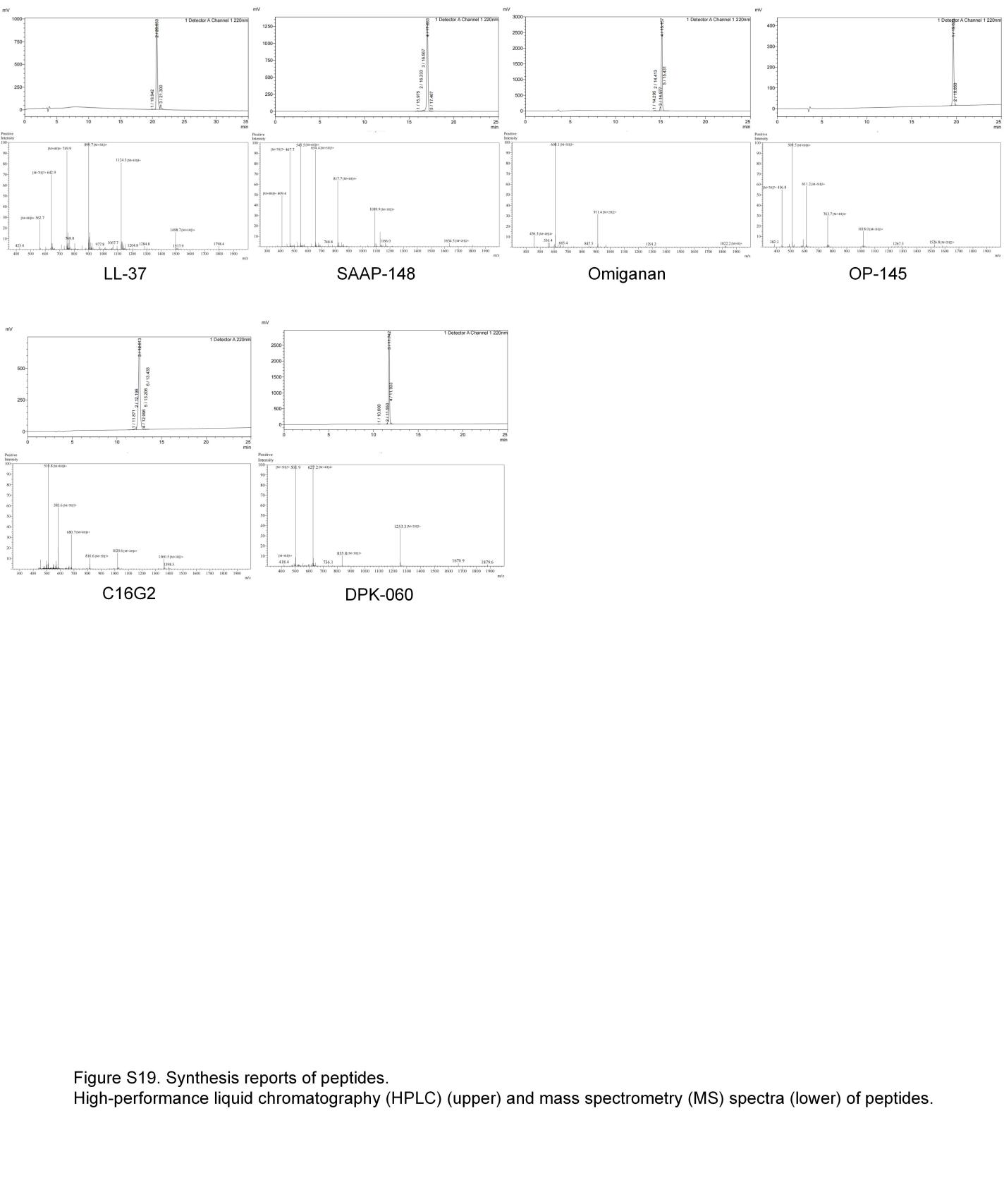


**Figure S21.Validation of synthesized contrast peptide.**

Up, High-performance liquid chromatography (HPLC). Down, Mass spectrometry (MS).


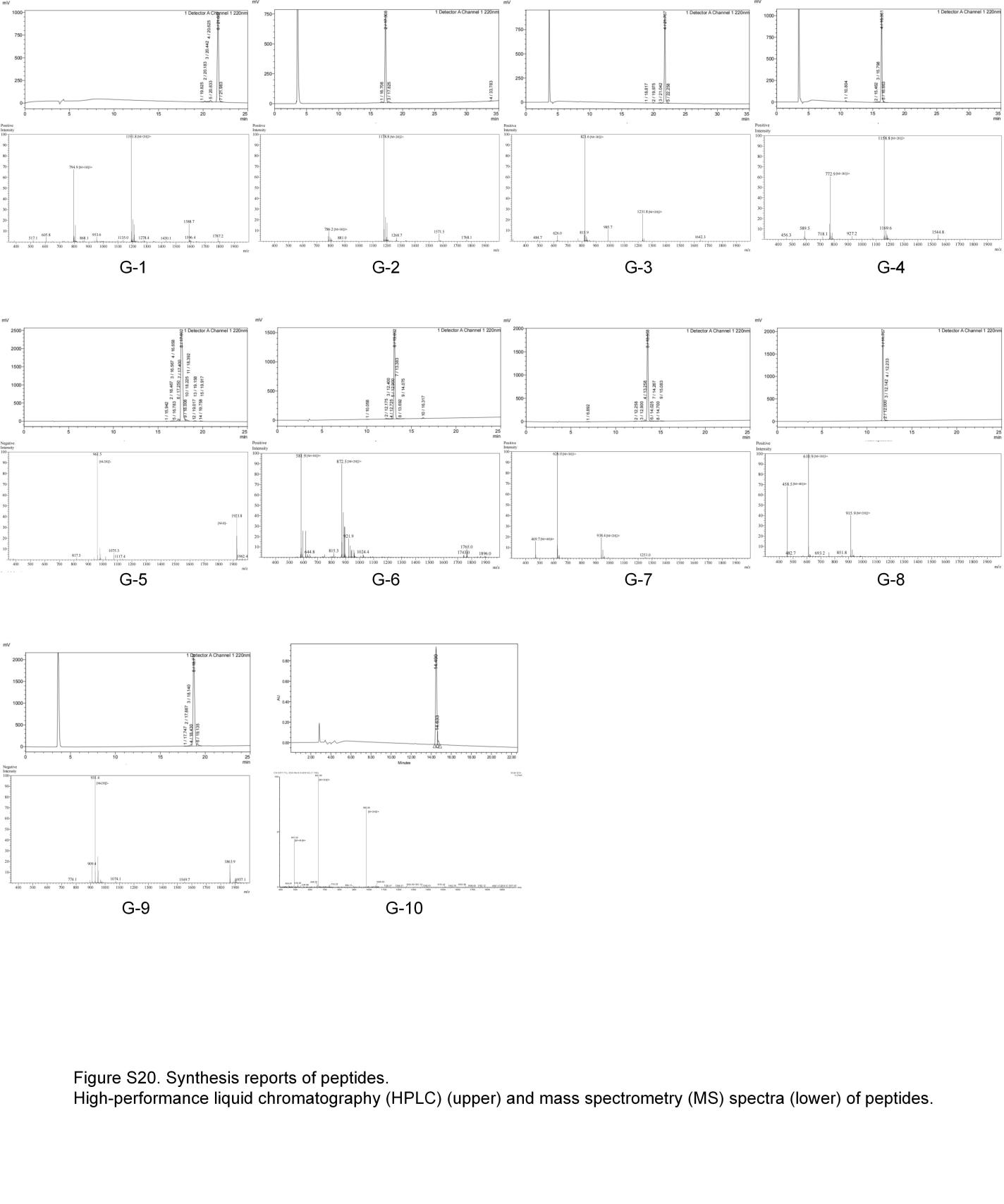


**Figure S22.Validation of synthesized peptide G1–10.**

Up, High-performance liquid chromatography (HPLC). Down, Mass spectrometry (MS).


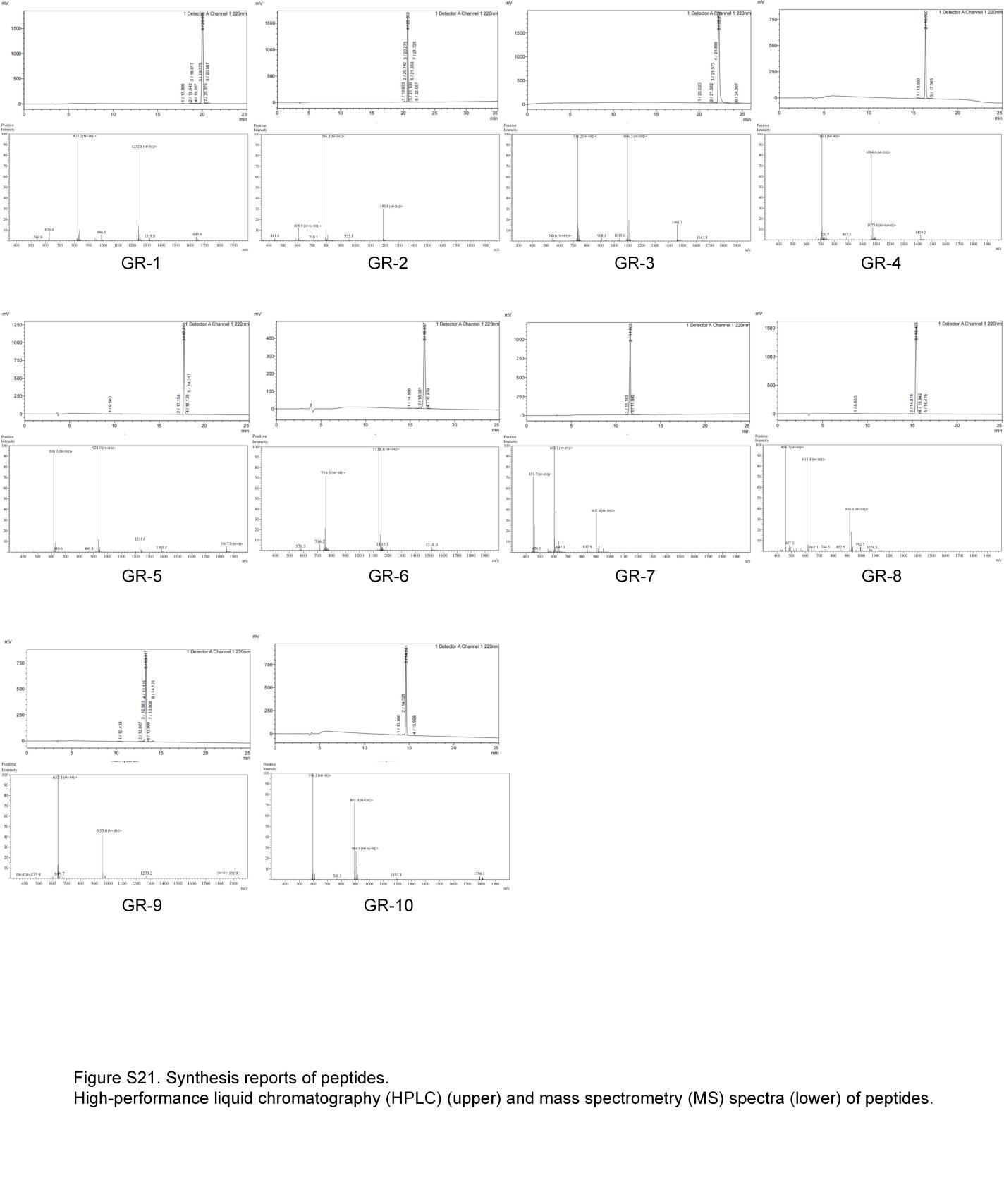


**Figure S23.Validation of synthesized peptide GR1–10.**

Up, High-performance liquid chromatography (HPLC). Down, Mass spectrometry (MS).


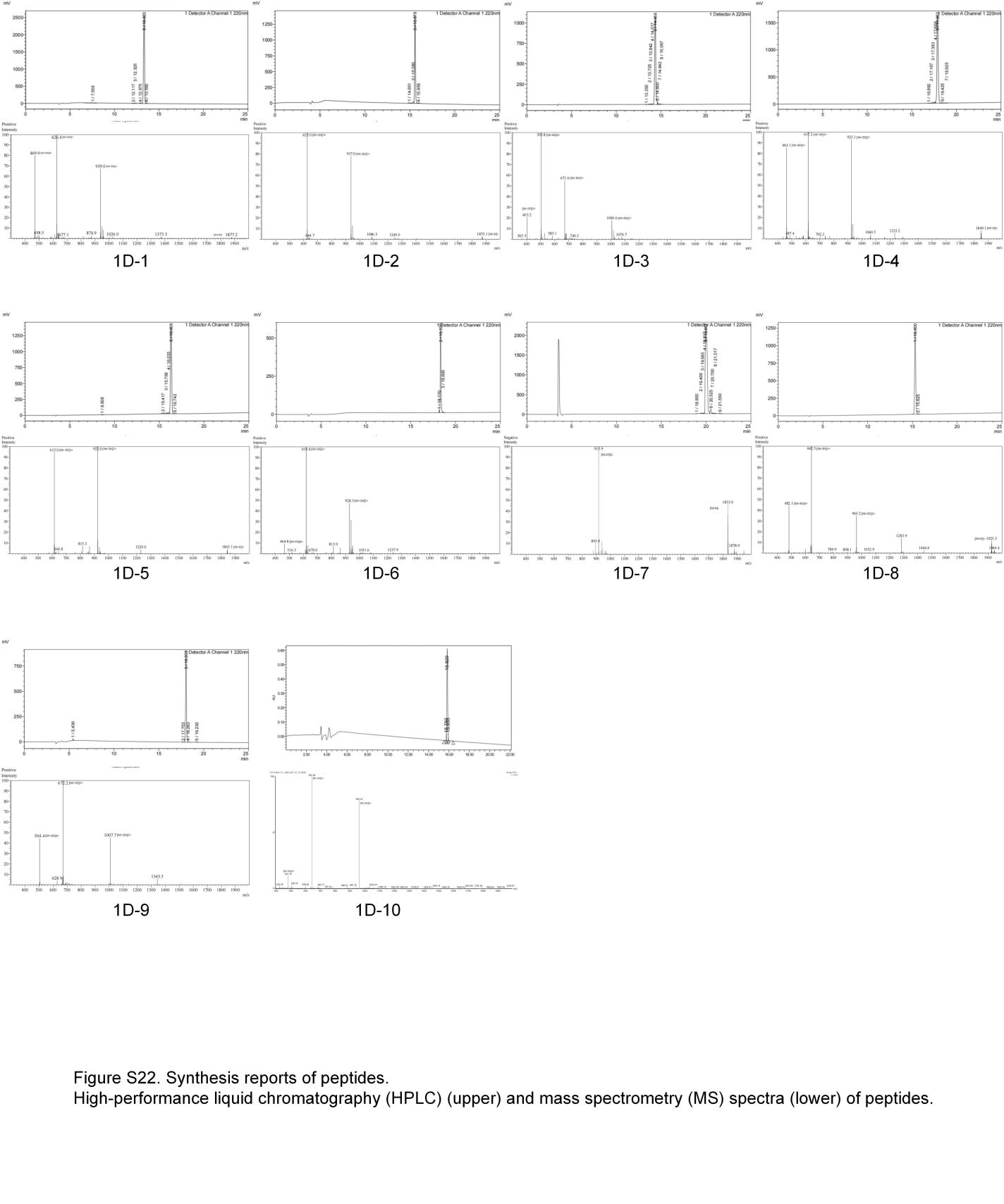


**Figure S24.Validation of synthesized peptide 1D1–10.**

Up, High-performance liquid chromatography (HPLC). Down, Mass spectrometry (MS).


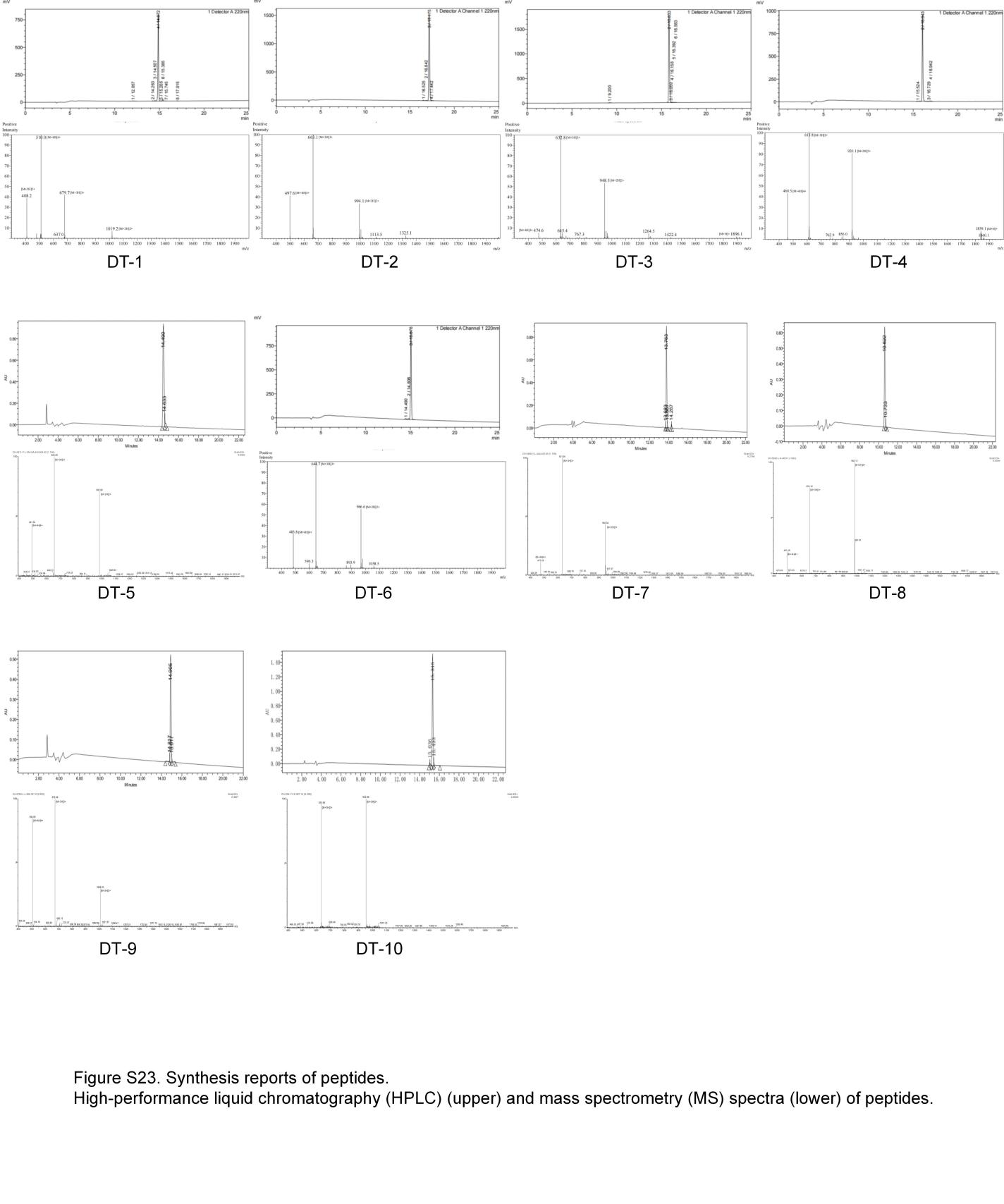


**Figure S25.Validation of synthesized peptide DT1–10.**

Up, High-performance liquid chromatography (HPLC). Down, Mass spectrometry (MS).

**Table S1. Performance comparison of four structure prediction methods on a wet-lab validated peptide dataset (79 PDBs, length < 50 aa).**

**Explanatory note:** Values are expressed as mean ± standard deviation (Mean ± SD).

| **Method** | **TM-score** | **RMSD** |
| --- | --- | --- |
| **AlphaFold2 with MSA** | 0.675±0.141 | 1.660±0.726 |
| **AlphaFold2 without MSA** | 0.533±0.156 | 3.621±2.729 |
| **ESMFold** | 0.666±0.148 | 1.712±0.853 |
| **HelixFold** | 0.645±0.202 | 1.627±0.856 |

**Table S2. Normalized values of atomic features and amino acid features for the 3D voxel coloring method.**

|  | **Atomic name** | **Abbreviation** | **Van der Waals radii** | **Relative atomic mass** |
| --- | --- | --- | --- | --- |
| **Atomic features** | Hydrogen | H | 1 | 1 |
|  | Carbon | C | 1.5 | 12 |
|  | Nitrogen | N | 1.5 | 14 |
|  | Oxygen | O | 1.5 | 16 |
|  | Sulfur | S | 2 | 30 |
|  | **Amino acid name** | **Abbreviation** | **Solubility** | **Charge** |
| **Amino acid features** | Alanine | A | 255 | 155 |
|  | Valine | V | 255 | 155 |
|  | Proline | P | 255 | 155 |
|  | Phenylalanine | F | 255 | 155 |
|  | Tryptophan | W | 255 | 155 |
|  | Isoleucine | I | 255 | 155 |
|  | Leucine | L | 255 | 155 |
|  | Glycine | G | 155 | 155 |
|  | Methionine | M | 155 | 155 |
|  | Tyrosine | Y | 55 | 155 |
|  | Serine | S | 55 | 155 |
|  | Threonine | T | 55 | 155 |
|  | Cysteine | C | 55 | 155 |
|  | Asparagine | N | 55 | 155 |
|  | Glutamine | Q | 55 | 155 |
|  | Aspartic acid | D | 55 | 55 |
|  | Glutamate | E | 55 | 55 |
|  | Lysine | K | 55 | 255 |
|  | Arginine | R | 55 | 255 |
|  | Histidine | H | 55 | 255 |

**Table S3. AP, F1, ACC, and AUC metrics on multilabel classification results of AMP inhibition activity against six types of drug-resistant bacteria.**

**Explanatory Note:** Values indicate mean ± SD (5-fold cross-validation). Bold underlining signifies statistical superiority over comparators. Total scores represent the sum of normalized metrics.

| **Method** | **AP** | **F1** | **ACC** | **AUC** | **Total score** |
| --- | --- | --- | --- | --- | --- |
| **3D voxel coloring+**  **Res-Conv Net** | 0.260±0.004 | **0.338**±0.006 | 0.622±0.007 | 0.591±0.009 | **394.04** |
| **3D voxel coloring+**  **SwinUNETR Net** | 0.211±0.011 | 0.251±0.004 | 0.630±0.002 | 0.511±0.013 | 338.32 |
| **GAT** | 0.251±0.004 | 0.291±0.004 | 0.631±0.003 | 0.578±0.008 | 376.18 |
| **GCN** | 0.269±0.006 | 0.306±0.005 | 0.628±0.006 | 0.597±0.006 | 390.22 |
| **Graphsage** | 0.258±0.005 | 0.305±0.002 | 0.628±0.006 | 0.589±0.008 | 384.35 |

**Table S4. AP, F1, ACC, and AUC metrics on four antimicrobial mechanisms of AMPs.**

**Explanatory Note:** Values indicate mean ± SD (5-fold cross-validation). Bold underlining signifies statistical superiority over comparators. Total scores represent the sum of normalized metrics.

| **Method** | **AP** | **F1** | **ACC** | **AUC** | **Total score** |
| --- | --- | --- | --- | --- | --- |
| **3D voxel coloring+**  **Res-Conv Net** | **0.400**±0.018 | **0.422**±0.011 | **0.911**±0.023 | 0.654±0.008 | **389.98** |
| **3D voxel coloring+**  **SwinUNETR Net** | 0.235±0.003 | 0.231±0.000 | 0.548±0.001 | 0.532±0.023 | 246.82 |
| **GAT** | 0.228±0.017 | 0.204±0.008 | 0.589±0.014 | 0.708±0.011 | 267.54 |
| **GCN** | 0.231±0.026 | 0.201±0.020 | 0.571±0.024 | **0.727**±0.013 | 268.11 |
| **Graphsage** | 0.244±0.029 | 0.215±0.015 | 0.578±0.016 | 0.718±0.005 | 274.21 |

**Table S5. AP, F1, ACC, and AUC metrics on multi-abel classification of six organ toxicities of AMPs.**

**Explanatory Note:** Values indicate mean ± SD (5-fold cross-validation). Bold underlining signifies statistical superiority over comparators. Total scores represent the sum of normalized metrics.

| **Method** | **AP** | **F1** | **ACC** | **AUC** | **Total score** |
| --- | --- | --- | --- | --- | --- |
| **3D voxel coloring+**  **Res-Conv Net** | 0.121±0.043 | **0.147**±0.019 | 0.607±0.006 | **0.728**±0.031 | **358.39** |
| **3D voxel coloring+**  **SwinUNETR Net** | 0.047±0.01 | 0.056±0.003 | **0.694**±0.002 | 0.551±0.079 | 241.73 |
| **GAT** | 0.126±0.010 | 0.080±0.007 | 0.571±0.019 | 0.672±0.060 | 302.85 |
| **GCN** | 0.171±0.024 | 0.091±0.010 | 0.545±0.039 | 0.706±0.078 | 337.73 |
| **Graphsage** | 0.124±0.019 | 0.079±0.007 | 0.566±0.024 | 0.684±0.068 | 302.34 |

**Table S6. AUC performance comparison across SVM, XGBoost, CatBoost, and M3CAD.**

**Explanatory note:** Values are expressed as mean ± standard deviation (Mean ± SD).

| **Model** | **AUC** |
| --- | --- |
| **SVM** | 63.64±0.85 |
| **XGBoost** | 63.17±0.53 |
| **CatBoost** | 71.33±1.79 |
| **M3CAD** | 76.32±0.89 |

**Table S7. Comparison of multimodal models vs. single-modality (sequence-only or 3D structure-only) performance on three downstream tasks.**

**Explanatory Note:** Values indicate mean ± SD (5-fold cross-validation). Bold underlining signifies statistical superiority over comparators. Total scores represent the sum of normalized metrics.

| **Task** | **Method** | **AP** | **F1** | **ACC** | **AUC** |
| --- | --- | --- | --- | --- | --- |
| **Antimicrobial activity** | Sequence | 0.375±0.013 | 0.432±0.013 | 0.644±0.024 | 0.691±0.010 |
|  | 3D structure | 0.260±0.004 | 0.338±0.006 | 0.622±0.007 | 0.591±0.009 |
|  | Multimodality | **0.396**±0.005 | **0.453**±0.005 | **0.681**±0.007 | **0.705**±0.006 |
| **Mechanism** | Sequence | 0.457±0.017 | 0.472±0.010 | 0.812±0.020 | 0.688±0.024 |
|  | 3D structure | 0.400±0.018 | 0.422±0.011 | 0.911±0.023 | 0.654±0.008 |
|  | Multimodality | **0.480**±0.022 | **0.536**±0.014 | **0.925**±0.008 | **0.709**±0.021 |
| **Toxic** | Sequence | 0.213±0.034 | 0.172±0.028 | 0.661±0.010 | 0.768±0.024 |
|  | 3D structure | 0.121±0.043 | 0.147±0.019 | 0.607±0.006 | 0.728±0.031 |
|  | Multimodality | 0.209±0.057 | **0.182**±0.036 | **0.677**±0.008 | **0.773**±0.019 |

**Table S8. Single-task (ST) vs multitask (MT) M3CAD performance across AP, F1, ACC, and AUC for bacterial classes.**

**Explanatory Note:** Values indicate mean ± SD (5-fold cross-validation). Bold underlining signifies statistical superiority over comparators. Total scores represent the sum of normalized metrics.

| **Category** | **Single-task (ST)** | | | | **M3CAD (MT)** | | | |
| --- | --- | --- | --- | --- | --- | --- | --- | --- |
|  | **AP** | **F1** | **ACC** | **AUC** | **AP** | **F1** | **ACC** | **AUC** |
| ***E. faecalis*** | 0.206 ±0.038 | 0.221  ±0.020 | 0.878  ±0.022 | 0.679  ±0.022 | 0.298  ±0.043 | 0.359  ±0.035 | 0.948  ±0.002 | 0.707  ±0.021 |
| ***P. aeruginosa*** | 0.627  ±0.007 | 0.562  ±0.003 | 0.474  ±0.016 | 0.739  ±0.011 | 0.673  ±0.010 | 0.627  ±0.004 | 0.740  ±0.010 | 0.770  ±0.004 |
| ***S. aureus*** | 0.603  ±0.009 | 0.551  ±0.019 | 0.503  ±0.040 | 0.737  ±0.009 | 0.635  ±0.018 | 0.597  ±0.015 | 0.744  ±0.008 | 0.757  ±0.008 |
| ***A. baumannii*** | 0.236  ±0.055 | 0.231  ±0.041 | 0.833  ±0.043 | 0.714  ±0.034 | 0.396  ±0.012 | 0.430  ±0.027 | 0.942  ±0.005 | 0.755  ±0.022 |
| **Enterobacteriaceae** | 0.584  ±0.009 | 0.522  ±0.010 | 0.466  ±0.025 | 0.732  ±0.010 | 0.640  ±0.021 | 0.597  ±0.015 | 0.754  ±0.007 | 0.762  ±0.005 |
| ***Salmonella*** | 0.365  ±0.030 | 0.313  ±0.019 | 0.660  ±0.029 | 0.726  ±0.030 | 0.500  ±0.032 | 0.505  ±0.027 | 0.897  ±0.008 | 0.786  ±0.018 |
| **Arithmetic Mean** | 0.437  ±0.178 | 0.400  ±0.150 | 0.636  ±0.171 | 0.721  ±0.030 | 0.524  ±0.141 | 0.519  ±0.101 | 0.838  ±0.093 | 0.756  ±0.029 |

**Table S9. Physicochemical properties of antimicrobial peptide QLX-3DV-1 and QLX-3DV-2, and similarity to training AMPs.**

|  | **QLX-3DV-1** | **QLX-3DV-2** |
| --- | --- | --- |
| **Hydrophobic** | -1.329 | -0.971 |
| **Hydrophobic moment** | 19.976 | 21.134 |
| **Aliphatic index** | 14.286 | 21.429 |
| **Aromaticity** | 0.286 | 0.286 |
| **Alpha helix** | 0.429 | 0.5 |
| **Sequence similarity** | 0.457 | 0.457 |
| **Beta helix** | 0.143 | 0.071 |
| **Turn helix** | 0.071 | 0.071 |
| **Charge** | 5.76 | 5.76 |
| **Charge density** | 0.411 | 0.411 |
| **Instability index** | 59.521 | 45.764 |

**Table S10. Experimental MICs of randomly sampled 10 peptides from m2cVAE, the top 10 c_AMPs selected from the regression module and complete pipeline M3CAD-selected 10 c_AMPs (3 biologically independent replicates).**

| **Method** | **Name** | **MIC (μg/mL)** | | |
| --- | --- | --- | --- | --- |
|  |  | **S. aureus CMCC26003** | **E.coli CMCC44102** | **A. baumannii 106** |
| Randomly sampled  10 peptides  from m2cVAE (Generation) | G-1 | >256 | >256 | >256 |
|  | G-2 | >256 | >256 | >256 |
|  | G-3 | >256 | >256 | >256 |
|  | G-4 | >256 | >256 | >256 |
|  | G-5 | >256 | >256 | >256 |
|  | G-6 | >256 | >256 | >256 |
|  | G-7 | 256 | 128 | 256 |
|  | G-8 | >256 | >256 | 256 |
|  | G-9 | >256 | >256 | >256 |
|  | G-10 | 256 | >256 | >256 |
| Regression moudle-selected 10 c_AMPs (Generation+Regression) | GR-1 | >256 | >256 | >256 |
|  | GR-2 | >256 | >256 | >256 |
|  | GR-3 | >256 | >256 | >256 |
|  | GR-4 | >256 | >256 | >256 |
|  | GR-5 | >256 | >256 | >256 |
|  | GR-6 | >256 | >256 | >256 |
|  | GR-7 | 64 | 32 | 64 |
|  | GR-8 | 32 | 32 | 128 |
|  | GR-9 | 64 | 128 | 128 |
|  | GR-10 | 16 | 32 | 32 |
| Complete pipeline M3CAD-selected  10 c_AMPs (Generation+Regression+Classification) | QLX-3DV-1 | 8 | 8 | 8 |
|  | QLX-3DV-2 | 8 | 8 | 8 |
|  | QLX-3DV-3 | 8 | 16 | 8 |
|  | QLX-3DV-4 | 8 | 8 | 16 |
|  | QLX-3DV-5 | 8 | 16 | 8 |
|  | QLX-3DV-6 | 8 | 16 | 16 |
|  | QLX-3DV-7 | 8 | 16 | 16 |
|  | QLX-3DV-8 | 8 | 16 | 16 |
|  | QLX-3DV-9 | 8 | 16 | 16 |
|  | QLX-3DV-10 | 8 | 16 | 16 |

**Table S11. Experimental MICs of the top 10 c_AMP selected from the pipeline trained with 3D structural characteristics and sequence, and the top 10 c_AMP selected from the pipeline trained with only sequence (3 biologically independent replicates).**

| **Method** | **Name** | **Sequence** | **MIC (μg/mL)** | | |
| --- | --- | --- | --- | --- | --- |
|  |  |  | 1. ***aureus* CMCC26003** | ***E.coli* CMCC44102** | ***A. baumannii* 106** |
| pipeline trained with 3D structural characteristics and sequence selected 10 c_AMPs | QLX-3DV-1 | KWKIKWPSRWFRKL-NH_2_ | 8 | 8 | 8 |
|  | QLX-3DV-2 | KWKIKWPVRWFRKL-NH_2_ | 8 | 8 | 8 |
|  | QLX-3DV-3 | KWKIKWPSNWFTKL-NH_2_ | 8 | 16 | 8 |
|  | QLX-3DV-4 | KWKIKWPSRWFTKL-NH_2_ | 8 | 8 | 16 |
|  | QLX-3DV-5 | KWKIKRPVKWFTML-NH_2_ | 8 | 16 | 8 |
|  | QLX-3DV-6 | KWKIKQWVRWFRKL-NH_2_ | 8 | 16 | 16 |
|  | QLX-3DV-7 | KRKIKWKVRWFTYL-NH_2_ | 8 | 16 | 16 |
|  | QLX-3DV-8 | KRKIKWWVRWITKL-NH_2_ | 8 | 16 | 16 |
|  | QLX-3DV-9 | KWKIKWRVRWFKLL-NH_2_ | 8 | 16 | 16 |
|  | QLX-3DV-10 | KWKIKWHVRWFRVL-NH_2_ | 8 | 16 | 16 |
|  | **Geometric Mean** | | **8.0** | **13.0** | **12.1** |
| pipeline trained with only sequence selected 10 c_AMPs | 1D-1 | KWKIKWPSNWFTKK-NH_2_ | 16 | 32 | 16 |
|  | 1D-2 | KVMIKWPSYWFTMR-NH_2_ | 16 | 16 | 32 |
|  | 1D-3 | KYKIKWPYRWFRKL-NH_2_ | 16 | 8 | 16 |
|  | 1D-4 | KWMIKTPSHWFTMR-NH_2_ | 128 | 64 | 256 |
|  | 1D-5 | KWMIKWPSHWFTVP-NH_2_ | 32 | 64 | 32 |
|  | 1D-6 | KHMIKWPRNWFTVL-NH_2_ | 32 | 32 | 32 |
|  | 1D-7 | KWMIIWPSKWFTVL-NH_2_ | 16 | 256 | 32 |
|  | 1D-8 | KWMIKWKTKWFVML-NH_2_ | 128 | 256 | 128 |
|  | 1D-9 | KWMIKWPLKWIIMKL-NH_2_ | 64 | 64 | 128 |
|  | 1D-10 | KRMIKWRVKWFTML-NH_2_ | 16 | 32 | 16 |
|  | **Geometric Mean** | | **32.0** | **48.5** | **42.2** |

**Table S12. Experimental MICs** **and HC50 value of the top 10 c_AMP selected from the triple-task classifier that includes mechanism, antimicrobial activity, and toxicity, and the top 10 c_AMP selected from the double-task classifier that includes antimicrobial activity and toxicity (3 biologically independent replicates).**

| **Method** | **Name** | **Sequence** | **MIC (μg/mL)** | | | **HC_50_**  **(μg/ml)** |
| --- | --- | --- | --- | --- | --- | --- |
|  |  |  | ***S. aureus* CMCC26003** | ***E.coli* CMCC44102** | ***A. baumannii* 106** |  |
| triple-task classifier that includes mechanism, antimicrobial activity and toxicity selected 10 c_AMPs | QLX-3DV-1 | KWKIKWPSRWFRKL-NH_2_ | 8 | 8 | 8 | >2048 |
|  | QLX-3DV-2 | KWKIKWPVRWFRKL-NH_2_ | 8 | 8 | 8 | >2048 |
|  | QLX-3DV-3 | KWKIKWPSNWFTKL-NH_2_ | 8 | 16 | 8 | >2048 |
|  | QLX-3DV-4 | KWKIKWPSRWFTKL-NH_2_ | 8 | 8 | 16 | >2048 |
|  | QLX-3DV-5 | KWKIKRPVKWFTML-NH_2_ | 8 | 16 | 8 | >2048 |
|  | QLX-3DV-6 | KWKIKQWVRWFRKL-NH_2_ | 8 | 16 | 16 | >512 |
|  | QLX-3DV-7 | KRKIKWKVRWFTYL-NH_2_ | 8 | 16 | 16 | >2048 |
|  | QLX-3DV-8 | KRKIKWWVRWITKL-NH_2_ | 8 | 16 | 16 | >256 |
|  | QLX-3DV-9 | KWKIKWRVRWFKLL-NH_2_ | 8 | 16 | 16 | >1024 |
|  | QLX-3DV-10 | KWKIKWHVRWFRVL-NH_2_ | 8 | 16 | 16 | >1024 |
| double-task classifier that includes antimicrobial activity and toxicity | DT-1 | KWKIKWVYRWFRKL-NH_2_ | 8 | 8 | 8 | <256 |
|  | DT-2 | KWMIKWPSKWFRMY-NH_2_ | 8 | 8 | 16 | >2048 |
|  | DT-3 | KWKIKWKSNWFTML-NH_2_ | 8 | 8 | 16 | >2048 |
|  | DT-4 | KWMIKWPSKWCSMK-NH_2_ | 8 | 16 | 16 | >1024 |
|  | DT-5 | KWMIKWRVKWRTML-NH_2_ | 16 | 16 | 8 | >512 |
|  | DT-6 | KWMIKWPSHWFVMR-NH_2_ | 8 | 16 | 32 | >512 |
|  | DT-7 | KWMIKWVSKWRTKL-NH_2_ | 32 | 16 | 8 | >2048 |
|  | DT-8 | KWMIKWVSKWKRML-NH_2_ | 16 | 8 | 16 | >1024 |
|  | DT-9 | KWKIKWRVRWFRIL-NH_2_ | 16 | 32 | 16 | >1024 |
|  | DT-10 | KWMHKWPSKWFVMI-NH_2_ | 16 | 32 | 16 | >1024 |

**Table S13. Physicochemical properties of training AMPs and newly discovered AMPs using the M3-CAD pipeline.**

**Explanatory note:** Values are expressed as mean ± standard deviation (Mean ± SD).

|  | **Training AMPs in QLAPD (n=9,392)** | **M3-CAD AMPs (n=1,000)** |
| --- | --- | --- |
| **Hydrophobic** | -0.289191±1.051384 | -0.297807±0.787882 |
| **Hydrophobic moment** | 19.336517±12.156072 | 10.311879±5.527324 |
| **Aliphatic index** | 31.238395±16.324402 | 25.580347±11.353217 |
| **Aromaticity** | 0.124672±0.117457 | 0.143548±0.090831 |
| **Alpha helix** | 0.372594±0.220745 | 0.227345±0.108067 |
| **Beta helix** | 0.180349±0.133597 | 0.229516±0.109581 |
| **Turn helix** | 0.393074±0.138374 | 0.396567±0.126287 |
| **Charge** | 3.766448±3.122441 | 1.663175±1.49528 |
| **Charge density** | 0.219823±0.175974 | 0.109502±0.098301 |
| **Instability index** | 40.070508±58.130655 | 39.16531±33.673232 |

**Table S14. Therapeutic index (CC50/geometric mean MIC) of QLX-3DV-1–20, and SAAP-148 against various bacterial species on HEK-293T.**

| **Peptide** | **CC50 (μg/ml)** | **CC50 / MIC (Therapeutic index)** | | | | | | | |
| --- | --- | --- | --- | --- | --- | --- | --- | --- | --- |
|  |  | ***S. aureus*** | ***E. faecium*** | ***E.coli*** | ***Salmonella typhi*** | ***A. baumannii*** | ***K. pneumoniae*** | ***P. aeruginosa*** | **all species** |
| QLX-3DV-1 | 309.29 | 44.41 | 54.68 | 32.51 | 38.66 | 45.98 | 19.33 | 45.98 | 40.87 |
| QLX-3DV-2 | 255.79 | 31.97 | 45.22 | 26.89 | 31.97 | 31.97 | 15.99 | 31.97 | 31.1 |
| QLX-2DV-3 | 536.3 | 19.25 | 47.4 | 39.86 | 23.7 | 56.37 | 33.52 | 23.7 | 32.6 |
| QLX-2DV-4 | 740.81 | 40.31 | 77.87 | 55.06 | 32.74 | 38.93 | 46.3 | 27.53 | 43.8 |
| QLX-2DV-5 | 498.34 | 11.8 | 22.02 | 52.38 | 44.05 | 62.29 | 62.29 | 15.57 | 28.66 |
| QLX-2DV-6 | 193.88 | 13.92 | 28.82 | 24.24 | 12.12 | 14.41 | 12.12 | 6.06 | 14.71 |
| QLX-2DV-7 | 492.24 | 35.34 | 51.74 | 25.87 | 30.77 | 30.77 | 30.77 | 21.75 | 31.63 |
| QLX-2DV-8 | 176.95 | 16.76 | 31.28 | 13.15 | 11.06 | 11.06 | 11.06 | 13.15 | 15 |
| QLX-2DV-9 | 310.94 | 33.84 | 109.93 | 27.48 | 38.87 | 27.48 | 19.43 | 27.48 | 35.77 |
| QLX-2DV-10 | 294.77 | 21.16 | 36.85 | 13.03 | 18.42 | 18.42 | 18.42 | 10.95 | 18.42 |
| QLX-2DV-11 | 399.47 | 57.36 | 99.87 | 83.98 | 49.93 | 70.62 | 35.31 | 29.69 | 58.97 |
| QLX-2DV-12 | 536.56 | 38.52 | 189.7 | 67.07 | 33.54 | 39.88 | 33.54 | 67.07 | 58.39 |
| QLX-2DV-13 | >2048 | >111.43 | >861.08 | >256 | >90.51 | >215.27 | >64 | >181.02 | >199.47 |
| QLX-2DV-14 | 87.28 | 7.2 | 15.43 | 10.91 | 10.91 | 9.17 | 7.71 | 4.59 | 8.74 |
| QLX-2DV-15 | 2015.86 | 190.97 | 503.97 | 251.98 | 89.09 | 178.18 | 63 | 89.09 | 175.73 |
| QLX-2DV-16 | 482.22 | 8.66 | 42.62 | 42.62 | 15.07 | 60.28 | 10.66 | 15.07 | 22.84 |
| QLX-2DV-17 | >2048 | >97.01 | >181.02 | >152.22 | >32 | >107.63 | >32 | >53.82 | >89.26 |
| QLX-2DV-18 | 91.85 | 3.3 | 11.48 | 5.74 | 1.44 | 2.87 | 2.03 | 2.03 | 3.58 |
| QLX-2DV-19 | 1035.79 | 18.59 | 76.99 | 45.78 | 32.37 | 64.74 | 16.18 | 32.37 | 37.18 |
| QLX-2DV-20 | >2048 | >27.86 | >38.05 | >107.63 | >45.25 | >53.82 | >22.63 | >26.91 | >41.07 |
| SAAP-148 | 42.2 | 1.51 | 5.28 | 1.11 | 1.32 | 1.32 | 1.32 | 1.32 | 1.65 |

**Table S15. Therapeutic index (CC50/geometric mean MIC) of QLX-3DV-1–20, and SAAP-148 against various bacterial species on MHCC97-H.**

| **Peptide** | **CC50  (μg/ml)** | **CC50 / MIC (Therapeutic index)** | | | | | | | | |
| --- | --- | --- | --- | --- | --- | --- | --- | --- | --- | --- |
|  |  | ***S. aureus*** | ***E. faecium*** | ***E.coli*** | ***Salmonella typhi*** | ***A. baumannii*** | ***K. pneumoniae*** | ***P. aeruginosa*** | | **all species** |
| QLX-3DV-1 | 275.02 | 39.49 | 48.62 | 28.91 | 34.38 | 40.88 | 17.19 | 40.88 | 36.34 | |
| QLX-3DV-2 | 437.34 | 54.67 | 77.31 | 45.97 | 54.67 | 54.67 | 27.33 | 54.67 | 53.17 | |
| QLX-3DV-3 | 574.38 | 20.62 | 50.77 | 42.69 | 25.38 | 60.37 | 35.9 | 25.38 | 34.92 | |
| QLX-3DV-4 | 573.57 | 31.21 | 60.29 | 42.63 | 25.35 | 30.14 | 35.85 | 21.32 | 33.91 | |
| QLX-3DV-5 | 465.99 | 11.04 | 20.59 | 48.98 | 41.19 | 58.25 | 58.25 | 14.56 | 26.8 | |
| QLX-3DV-6 | 230.69 | 16.56 | 34.29 | 28.84 | 14.42 | 17.15 | 14.42 | 7.21 | 17.51 | |
| QLX-3DV-7 | 428.07 | 30.73 | 45 | 22.5 | 26.75 | 26.75 | 26.75 | 18.92 | 27.51 | |
| QLX-3DV-8 | 210.11 | 19.9 | 37.14 | 15.62 | 13.13 | 13.13 | 13.13 | 15.62 | 17.81 | |
| QLX-3DV-9 | 334.39 | 36.39 | 118.22 | 29.56 | 41.8 | 29.56 | 20.9 | 29.56 | 38.46 | |
| QLX-3DV-10 | 307.1 | 22.05 | 38.39 | 13.57 | 19.19 | 19.19 | 19.19 | 11.41 | 19.19 | |
| QLX-3DV-11 | 378.87 | 54.4 | 94.72 | 79.65 | 47.36 | 66.98 | 33.49 | 28.16 | 55.93 | |
| QLX-3DV-12 | 537.79 | 38.61 | 190.14 | 67.22 | 33.61 | 39.97 | 33.61 | 67.22 | 58.52 | |
| QLX-3DV-13 | 1399.87 | 76.17 | 588.57 | 174.98 | 61.87 | 147.14 | 43.75 | 123.73 | 136.34 | |
| QLX-3DV-14 | 98.69 | 8.14 | 17.45 | 12.34 | 12.34 | 10.37 | 8.72 | 5.19 | 9.88 | |
| QLX-3DV-15 | 1557.92 | 147.59 | 389.48 | 194.74 | 68.85 | 137.7 | 48.69 | 68.85 | 135.81 | |
| QLX-3DV-16 | 500.96 | 8.99 | 44.28 | 44.28 | 15.66 | 62.62 | 11.07 | 15.66 | 23.73 | |
| QLX-3DV-17 | 1638.43 | 77.61 | 144.82 | 121.78 | 25.6 | 86.11 | 25.6 | 43.05 | 71.41 | |
| QLX-3DV-18 | 92.31 | 3.31 | 11.54 | 5.77 | 1.44 | 2.88 | 2.04 | 2.04 | 3.6 | |
| QLX-3DV-19 | 1486.53 | 26.68 | 110.49 | 65.7 | 46.45 | 92.91 | 23.23 | 46.45 | 53.36 | |
| QLX-3DV-20 | >2048 | >27.86 | >38.05 | >107.63 | >45.25 | >53.82 | >22.63 | >26.91 | >41.07 | |
| SAAP-148 | 26.79 | 0.96 | 3.35 | 0.7 | 0.84 | 0.84 | 0.84 | 0.84 | 1.05 | |

**Table S16. MICs of AMPs and cefepime (tested alone or in combination) and FICIs for the combinations of AMPs and cefepime.**

**Explanatory note:** FICI represents Fractional Inhibitory Concentration Indices. FICI values were interpreted as follows: FICI ≤ 0.5 (synergy); 0.5 < FICI ≤ 4.0 (no interaction), and FICI > 4.0 (antagonism). (A), alone; (C), combination, and (S), synergism.

| **QLX-3DV-1 (μg/mL)** | | **cefepime (μg/mL)** | | **FICI** |
| --- | --- | --- | --- | --- |
| MIC (A) | MIC (C) | MIC (A) | MIC (C) |  |
| 32 | 8 | 64 | 4 | 0.3125 (S) |

| **QLX-3DV-2 (μg/mL)** | | **cefepime (μg/mL)** | | **FICI** |
| --- | --- | --- | --- | --- |
| MIC (A) | MIC (C) | MIC (A) | MIC (C) |  |
| 32 | 8 | 128 | 8 | 0.1875 (S) |

**Table S17. Sequences of *de novo* designed peptides QLX-3DV-1–20 by M3CAD.**

| **Name** | **Sequence** |
| --- | --- |
| QLX-3DV-11 | RRQKWRILALNIWK-NH_2_ |
| QLX-3DV-12 | VKVFRFYWKRKQRF-NH_2_ |
| QLX-3DV-13 | QYRLVWQRRLLRRRFLLKA-NH_2_ |
| QLX-3DV-14 | RQYKRWRKFVRPWWKFLKD-NH_2_ |
| QLX-3DV-15 | KVINYKLRFFWAPWFKKL-NH_2_ |
| QLX-3DV-16 | RRALIYNLVKKWAYSYSAT-NH_2_ |
| QLX-3DV-17 | LAVLIFVRRKQL-NH_2_ |
| QLX-3DV-18 | YQQRAWKLPWILRFLRL-NH_2_ |
| QLX-3DV-19 | IVIRFLKPKLLKALK-NH_2_ |
| QLX-3DV-20 | IRPLVQPRYRFYPRKRL-NH_2_ |

**Table S18. Sequences of contrast peptides.**

| **Name** | **Sequence** |
| --- | --- |
| QLX-227-1 | KWMIKWPSNWFTML-NH_2_ |
| LL-37 | LLGDFFRKSKEKIGKEFKRIVQRIKDFLRNLVPRTES |
| SAAP-148 | Ac-LKRVWKRVFKLLKRYWRQLKKPVR-NH_2_ |
| C16G2 | TFFRLFNRSFTQALGKGGGKNLRIIRKGIHIIKKY-NH_2_ |
| Omiganan | Ac-ILRWPWWPWRRK-NH_2_ |
| OP-145 | IGKEFKRIVERIKRFLRELVRPLR-NH_2_ |
| DPK-060 | GKHKNKGKKNGKHNGWKWWW |

**Table S19. Sequences of Randomly sampled 10 peptides from m2cVAE(Generation).**

| **Name** | **Sequence** |
| --- | --- |
| G-1 | KWNPKWFSQWFTMIVTWP-NH_2_ |
| G-2 | KWNPKWPWNYFTCLACWI-NH_2_ |
| G-3 | RSMPKWPWNWPTMIVCWIM-NH_2_ |
| G-4 | KSMPKWPWNWPCMIAMYF-NH_2_ |
| G-5 | KFMIKWFCEYWTML-NH_2_ |
| G-6 | KWMIKKPSNWPTMP-NH_2_ |
| G-7 | KWMKKWPSNWPTMR-NH_2_ |
| G-8 | KWMIKRPSNWPTMR-NH_2_ |
| G-9 | KWMIVWRSNWFTVL-NH_2_ |
| G-10 | KWMIKWITKWFVA-NH_2_ |

|  |
| --- |

**Table S20. Sequences of Regression moudle-selected 10 c_AMPs (Generation+Regression).**

| **Name** | **Sequence** |
| --- | --- |
| GR-1 | KWMPKWPWNWPTMIVMWF-NH_2_ |
| GR-2 | KWNPKWFSMYFTCLVCWF-NH_2_ |
| GR-3 | KWMIKSFSQWFTMLACSP-NH_2_ |
| GR-4 | KSQIKSFTQFFSMLVCSF-NH_2_ |
| GR-5 | KYMPKWFSEWFSNP-NH_2_ |
| GR-6 | KWMIKWFSDWFTNIACSI-NH_2_ |
| GR-7 | KWKIKWPSNKFTKL-NH_2_ |
| GR-8 | KWKIKWPSNRFTKL-NH_2_ |
| GR-9 | KWMIKWPYNWKTMP-NH_2_ |
| GR-10 | KWMIKTPSKWFTMC-NH_2_ |
